# Correction: Determining soil particle-size distribution from infrared spectra using machine learning predictions: Methodology and modeling

**DOI:** 10.1371/journal.pone.0273277

**Published:** 2022-08-16

**Authors:** Elizabeth Jeanne Parent, Serge-Étienne Parent, Léon Etienne Parent

In the “Spectral data modeling” subsection of the “Results” section, there is an error in the fifth sentence of the first paragraph. The correct sentence is: “Combining laser and sedimentation methods in Set2 improved the clay predictions with R2 values of 0.80–0.85.”

In Fig 4, incorrect titles were used on the left-hand side to correspond to the values of the Mean *ilr* differences. The authors have provided a corrected version here.

**Fig 4:**
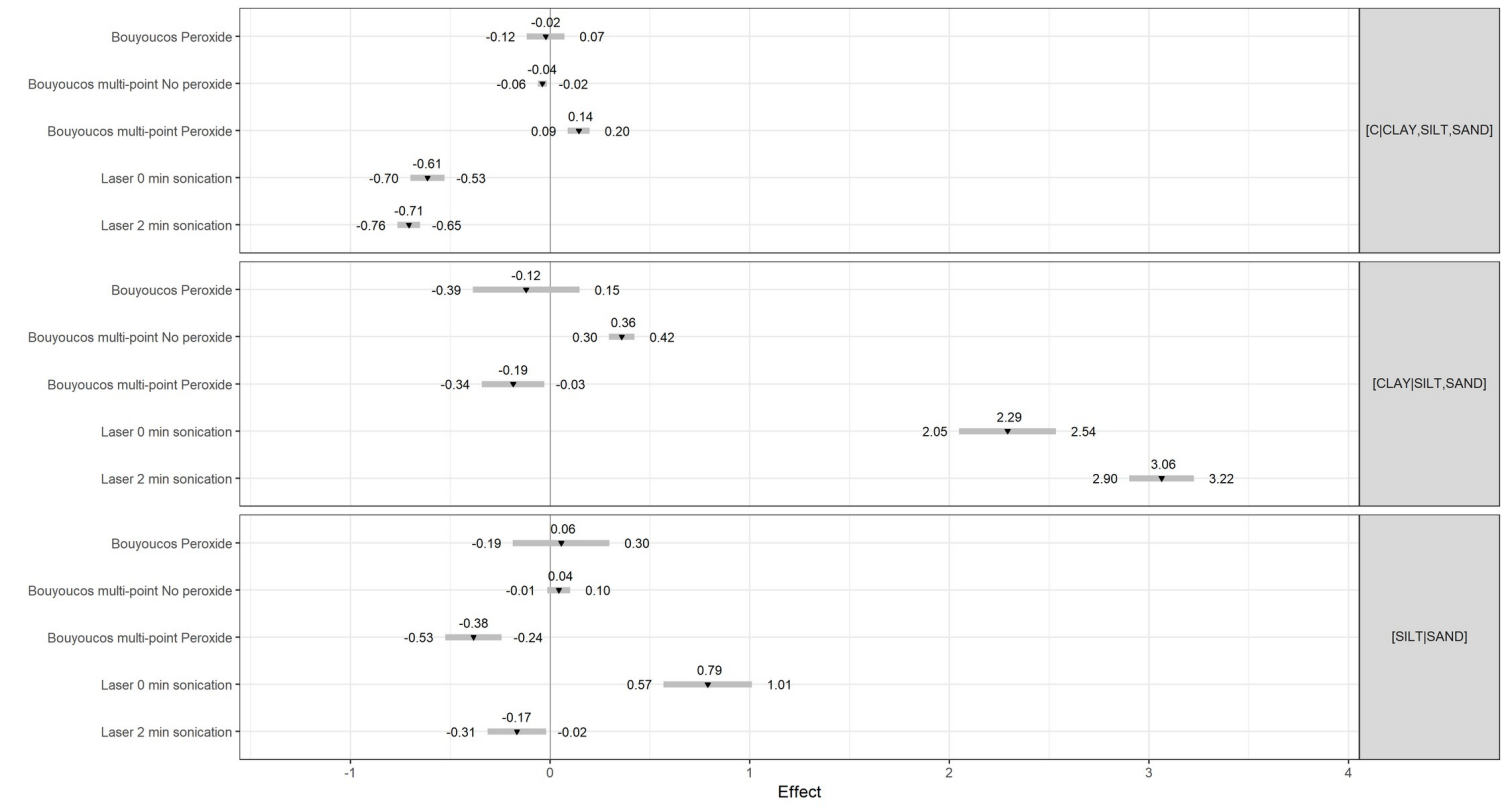


There are errors in Table 2. Please see the correct Table 2 here.

**Table 2:** Comparison of methods (method1 minus method2) using paired t-test and confidence intervals (p ≤ 0.05).

Target variable	Method1	Method2	p.value	N
[Carbon | Clay,Silt,Sand]	Sedimentation 2-point No peroxide	Sedimentation 2-point peroxide	ns	38
[Carbon | Clay,Silt,Sand]	Sedimentation 2-point No peroxide	Laser 0 min	****	46
[Carbon | Clay,Silt,Sand]	Sedimentation 2-point No peroxide	Laser 2 min	****	106
[Carbon | Clay,Silt,Sand]	Sedimentation 2-point No peroxide	Sedimentation multi-point No peroxide	****	763
[Carbon | Clay,Silt,Sand]	Sedimentation 2-point No peroxide	Sedimentation multi-point peroxide	****	206
[Clay | Silt,Sand]	Sedimentation 2-point No peroxide	Sedimentation 2-point peroxide	**	38
[Clay | Silt,Sand]	Sedimentation 2-point No peroxide	Laser 0 min	****	46
[Clay | Silt,Sand]	Sedimentation 2-point No peroxide	****	****	106
[Clay | Silt,Sand]	Sedimentation 2-point No peroxide	Sedimentation multi-point No peroxide	****	763
[Clay | Silt,Sand]	Sedimentation 2-point No peroxide	Sedimentation multi-point peroxide	ns	206
[Silt | Sand]	Sedimentation 2-point No peroxide	Sedimentation 2-point peroxide	ns	38
[Silt | Sand]	Sedimentation 2-point No peroxide	Laser 0 min	****	46
[Silt | Sand]	Sedimentation 2-point No peroxide	Laser 2 min	*	106
[Silt | Sand]	Sedimentation 2-point No peroxide	Sedimentation multi-point No peroxide	**	763
[Silt | Sand]	Sedimentation 2-point No peroxide	Sedimentation multi-point peroxide	*	206
Sand	Sedimentation 2-point No peroxide	Sieving	*	746
Sand	Sedimentation 2-point peroxide	Sieving	ns	26
Sand	Laser 0 min	Sieving	****	34
Sand	Laser 2 min	Sieving	*	77
Sand	Sedimentation multi-point No peroxide	Sieving	ns	358
Sand	Sedimentation multi-point peroxide	Sieving	****	84

ns: non-significant (> 0.05),

*: significant at p ≤ 0.05,

**: significant at p ≤ 0.01,

***: significant at p ≤ 0.005,

****: significant at p ≤ 0.001,

N: Sample size.
