# Supplementary material for: Determining soil particle-size distribution from infrared spectra using machine learning predictions: Methodology and modeling
Source: PLoS One. 2021 Jul 20;16(7):e0233242. doi: 10.1371/journal.pone.0233242 (PMC8291647; doi:10.1371/journal.pone.0233242)
Supplement: S1 Table — (DOCX) [file pone.0233242.s001.docx]

**S1 Table. Slope, intercept, and number of observations of models**

| **Set** | **Dependent**  **Variables** | **Independent variables** | **Laboratory method**  **for calibration** | **Sand** | **Silt** | **Clay** | **C** | **Sand** | Silt | Clay | C |
| --- | --- | --- | --- | --- | --- | --- | --- | --- | --- | --- | --- |
|  |  |  |  | **Slope** | | | | **Intercept** | | | |
| **Set1** | **Ilr** | **2X** | **Laser** | 1.08 | 1.06 | 0.77 | 0.66 | -3.29 | -3.01 | 0.08 | 0.76 |
|  | **%** |  |  | 1.03 | 1.10 | 1.08 | 1.66 | -3.02 | -3.24 | -0.47 | -0.47 |
| **Set2** | **Ilr** | **Lab method,**  **PT, NIR-2X** | **2-point 2-h sedimentation+ Laser** | 1.05 | 1.05 | 0.99 | 1.09 | -2.28 | 1.59 | -1.44 | -0.20 |
|  | **%** |  |  | 1.11 | 1.14 | 1.02 | 1.13 | -6.78 | -6.74 | -3.50 | -0.46 |
| **Set3** | **Ilr** | **NIR-2X** | **Multi-point 7-h sedimentation** | 1.01 | 0.89 | 1.20 | 1.02 | -4.39 | 5.08 | -0.94 | -0.02 |
|  |  |  | **2-point 2-h sedimentation** | 0.95 | 0.85 | 0.95 | 1.04 | 2.59 | 4.75 | 0.05 | -0.06 |
| **Set4** | **Ilr** | **MIR** | **2-point 2-h sedimentation** | 0.94 | 0.72 | 1.02 | 0.70 | 6.63 | 7.19 | -1.72 | 0.76 |
|  |  | **NIR-2X** |  | 0.93 | 0.67 | 0.96 | -0.35 | 6.81 | 9.78 | -0.79 | 3.37 |
| **Set5** | **Ilr** | **NIR-4X** | **2-point 2-h sedimentation** | 1.01 | 1.02 | 0.97 | 1.13 | 1.42 | -1.03 | -0.59 | -0.31 |
|  |  | **NIR-2X** |  | 1.00 | 1.01 | 0.93 | 1.03 | 3.81 | -1.31 | -0.94 | -0.17 |
| **Set6** | **Ilr** | **PT, NIR-2X** | **2-point 2-h sedimentation** | 1.00 | 1.00 | 0.96 | 1.21 | 0.30 | 0.34 | 0.32 | -0.49 |
|  | **%** |  |  | 1.05 | 1.09 | 1.04 | 1.14 | -3.93 | -2.08 | -0.69 | -0.39 |
|  | **Slope, Intercept** |  |  | 1.10 | 1.10 | 1.14 | - | -8.86 | -0.92 | -0.59 | - |
| **Set7** | **Ilr** | **All features,**  **NIR-2X** | **2-point 2-h sedimentation** | 0.99 | 0.91 | 1.06 | 0.82 | 4.85 | -1.18 | -2.15 | 0.29 |
|  |  | **No feature,**  **NIR-2X** |  | 1.04 | 1.08 | 1.00 | 1.45 | -0.69 | -2.53 | -0.97 | -1.15 |
|  |  | **Carbon, NIR-2X** |  | 1.03 | 1.07 | 1.03 | 1.68 | -0.06 | -2.73 | -1.49 | -1.93 |
|  |  | **Bulk density,**  **NIR-2X** |  | 1.05 | 1.01 | 0.96 | 1.13 | 0.58 | -1.11 | -0.19 | -0.41 |
|  |  | **pH, NIR-2X** |  | 1.02 | 0.98 | 0.99 | 1.83 | 1.04 | -0.57 | -0.25 | -2.14 |
|  |  | **Color, NIR-2X** |  | 1.01 | 1.01 | 1.03 | 1.49 | 0.78 | -1.68 | -0.84 | -1.35 |
|  |  | **Oxalate, NIR-2X** |  | 1.02 | 1.04 | 0.99 | 1.27 | 0.11 | -0.93 | -0.60 | -0.75 |
|  |  | **Mehlich3, NIR-2X** |  | 1.01 | 1.01 | 0.95 | 1.23 | 0.38 | -0.50 | 1.00 | -0.77 |
| **Set8** | **%** | **NIR-2X** | **Sand sieving** | 1.19 | - | - | - | -14.35 | - | - | - |
|  |  |  | **2-point 2-h sedimentation** | 1.07 | - | - | - | -5.95 | - | - | - |

C: Carbon; PT: Pre-treatments (no peroxide or peroxide); MIR: MIR scores; RMSE: root mean square error; N: Sample size
