# Supplementary material for: Determining soil particle-size distribution from infrared spectra using machine learning predictions: Methodology and modeling
Source: PLoS One. 2021 Jul 20;16(7):e0233242. doi: 10.1371/journal.pone.0233242 (PMC8291647; doi:10.1371/journal.pone.0233242)
Supplement: S2 Table — (DOCX) [file pone.0233242.s002.docx]

S2 Table. Accuracy of GBM and Neural network models using k-fold cross validation (k = 10)

|  | |  |  |  | GBM | | | | | | | | Neural Network | | | | | | | | |
| --- | --- | --- | --- | --- | --- | --- | --- | --- | --- | --- | --- | --- | --- | --- | --- | --- | --- | --- | --- | --- | --- |
| Set | Dependent  Variables | | Independent variables | Laboratory method  for calibration | Sand | Silt | Clay | C | Sand | Silt | Clay | C | Sand | Silt | Clay | C | Sand | Silt | Clay | C | N |
|  | |  |  |  | Adjusted R^2^ | | | | RMSE | | | | Adjusted R^2^ | | | | RMSE | | | |  |
| Set7 | | Ilr | All features,  NIR-2X | 2-point 2-h sedimentation | 0.98 | 0.93 | 0.97 | 0.52 | 4.76 | 4.69 | 3.41 | 0.53 | 0.76 | 0.41 | 0.67 | 0.00 | 15.78 | 13.50 | 10.46 | 0.77 | 156 |
|  |  |  | No feature,  NIR-2X |  | 0.94 | 0.80 | 0.94 | 0.61 | 8.13 | 7.90 | 4.33 | 0.48 | 0.79 | 0.53 | 0.64 | 0.01 | 14.62 | 12.03 | 10.94 | 0.74 |  |
|  |  |  | Carbon, NIR-2X |  | 0.95 | 0.90 | 0.95 | 0.63 | 6.98 | 5.66 | 4.03 | 0.47 | 0.81 | 0.63 | 0.58 | 0.01 | 14.02 | 10.60 | 11.85 | 0.74 |  |
|  |  |  | Bulk density,  NIR-2X |  | 0.95 | 0.90 | 0.94 | 0.77 | 7.13 | 5.42 | 4.66 | 0.37 | 0.67 | 0.66 | 0.38 | 0.00 | 18.28 | 10.22 | 14.43 | 0.77 |  |
|  |  |  | pH, NIR-2X |  | 0.91 | 0.74 | 0.95 | 0.48 | 9.86 | 9.03 | 4.26 | 0.56 | 0.65 | 0.64 | 0.38 | 0.00 | 18.86 | 10.47 | 14.40 | 0.75 |  |
|  |  |  | Color, NIR-2X |  | 0.96 | 0.92 | 0.97 | 0.55 | 6.27 | 4.89 | 3.23 | 0.52 | 0.83 | 0.39 | 0.67 | 0.00 | 13.04 | 13.65 | 10.56 | 0.77 |  |
|  |  |  | Oxalate, NIR-2X |  | 0.94 | 0.87 | 0.93 | 0.46 | 7.92 | 6.34 | 4.93 | 0.57 | 0.77 | 0.60 | 0.57 | 0.01 | 15.18 | 11.13 | 12.04 | 0.74 |  |
|  |  |  | Mehlich3, NIR-2X |  | 0.97 | 0.84 | 0.95 | 0.43 | 5.65 | 7.05 | 4.00 | 0.58 | 0.90 | 0.69 | 0.75 | 0.00 | 10.00 | 9.80 | 9.06 | 0.76 |  |

C: Carbon; PT: Pre-treatments (no peroxide or peroxide); MIR: MIR scores; RMSE: root mean square error; N: Sample size
