# Supplementary material for: Determining soil particle-size distribution from infrared spectra using machine learning predictions: Methodology and modeling
Source: PLoS One. 2021 Jul 20;16(7):e0233242. doi: 10.1371/journal.pone.0233242 (PMC8291647; doi:10.1371/journal.pone.0233242)
Supplement: S1 File — (PDF) [file pone.0233242.s003.pdf]

Soil texture

Elizabeth and Serge-Etienne Parent

10/12/2019

Objectives

- The objective of this code is to use spectral print from air-dried 2mm sieved soil sample and predict sand, silt and clay percentages, using preprocessing and machin learning intermediates.

Latex installation

```
#library(tinytex)
#tinytex::pdflatex('stats4')
#install.packages('tinytex')
#tinytex::install_tinytex()
#tinytex:::is_tinytex()
```

Initiate session

```
session_seed = 14966 # random.org
set.seed(session_seed)
```

Load packages

Several packages are needed through the notebook. The **tidyverse** package is a meta package which loads generic tools for R. The **reshape2** package is used for flexible table restructure and **stringi** is a character string or text processing. The **signal** package includes filtering functions. The **caret** is a metapackage used for machine learning. The **compositions** package is used to handle compositional data (texture and oxalate compositions). The **mvoutlier** package detects multivariate outliers. The **soiltexture** package categorizes texture data into international code standards and **aqp** transforms Munsell color to red-blue-green.The **patchwork** and **broom** packages are used for graphs and data frames visualisation. Sources cited are used for ilr calulations.

```
library("tidyverse")
```

```
## Registered S3 methods overwritten by 'ggplot2':
##   method         from
##   [.quosures     rlang
##   c.quosures     rlang
##   print.quosures rlang
```

```
## -- Attaching packages ------------------------------------------------------------------------------
```

```
## v ggplot2 3.1.1     v purrr   0.3.2
## v tibble  2.1.1     v dplyr   0.8.1
## v tidyr   0.8.3     v stringr 1.4.0
## v readr   1.3.1     v forcats 0.4.0

## -- Conflicts ---------------------------------------------------------------------------------
## x dplyr::filter() masks stats::filter()
## x dplyr::lag()    masks stats::lag()
```

```r
theme_set(theme_bw())
library("reshape2")
```

```
##
## Attaching package: 'reshape2'

## The following object is masked from 'package:tidyr':
##
##     smiths
```

```r
library("stringi")
library("signal")
```

```
##
## Attaching package: 'signal'

## The following object is masked from 'package:dplyr':
##
##     filter

## The following objects are masked from 'package:stats':
##
##     filter, poly
```

```r
library("mvoutlier")
```

```
## Loading required package: sgeostat

## Registered S3 method overwritten by 'GGally':
##   method from
##   +.gg   ggplot2

## sROC 0.1-2 loaded
```

```r
library("caret")
```

```
## Loading required package: lattice

##
## Attaching package: 'caret'
```

```
## The following object is masked from 'package:purrr':
##
##     lift
```

```r
library("compositions")
```

```
## Loading required package: tensorA

##
## Attaching package: 'tensorA'

## The following object is masked from 'package:base':
##
##     norm

## Loading required package: robustbase

## Loading required package: energy

## Loading required package: bayesm

## Welcome to compositions, a package for compositional data analysis.
## Find an intro with "? compositions"

##
## Attaching package: 'compositions'

## The following object is masked from 'package:caret':
##
##     R2

## The following objects are masked from 'package:stats':
##
##     cor, cov, dist, var

## The following objects are masked from 'package:base':
##
##     %*%, scale, scale.default
```

```r
library("soiltexture")
library("aqp") # to use munsell2rgb
```

```
## This is aqp 1.17

##
## Attaching package: 'aqp'

## The following objects are masked from 'package:dplyr':
##
##     slice, union
```

```
## The following object is masked from 'package:base':
##
##     union
```

```r
library("patchwork")
library("broom")
library("ggExtra")
```

```
## Warning: package 'ggExtra' was built under R version 3.6.1
```

```r
library("outliers")

# Custom functions
source("lib/ilrNA.R")
source("lib/ilrDefinition.R")
```

Load data

Twelve csv files are loaded.

- `noPeroxide.csv` contains density of a hexametaphosphate-soil sample dispersion mixture (g/L) measured by a hydrometer after 0.75, 5, 120, 420, 1440 min. Particle size distribution was analysed using the sedimentation method (Sheldrick and Wang, 1993; Day, 1965) without pretreatments as peroxide addition. Texture was performed on soil library at Department of Soils and Agrifood Engineering, Université Laval, Québec, Canada. Soils were air dried and 2mm sieved.
- `peroxide.csv` contains density of the dispersion mixture (g/L) measured by a hydrometer after 0.75, 5, 120, 420, 1440 min. Particle size distribution was analysed using the sedimentation method (Sheldrick and Wang, 1993; Day, 1965) with 30% hydrogen peroxide pretreatment. Texture was performed on soil library at Department of Soils and Agrifood Engineering, Université Laval, Québec, Canada. Soils were air dried and 2mm sieved.
- `peroxide_estim .csv` contains density of the dispersion mixture (g/L) measured by a hydrometer after 0.67 and 120 min. Particle size distribution was analysed using the sedimentation method (Kalra and Maynard, 1992) with 30% hydrogen peroxide pretreatment applicated on over 5% carbon soil samples. Texture was performed on soils at Agriculture and Agri-Food Canada, Québec, Canada. Soils were air dried and 2mm sieved.
- `fractSand.csv` contains the sand fraction, firstly retained using a 0.053 mm sieve after hydrometer sedimentation experimentation. Samples were then dried overnight at 105°C. They were passed through five consecutive sieves of 1, 0.5, 0.25, 0.10 and 0.05 mm diameters and shaked for 10 min. Sand fractions method was performed on soil library at Department of Soils and Agrifood Engineering, Université Laval, Québec, Canada. Soils were air dried and 2mm sieved.
- `laser.csv` contains particule sizes quantification determined using a MASTERSIZER 2000 Laser Particle Size Analyzer (MALVERN Instruments, Worcestershire, UK) and 2 min sonification (evaluation will be shown for 0 to 8 min). Texture was performed on soil library at Department of Soils and Agrifood Engineering, Université Laval, Québec, Canada. Soils were air dried and 2mm sieved.
- `text_NIR.csv` contains Fourier transform near infrared spectra of various soil samples resulted from spinning cup reflectance using an integrated sphere diffuse reflection InGaAs detector Nicolet Antaris II technology (4000 - 9090 cm-1, every 2 cm-1, gain of 2X) (Thermo Electron Corp., Ann Arbor, Mich.). Experimentation was performed on soil library at Department of Soils and Agrifood Engineering and Agriculture and Agri-Food Canada, Québec, Canada, Université Laval, Québec, Canada. Soils were air dried and 2mm sieved.
- `text_NIR.csv` contains Fourier transform near infrared spectra of various soil samples resulted from spinning cup reflectance using an integrated sphere diffuse reflection InGaAs detector Nicolet Antaris II

technology (4000 - 9090 cm-1, every 2 cm-1, gain of 4X) (Thermo Electron Corp., Ann Arbor, Mich.). Experimentation was performed on soil library at Department of Soils and Agrifood Engineering and Agriculture and Agri-Food Canada, Québec, Canada, Université Laval, Québec, Canada. Soils were air dried and 2mm sieved.

- `text_MIR.csv` contains mid infrared spectra of different soil samples using a transmission DTGS detector Varian 1000 FT-IR Scimitar series spectrometer (Varian inc., Palo Alto, CA). 2g of soil were grounded through a 200 mesh (Le Guillou et al., 2015). Then, 0.002g of sample was mixed with a grounded 0.2g of KBr and pressed at 15000lbs (517 MPa) for three minutes using a manual hydraulic press (Carver, Model 4350.L, Carver Inc., Wabash, Indiana). Pellets were dried at 110C for 2-3 hours. MIR scanned were registered in the range of 400 to 4000 cm-1 with a resolution of 4 cm-1 (2500-25 000 nm, every 0.6nm) (Madejova and Konadel, 2001). Two pellets and two scans with 45 degre pellet rotation were made to optimize precision totaling 20 scans. Scans were corrected for ambiant air as background. Experimentation was performed on soil library at Department of Soils and Agrifood Engineering and Agriculture and Agri-Food Canada, Québec, Canada, Université Laval, Québec, Canada. Soils were air dried and 2mm sieved.
- `Cdensity.csv` contains soil density determined by weight with a 3cc coppermade spoon. Soil samples were air dried and 2 mm sieved. The file also contains total soil carbon, nitrogen and sulfur percentages using CNS Leco analyzer (Leco Corporation, St.Joseph, Michigan). Finally, various carbon analysis were also added: Walkley-Black method, 'lost in ignition' method, active carbon (Gugino et al., 2009; Weil et al., 2003). Experimentations were performed on soil library at Department of Soils and Agrifood Engineering and Agriculture and Agri-Food Canada, Québec, Canada, Université Laval, Québec, Canada. Soils were air dried and 2mm sieved.
- `color_oxalate.csv` contains iron, calcium, magnesium and manganese oxides and silicate. They were determined according to McKeague and Day (1966), McKeague (1978) and Ross and Wang (1993) methods. Soil color of dried soil samples was determined using a Munsell chart (Hue, value, chroma). Experimentations were performed on soil library at Department of Soils and Agrifood Engineering. Soils were air dried and 2mm sieved. `-pH_M3.csv` contains pH determination using a 0.01M CaCl2 solution and M3 extracts.

```r
no_peroxide <- read.table(file = "data/NoPeroxideWeight.csv", # no peroxide weight
                          sep=";", dec=".", header=TRUE)
peroxide <- read.table(file = "data/PeroxideWeight.csv", # peroxide weight
                       sep=";", dec=".", header=TRUE)
peroxide_estim <- read.table(file = "data/agric_canada.csv", sep=";", dec=",", header=TRUE)
multi_point <- read.table(file = "data/SLA_bouy_calc.csv", sep=";", dec=",", header=TRUE)
text_fract_sand <- read.table(file = "data/fract_unique.csv",
                              sep=";", dec=".", header=TRUE)
laser <- read.csv(file = "data/Laser.csv",
                  sep=";", dec=".", header=TRUE)
text_NIR <- read.table(file = "data/totalsoils.csv",
                       sep=";", dec=",", header=TRUE)
text_NIR_4X <- read.table(file = "data/totalsoils4X.csv",
                          sep=";", dec=",", header=TRUE)
text_MIR <- read.table(file = "data/MIR2.csv",
                       sep=";", dec=".", header=TRUE)
c_density <- read.table(file = "data/C-density.csv", sep=";", dec=".", header=TRUE)
color_oxalate <- read.table(file = "data/colour-oxalate.csv", sep=";", dec=".", header=TRUE)
ph_m3 <- read.csv(file = "data/Inventaire1.csv", sep=";", dec=".", header=TRUE)
```

The data sets are connected by the sample identifier, which is the IDEN column. Objects were homogenized as numeric or character class.

```
c_density$IDEN <- as.character(c_density$IDEN)
color_oxalate$IDEN <- as.character(color_oxalate$IDEN)
peroxide_estim$IDEN <- as.character(peroxide_estim$IDEN)
text_fract_sand$IDEN <- as.character(text_fract_sand$IDEN)
multi_point$IDEN <- as.character(multi_point$IDEN)
ph_m3$IDEN <- as.character(ph_m3$IDEN)
laser$IDEN <- as.character(laser$IDEN)
```

Organization of data

Organisation of sedimentation data

We add a 'Treatment' column to specify the pretreatment type (with or without peroxide).We add a `Method_treatment` column to specify Method combined with pretreatment type (with or without peroxide) and a `IDEN_Method_treatment` column to specify a unique identification.

```
peroxide <- peroxide %>%
dplyr::  mutate(., Treatment = 'Peroxide', Method =  'Bouyoucos 2-point 2-h', Method_treatment = paste(
no_peroxide <- no_peroxide %>%
dplyr::  mutate(., Treatment = 'No peroxide', Method =  'Bouyoucos 2-point 2-h', Method_treatment = past
```

Both `peroxide` and `no_peroxide` tables are combined by rows in a table named `time`, whose columns are renamed. We reshape the `time` data frame for time period and density results (renamed as 'Time' and 'Density'), while keeping 'IDEN' and 'Method_treatment'.Five tuples `IDEN-Method_treatment` are repetitions. `Unique` function to avoid repetitions is used for each sample identified by ÌDEN_Method_treatment'.

```
times <- rbind(peroxide, no_peroxide)
colnames(times) <- c("IDEN","0.75", "5", "120", "420", "1440", "Treatment", "Method", "Method_treatment
times <- times %>%
  dplyr::select(., c("IDEN_Method_treatment", "IDEN", "Method", "Treatment", "0.75", "5", "120", "420",
  distinct(IDEN_Method_treatment, .keep_all = TRUE)
times <- melt(times, id.vars = c('IDEN_Method_treatment', 'IDEN', 'Method', 'Treatment')) #reshape
times$variable <- as.numeric(as.character(times$variable))
names(times) <- c('IDEN_Method_treatment', 'IDEN', 'Method', 'Treatment', 'Time', 'Density')
```

`peroxide_estim` is a table where 2-points calcuted mesurements are stored. The 2-points 2-hours Boyoucos method is considered as an estimated texture method, compared to a five points mesurements (`noPeroxide` and `peroxide`). For consistency, the number `6` in the `peroxide_estim` table becomes `No peroxide` and the number `7` becomes `Peroxide`.

```
# Texture estimated in two points line
peroxide_estim$Treatment <- ifelse(peroxide_estim$Method == 6, 'No peroxide', 'Peroxide')
peroxide_estim <- peroxide_estim %>%
  dplyr::mutate(Method = c("Bouyoucos 2-points 2-h"), Method_treatment = paste(Method, " ", Treatment),

agric_carbon <- peroxide_estim %>%
  rename(SAND = S, SILT = Si, CLAY = Clay, C_perc = Carbon)
```

In `multi_point`, i.e. the table where multipoint 7-hour Boyoucos data are stored, we add a `Method_treatment` column to specify pretreatment type (with or without peroxide). We homogenize titles as SAND, SILT

and CLAY percentages. `Unique` function to avoid repetitions is used for each sample identified by ÌDEN_Method_treatment‘.

```
multi_point$Treatment <- ifelse(multi_point$Method == 2, "No peroxide", "Peroxide")
multi_point$Method <- c("Bouyoucos multi-point 7-h")
multi_point_7h <- multi_point %>%
  select(IDEN, Method, Treatment, Sable, Limon, Argile) %>%
  dplyr::mutate(., IDEN_Method_treatment = paste(IDEN, " ", Method, " ", Treatment), Method_treatment =
  dplyr::select(., IDEN_Method_treatment, IDEN, Method, Treatment, Method_treatment, SAND, SILT, CLAY)
  group_by(IDEN_Method_treatment, Method_treatment) %>%
  na.omit() %>%
  distinct(IDEN_Method_treatment, .keep_all = TRUE)

multi_carbon <- multi_point_7h %>%
  left_join(., c_density[c('IDEN', 'C_perc')], by = c("IDEN")) %>%
  as_tibble(.)
```

In `fract_sand`, we add columns corresponding to unique identification, metholodogy used and treatment are also made. We remove NAs and negative results from `fractSand`.

```
fract_sand <- text_fract_sand %>%
  mutate(Method = c("Sieving"), Treatment = c("No peroxide"), Method_treatment = paste(Method, " ", Trea
  na.omit() %>%
  group_by(IDEN_Method_treatment) %>%
   distinct(IDEN_Method_treatment, .keep_all = TRUE)
```

We aim to compute slope and intercept of the log-log density-time curve. Those parameters are used to compute the PSD. Later on, the spectra will be used to predict these parameters. Using **unique** function, we keep no sample repetition.

```
times$test <- paste(times$IDEN, "Bouyoucos", times$Treatment)
tests <- unique(times$test)
time_coefficients <- data.frame(IDEN_Method_treatment = rep(NA, length(tests)),
                                IDEN = rep(NA, length(tests)),
                                Method = rep(NA, length(tests)),
                                Treatment = rep(NA, length(tests)),
                                Intercept = rep(NA, length(tests)),
                                Slope = rep(NA, length(tests)))
```

We create a dataframe for slope and intercept results.

```
for (i in 1:length(tests)) {
  data_lm <- times[times$test == tests[i], ] # new dataframe empty with time$test lenght
  data_lm <- data_lm[!is.na(data_lm$Density), ] # just in case 0s are found in density in df 'time'
  data_lm <- data_lm[data_lm$Density != 0, ] # just in case 0s are found in density
  time_coefficients[i, 1] <- data_lm[1, 1] # put direct information from first column 'IDEN_Method_trea
  time_coefficients[i, 2] <- data_lm[1, 2] # put direct information from second column 'IDEN'
  time_coefficients[i, 3] <- data_lm[1, 3] # put direct information from second column 'Method'
  time_coefficients[i, 4] <- data_lm[1, 4] # put direct information from second column 'Treatment'
  if (nrow(data_lm) == 0) { # if there is '0' in 'Time' and 'Density' columns, put 'NA'
    time_coefficients[i, 5] <- NA
    time_coefficients[i, 6] <- NA
```

```
  } else {
    if (any(is.na(data_lm[, c('Time', 'Density')]))) { # if there is no data in 'Time' and 'Density' co
      time_coefficients[i, 5] <- NA
      time_coefficients[i, 6] <- NA
    } else {
      mod_lm <- lm(log(Density) ~ log(Time), data = data_lm) # if there is data > 0, calculate ln on 'D
      time_coefficients[i, 5] <- coef(mod_lm)[1] # Insert coefficient, here intercept from linear model
      time_coefficients[i, 6] <- coef(mod_lm)[2] # Insert coefficient, here slope from linear model
    }
  }
}
```

For example, a fitted density-time model looks like this.

```
IDEN_i <- 100
data_lm <- times[times$IDEN == IDEN_i, ]
plot(data_lm$Time, data_lm$Density, ylim = c(0.1, max(data_lm$Density)))
t <- 10^seq(0, 3.2, length=50)
d <-  exp(time_coefficients$Intercept[time_coefficients$IDEN == IDEN_i]
          + log(t) * time_coefficients$Slope[time_coefficients$IDEN == IDEN_i])
```

```
## Warning in log(t) * time_coefficients$Slope[time_coefficients$IDEN ==
## IDEN_i]: la taille d'un objet plus long n'est pas multiple de la taille
## d'un objet plus court
```

```
## Warning in time_coefficients$Intercept[time_coefficients$IDEN == IDEN_i]
## + : la taille d'un objet plus long n'est pas multiple de la taille d'un
## objet plus court
```

```
lines(x=t, y=d, pch=16, cex=0.2)
```

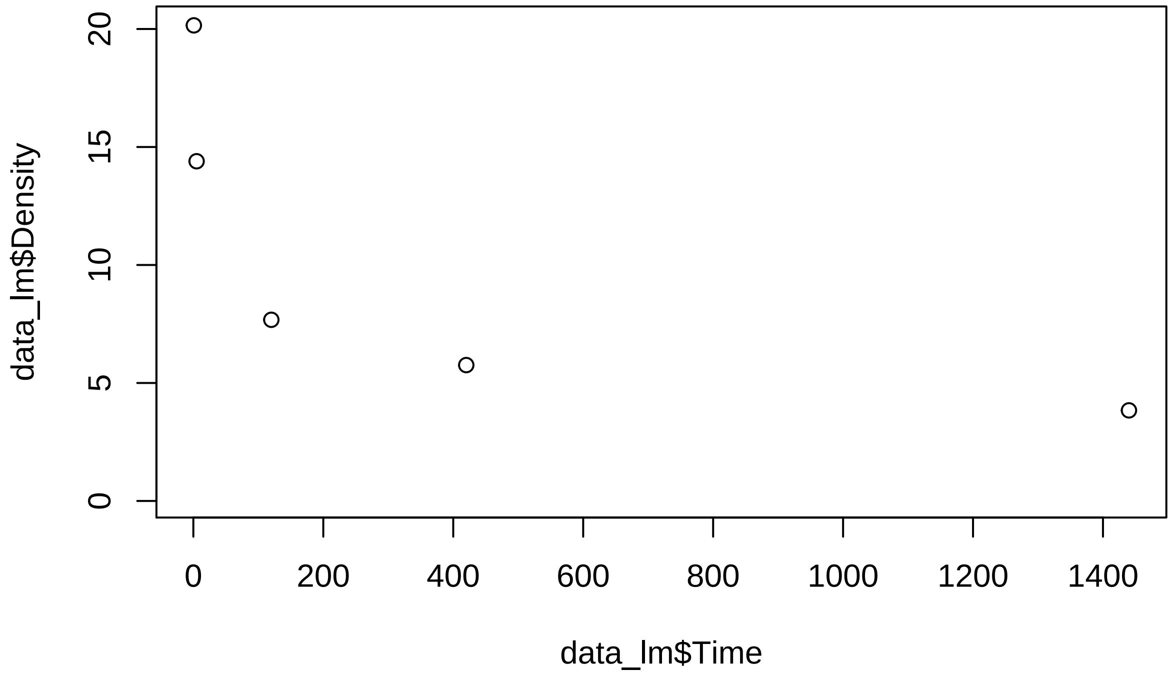

The previously computed slopes and intercepts are used to calculate sand, silt and clay percentages. Percentage of combined silt and clay is calculated at 40 seconds from equation curve. Clay percentage is calculated at 2 hours. Silt percentage is resulted from substraction of silt and clay combination and clay percentage. Sand results from rest part of 100% total. We keep only rows without NA and negative results. We add carbon percentage in `time_carbon`.

```
silt_clay <- exp(time_coefficients$Intercept + log(40/60) * time_coefficients$Slope)
silt_clay <- ifelse(silt_clay > 100, 99, silt_clay)
time_coefficients$CLAY <- exp(time_coefficients$Intercept + log(120) * time_coefficients$Slope)
time_coefficients$SILT <- silt_clay - time_coefficients$CLAY
time_coefficients$SAND <- 100 - silt_clay
time_coefficients <- time_coefficients %>%
  dplyr::filter(., CLAY >= 0 & SILT >= 0 & SAND >= 0) %>%
  as.data.frame()
time_coefficients$IDEN <- as.character(time_coefficients$IDEN)

time_carbon <- time_coefficients %>%
  left_join(., c_density[c('IDEN', 'C_perc')], by = c("IDEN")) %>%
  mutate(., Method_treatment = paste(Method, Treatment)) %>%
  as_tibble(.)
```

We visualize partition of clay with silt and clay combination. We keep only positive results with function `which`. Classes are made with `TT.plot` from **soiltexture** package to visualize on triangle textural figure.

```
tiff(filename = "images/ternary-texture.tiff", width = 1800, height = 1800, units = "px", pointsize = 24
TT.plot(class.sys = "USDA.TT",
        main = NA,
        tri.data = data.frame(time_coefficients[, c("CLAY", "SILT", "SAND")]),
        col = rgb(0, 0, 0, 0.25),
        pch=16, cex=0.8, frame.bg.col = "white")
dev.off()
```

```
## pdf
##   2
```

Organisation of laser data

Thanks to Jonathan Lafond (Université Laval) for his help here!

We rename first row for complete title. We calculate SAND, SILT and CLAY from the `laser` data set. We select columns into a `tbl` data frame. Average data from `laser_select` are isolated from repetitions. SAND, SILT and CLAY percentages were obtained using `rowSums` function. Nas and negative data were removed.

```
laser_select <- cbind(laser[, 1:3],laser[, 29:31],laser[, 33:133])
laser_select <- laser_select[-c(1), -c(3)]

laser_perc <- laser_select %>%
  mutate(Repetition = rep(seq(4), 364)) %>%  # For each sample, 4 lines including average
  dplyr::filter(Repetition == 4) %>% # We just keep the average, corresponding to no4.
  mutate(CLAY = rowSums(.[, 7:44]), SILT = rowSums(.[, 45:67]), SAND = rowSums(.[, 68:105]), IDEN_Metho
  as.data.frame(.) %>%
  dplyr::select(., IDEN_Method_treatment, IDEN, Method, Treatment, Method_treatment, Sonification_min, S
  distinct(IDEN_Method_treatment, .keep_all = TRUE) %>%
  na.omit(.)

laser_carbon <- laser_perc %>%
  left_join(., c_density[c('IDEN', 'C_perc')], by = c("IDEN")) %>%
  dplyr::filter(., Sonification_min  %in% c(0, 2)) # Only 2min sonicated and without sonication samples
```

We compare laser data with Bouyoucos for errors investigation.

```
compare <- time_coefficients[,c("IDEN", "SAND", "SILT", "CLAY")] %>%
  left_join(., laser_perc[, c("IDEN", "SAND", "SILT", "CLAY")], by= "IDEN") %>%
  mutate(factor_sand = SAND.x-SAND.y, factor_silt = SILT.x-SILT.y, factor_clay = CLAY.x-CLAY.y)
compare <- compare[!(apply(compare, 1, function(x) any(is.na(x)))),]
compare <- compare %>%
  left_join(., c_density[, c("IDEN", "C_perc")], by = "IDEN") %>%
  rename(SAND_bouy = SAND.x, SILT_bouy = SILT.x, CLAY_bouy = CLAY.x, SAND_laser = SAND.y, SILT_laser = S
#summary(compare)
```

Preprocessing of infrared spectroscopy

We create a function `infrared`for spectra processing. Procedure: - We remove negative from NIR data. - We compute the mean of NIR and MIR data - We apply binning on NIR spectra. Binning is a mean of 10

points spectra to light file. - We test noise removal techniques. - We used first derivatives, scaling and PCA to remove some noise.

```
infrared <- function(data) {

  # Normalize spectra
  ## Remove negative data
  data<- data[!(apply(data, 1, function(x) any(is.na(x)))),]
  data<- data[!(apply(data, 1, function(x) any(x<0))),]
  data<- data%>%
    group_by(IDEN) %>%
    summarise_all(funs(mean))

  ## Remove X from titles
  wavelength <- colnames(data)[-1]
  wavelength <- stringi::stri_sub(wavelength, 2)
  wavelength <- as.numeric(wavelength)
  colnames(data)[2:ncol(data)] <- wavelength

  ## Binning
  bin_each <- 10
  temp_bin <- data.frame(t(data[, -1]))
  temp_bin$group <- rep(1:ceiling(length(wavelength)/bin_each),
                        each = bin_each)[1:length(wavelength)]
  IR_bin <- temp_bin %>%
    group_by(group) %>%
    summarise_all(funs(mean)) %>%
    t(.)
  IR_bin <- IR_bin[-1, ]

  IR_wl_bin <- data.frame(wl = wavelength, group = temp_bin$group) %>%
    group_by(group) %>%
    summarise_all(funs(mean))

  colnames(IR_bin) <- IR_wl_bin$wl
  IR_deriv <- t(apply(IR_bin, 1, signal::sgolayfilt, m = 1))
  IR_sc <- t(apply(IR_deriv, 1, scale))

  # PCA
  IR_svd = svd(IR_sc[, -c(622:623)])
  IR_scores = IR_svd$u %*% diag(IR_svd$d)
  plot(IR_scores[, 1], IR_scores[, 2])

  IR_scores <- data.frame(IDEN = data$IDEN, IR_scores)

  write.table(IR_scores, file = "C:/Users/eliza/Documents/Jusque 2019/Spectres_sol/output/IR_scores.csv
  return(IR_scores)
}
```

NIR spectra with 2X gain

```r
if (!file.exists("output/NIR_scores.csv")) {
  NIR_scores <- infrared(text_NIR)
    write.table(NIR_scores, file = "C:/Users/eliza/Documents/Jusque 2019/Spectres_sol/output/NIR_scores
} else {
  NIR_scores <- read.csv("output/NIR_scores.csv", sep = ',', dec = '.')
  NIR_scores$IDEN <- as.character(NIR_scores$IDEN)
}
```

NIR spectra with 4X gain

```r
if (!file.exists("output/NIR4X_scores.csv")) {
  NIR4X_scores <- infrared(text_NIR_4X)
    write.table(NIR4X_scores, file = "C:/Users/eliza/Documents/Jusque 2019/Spectres_sol/output/NIR4X_sco
} else {
  NIR4X_scores <- read.csv("output/NIR4X_scores.csv", sep = ',', dec = '.')
  NIR4X_scores$IDEN <- as.character(NIR4X_scores$IDEN)
}
```

MIR spectra

```r
if (!file.exists("output/MIR_scores.csv")) {
  MIR_scores <- infrared(text_MIR)
  write.table(MIR_scores, file = "C:/Users/eliza/Documents/Jusque 2019/Spectres_sol/output/MIR_scores.c
} else {
  MIR_scores <- read.csv("output/MIR_scores.csv", sep = ',', dec = '.')
  MIR_scores$IDEN <- as.character(MIR_scores$IDEN)
}
```

Other features

In addition to spectra, some features are added to be used as predictor later on for machine learning. These features are color, carbon, oxalate extracts, density parameters.

Colors

Colors are converted in RGB using the `munsell2rgb` function from **aqp** package.

```r
# color_oxalate
color_oxalate <- drop_na(color_oxalate)

## Colors
color_oxalate$munsell <- ifelse(color_oxalate$Hue == "", NA, paste0(color_oxalate$Hue, ' ', color_oxala
color_rgb <- munsell2rgb(the_hue = color_oxalate$Hue,
                         the_value = color_oxalate$Value,
                         the_chroma = color_oxalate$Chroma,
                         return_triplets = TRUE)
```

```
## Notice: converting hue to character
```

```
color_oxalate <- cbind(color_oxalate, color_rgb)
color_rgb$IDEN <- as.character(color_oxalate$IDEN)
```

Function `new_ilr`is created for ilr transformation.

```
new_ilr <- function (data, comp_ilr, sbp_ilr) {
bal_ilr <- ilr(comp_ilr, V = gsi.buildilrBase(t(sbp_ilr)))
bal_ilr <- ilrNA(comp = comp_ilr, sbp = sbp_ilr, bal = bal_ilr)
bal_ilr <- data.frame(bal_ilr)
ilr_def <- ilrDefinition(sbp = sbp_ilr, side="-+")
colnames(bal_ilr) <- ilr_def
bal_ilr$IDEN <- as.character(data$IDEN)
print(bal_ilr)
return(bal_ilr)
}
```

Oxalate extracts

Ilr transformations are applied on oxalate extracts.

```
## Oxalate
sbp_oxalate <- matrix(c(-1, 1,-1,-1,-1, 1,
                        -1, 0,-1,-1, 1, 0,
                        -1, 0,-1, 1, 0, 0,
                        -1, 0, 1, 0, 0, 0,
                         0,-1, 0, 0, 0, 1),
                      byrow = TRUE, ncol=6)
colnames(sbp_oxalate) <- colnames(color_oxalate)[6:11]

comp_oxalate <- acomp(color_oxalate[, 6:11])
bal_oxalate <- new_ilr(color_oxalate, comp_oxalate, sbp_oxalate)
bal_oxalate <- bal_oxalate %>%
dplyr::rename(ilr1_ox = `[Pox_mgPkg,Mnox_mgPkg,Feox_mgPkg,Alox_mgPkg | Siox_mgPkg,Caox_mgPkg]`, ilr2_ox
```

Mehlich-3 extracts

Ilr balances are calculated for M-3 extracts.

```
# M3
m3 <- ph_m3 %>%
  dplyr::select(IDEN, P_M3_mgPkg, K_M3_mgPkg, Ca_M3_mgPkg, Mg_M3_mgPkg, Al_M3_mgPkg, Fe_M3_mgPkg, Mn_M3
m3 <- drop_na(m3)

sbp_m3 <- matrix(c(-1,-1,-1,-1,-1,-1,-1, 1, 1,
                    0, 0, 0, 0, 0, 0, 0,-1, 1,
                   -1,-1,-1,-1, 1, 1, 1, 0, 0,
                    0, 0, 0, 0,-1,-1, 1, 0, 0,
                    0, 0, 0, 0,-1, 1, 0, 0, 0,
                   -1, 1, 1, 1, 0, 0, 0, 0, 0,
```

```
                   0,-1, 1, 1, 0, 0, 0, 0, 0,
                   0, 0,-1, 1, 0, 0, 0, 0, 0),
                 byrow = TRUE, ncol=9)
colnames(sbp_m3) <- colnames(m3)[2:10]

comp_m3 <- acomp(m3[, 2:10])
bal_m3 <- new_ilr(m3, comp_m3, sbp_m3)

bal_m3 <- bal_m3 %>%
  dplyr::rename(., ilr1_M3 = `[Mn_M3_mgPkg,Fe_M3_mgPkg,Al_M3_mgPkg,Mg_M3_mgPkg,Ca_M3_mgPkg,K_M3_mgPkg,P_
```

All data combinations

We combined all methods in a data frame. We verify that each sample has a 100% sand, silt, clay total. We remove Nas from data frame.

```
# Combined methods in data frame
three_methods_q <- time_carbon %>%
  full_join(agric_carbon, by = c("IDEN_Method_treatment", "IDEN", "Method", "Treatment", "SAND", "SILT"
  full_join(multi_carbon, by = c("IDEN_Method_treatment", "IDEN", "Method", "Treatment", "SAND", "SILT"
  full_join(laser_carbon, by = c("IDEN_Method_treatment", "IDEN", "Method", "Treatment", "SAND", "SILT"
  as.tibble(.)
```

```
## Warning: `as.tibble()` is deprecated, use `as_tibble()` (but mind the new semantics).
## This warning is displayed once per session.
```

```
print("Proportion of NAs in the three_methods_q table")
```

```
## [1] "Proportion of NAs in the three_methods_q table"
```

```
print(sum(is.na(three_methods_q$C_perc)) / nrow(three_methods_q))
```

```
## [1] 0.07201349
```

```
# 100% sand, silt, clay total
three_methods_q1 <- three_methods_q %>%
  dplyr::mutate(SAND = 100 - SILT - CLAY) %>%
  dplyr::select(IDEN_Method_treatment, IDEN, Method, Treatment, Method_treatment, SAND, SILT, CLAY, C_pe

#Nas removal
three_methods_q1 <- three_methods_q1[complete.cases(three_methods_q1),]
```

Ilr transformation for sand, silt and clay percentages

Carbon, sand, silt and clay percentages are coverted into ilr balances. As, carbon represents the organic matter, it is considered as a part of the soil texture. We have discarded it from publication results because subject is focussing on sand, silt and clay percentages.
We join data frames together.

```r
sbp_texture_q1 <- matrix(c( 1, 1, 1,-1,
                           -1, 1, 1, 0,
                            0,-1, 1, 0),
                        byrow=TRUE, ncol=4)
three_methods_q1 <- three_methods_q1[-3733,]
parts_texture_q1 <- three_methods_q1[, c('CLAY', 'SILT', 'SAND', 'C_perc')]
parts_texture_q1_sc <- parts_texture_q1
parts_texture_q1_sc$total <- rowSums(parts_texture_q1_sc)
parts_texture_q1_sc_closed <- parts_texture_q1_sc %>%
dplyr::mutate(SAND = SAND/total*100,
SILT = SILT/total*100,
CLAY = CLAY/total*100,
C_perc = C_perc/total*100)

parts_texture_q1_sc_closed <- parts_texture_q1_sc_closed %>% dplyr::select(-total)
parts_texture_q1_sc_closed <- normalize(parts_texture_q1_sc_closed)
```

The mineral part and C are not scaled to the same constant sum. C is scaled on [C, mineral part]. So clay, silt and sand are part of the [1-C] subcomposition.

```r
#parts_texture_q1_sc_mineral <- as.matrix(parts_texture_q1_sc[, c('CLAY', 'SILT', 'SAND')])
#parts_texture_q1_sc_closed <- cbind(parts_texture_q1_sc_mineral * (100-parts_texture_q1_sc[, c("C_perc
#                                    C_perc=parts_texture_q1_sc[, c("C_perc")])
```

We close the simplex to 1 with the acomp data class. Negative concentrations are considered BDL (beloww detection limit) data.

```r
which(apply(parts_texture_q1_sc_closed, 1, function(x) any(x < 0)))
parts_texture_q1_sc_closed[100, ]
```

We transform parts to ilr balances.

```r
comp_texture_q1 <- acomp(parts_texture_q1_sc_closed)
bal_texture_q1 <- new_ilr(three_methods_q1, comp_texture_q1, sbp_texture_q1)
bal_texture_q1$IDEN_Method_treatment <- as.character(three_methods_q1$IDEN_Method_treatment)
names(bal_texture_q1) <- c('[C | CLAY,SILT,SAND]', '[CLAY | SILT,SAND]', '[SILT | SAND]', "IDEN", "IDEN_

perc_methods <- three_methods_q1 %>%
  left_join(bal_texture_q1, by = c("IDEN_Method_treatment", "IDEN"))

perc_ilr <- perc_methods %>%
  dplyr::rename(ilr1 = `[C | CLAY,SILT,SAND]`, ilr2 =  `[CLAY | SILT,SAND]`, ilr3 = `[SILT | SAND]`)
```

Ilr balance and features are joined to data frame.

```r
perc_ilr_feat_2xscores <- perc_ilr %>%
  left_join(c_density[, c("IDEN", "Density_gPcm3")], by = "IDEN") %>%
  left_join(color_rgb, by = "IDEN") %>%
  left_join(bal_oxalate, by = "IDEN") %>%
  left_join(bal_m3, by = "IDEN") %>%
  left_join(NIR_scores, by = "IDEN")
```

We filter to isolate the 2min sonicated samples from other sonication.

```
perc_ilr_feat_2xscores_2min <- perc_ilr_feat_2xscores %>%
  dplyr::filter(Treatment != "0   min")
```

Questions

The purpose of this section if to answer the following questions.

1. Are there differences between methods in terms of sand, silt and clay? 1.2 Is there a difference between Bouyoucos no peroxided and peroxided? 1.3 Is there a difference between Bouyoucos with five points and with two points mesurements? 1.4 Is there a difference between Bouyoucos no peroxided and sieving? 1.5 Is there a difference between Bouyoucos peroxided and sieving? 1.6 Is there a difference between laser and sieving? 1.7 Is there a difference between Bouyoucos no peroxided and laser?

2. Can sand, silt and clay be predicted from NIR and MIR data? 2.1 Can machine learning predict [c | sS] and [s | S] balances from spectra PC and other features? 2.2 Can we predict sand, silt and clay percentages from NIR data? 2.3 Can we predict intercept and slope (Bouyoucos tests) from NIR data? 2.4 Can we predict probability density function (PDF) curve parameters generated from laser data using NIR data? 2.5 We finally resume results in tables and graphs.

Arrange data for Question 1 - Difference between methodologies

1.1 Test differences between methods

Visualization

We compare different ilr balance textures distributions. Textures colors are displayed by methodology.

```
gg_ilr <- ggplot(perc_methods, aes(x = `[CLAY | SILT,SAND]`, y = `[SILT | SAND]`)) +
  geom_point(aes(shape = Method, colour = Method), alpha = 0.5, size=2) +
  theme(legend.position="bottom")+
  scale_color_hue(l = 5)
gg_ilr_marg <- ggMarginal(gg_ilr, type="histogram", groupFill = TRUE)

ggsave("images/texture-balances-per-method.tiff", width=6, height=10)
```

```
## `stat_bin()` using `bins = 30`. Pick better value with `binwidth`.
```

Normalization of all ilr data

We calculate differences by ilr balances substraction between our original method (Bouyoucos No peroxide) and the other methods (Bouyoucos Peroxide, Laser and multi-points calculation for No peroxide and Peroxide). We combine all differences in a data frame for further investigations.

Bouyoucos No peroxide vs Bouyoucos Peroxide

```
bouy_2pts_np <- perc_ilr_feat_2xscores_2min %>%
dplyr::filter(., Treatment == "No peroxide" & Method == "Bouyoucos 2-point 2-h") %>%
dplyr::rename(ilr1_np = ilr1, ilr2_np = ilr2, ilr3_np = ilr3) %>%
dplyr::mutate(SAND_np = SAND, SILT_np = SILT, CLAY_np = CLAY)

bouy_2pts_p <- perc_ilr_feat_2xscores_2min %>%
dplyr::filter(., Treatment == "Peroxide" & Method == "Bouyoucos 2-point 2-h") %>%
dplyr::rename(ilr1_p = ilr1, ilr2_p = ilr2, ilr3_p = ilr3) %>%
dplyr::mutate(SAND_p = SAND, SILT_p = SILT, CLAY_p = CLAY)

bouy_2pts_np_p <- bouy_2pts_np[, c("IDEN", "ilr1_np", "ilr2_np", "ilr3_np", "SAND_np", "SILT_np", "CLAY_
left_join(bouy_2pts_p[, c("IDEN", "Method_treatment", "ilr1_p", "ilr2_p", "ilr3_p", "SAND_p", "SILT_p",
  na.omit() %>% unique(.) %>%
  mutate(ilr1_diff = ilr1_p - ilr1_np, ilr2_diff = ilr2_p - ilr2_np, ilr3_diff = ilr3_p - ilr3_np, SAND_
```

Bouyoucos No peroxide vs Laser 0 min sonificated

```
laser_0 <- perc_ilr_feat_2xscores %>%
  dplyr::filter(Treatment == "0   min") %>%
dplyr::rename(ilr1_0 = ilr1, ilr2_0 = ilr2, ilr3_0 = ilr3) %>%
dplyr::mutate(SAND_0 = SAND, SILT_0 = SILT, CLAY_0 = CLAY)

bouy_2pts_np_0 <- bouy_2pts_np[, c("IDEN", "ilr1_np", "ilr2_np", "ilr3_np", "SAND_np", "SILT_np", "CLAY_
left_join(laser_0[, c("IDEN", "Method_treatment", "ilr1_0", "ilr2_0", "ilr3_0", "SAND_0", "SILT_0", "CL
  na.omit() %>% unique(.) %>%
  mutate(ilr1_diff = ilr1_0 - ilr1_np, ilr2_diff = ilr2_0 - ilr2_np, ilr3_diff = ilr3_0 - ilr3_np, SAND_
```

Bouyoucos No peroxide vs Laser 2 min sonificated

```
laser_2 <- perc_ilr_feat_2xscores %>%
dplyr::filter(., Treatment == "2   min") %>%
dplyr::rename(ilr1_2 = ilr1, ilr2_2 = ilr2, ilr3_2 = ilr3) %>%
dplyr::mutate(SAND_2 = SAND, SILT_2 = SILT, CLAY_2 = CLAY)

bouy_2pts_np_2 <- bouy_2pts_np[, c("IDEN", "ilr1_np", "ilr2_np", "ilr3_np", "SAND_np", "SILT_np", "CLAY_
left_join(laser_2[, c("IDEN", "Method_treatment", "ilr1_2", "ilr2_2", "ilr3_2", "SAND_2", "SILT_2", "CL
  na.omit() %>% unique(.) %>%
  mutate(ilr1_diff = ilr1_2 - ilr1_np, ilr2_diff = ilr2_2 - ilr2_np, ilr3_diff = ilr3_2 - ilr3_np, SAND_
```

Bouyoucos 2-point 2-h vs multi points calculated

```
bouy_multi <- perc_ilr_feat_2xscores %>%
dplyr::filter(., Method == "Bouyoucos multi-point 7-h") %>%
dplyr::rename(ilr1_multi = ilr1, ilr2_multi = ilr2, ilr3_multi = ilr3) %>%
dplyr::mutate(SAND_multi = SAND, SILT_multi = SILT, CLAY_multi = CLAY)

bouy_multi_np <- bouy_multi %>%
```

```
dplyr::filter(., Treatment == "No peroxide") %>%
dplyr::rename(SAND_multi_np = SAND_multi, SILT_multi_np = SILT_multi, CLAY_multi_np = CLAY_multi, ilr1_m

bouy_multi_p <- bouy_multi %>%
dplyr::filter(., Treatment == "Peroxide") %>%
dplyr::rename(SAND_multi_p = SAND_multi, SILT_multi_p = SILT_multi, CLAY_multi_p = CLAY_multi, ilr1_mul

bouy_2pts_multi <- bouy_2pts_np[, c("IDEN", "ilr1_np", "ilr2_np", "ilr3_np", "SAND_np", "SILT_np", "CLA
left_join(bouy_multi[, c("IDEN", "Method_treatment", "ilr1_multi", "ilr2_multi", "ilr3_multi", "SAND_mul
  na.omit() %>%  unique(.) %>%
  mutate(ilr1_diff = ilr1_multi - ilr1_np, ilr2_diff = ilr2_multi - ilr2_np, ilr3_diff = ilr3_multi - il
```

Differences between sand percentages and with and without Bouyoucos's are calculated.

```
bouy_2pts_fract <- bouy_2pts_np[, c("IDEN", "SAND_np")] %>%
left_join(fract_sand[, c("IDEN", "Method_treatment", "SAND")], by = "IDEN") %>%
  na.omit() %>% unique(.) %>%
  mutate(ilr1_diff = NA, ilr2_diff = NA, ilr3_diff = NA, SAND_diff = SAND - SAND_np, SILT_diff = NA, CLA
```

Combine all differences

```
data_diff <- as.data.frame(rbind.data.frame(bouy_2pts_np_p[, c("IDEN", "Method_treatment", "ilr1_diff",
                                    bouy_2pts_np_0[, c("IDEN", "Method_treatment", "ilr1_diff", "ilr2_di
                                    bouy_2pts_np_2[, c("IDEN", "Method_treatment", "ilr1_diff", "ilr2_di
                                    bouy_2pts_multi[, c("IDEN", "Method_treatment", "ilr1_diff", "ilr2_
                                    bouy_2pts_fract[, c("IDEN", "Method_treatment", "ilr1_diff", "ilr2_
```

Combine all

```
perc_ilr_fract <- bouy_2pts_np[, c("IDEN", "SAND_np", "SILT_np", "CLAY_np")] %>%
  left_join(bouy_2pts_np_p[, c("IDEN", "SAND_p", "SILT_p", "CLAY_p")], by = "IDEN") %>%
  left_join(bouy_2pts_np_0[, c("IDEN", "SAND_0", "SILT_0", "CLAY_0")], by = "IDEN") %>%
  left_join(bouy_2pts_np_2[, c("IDEN", "SAND_2", "SILT_2", "CLAY_2")], by = "IDEN") %>%
  left_join(bouy_multi_np[, c("IDEN", "SAND_multi_np", "SILT_multi_np", "CLAY_multi_np")], by = "IDEN")
  left_join(bouy_multi_p[, c("IDEN", "SAND_multi_p", "SILT_multi_p", "CLAY_multi_p")], by = "IDEN") %>%
  left_join(fract_sand[, c("IDEN", "SAND")], by = "IDEN") %>%
  unique(.)
```

Linear model

A function for linear model is made ìlr_ci'between ilr or percentage differences and a value fixed as 0 for comparison influenced by methods and pre-treatments. We introduce deviation bands for variables; probability of observation's lower value is setted at 2.5 and higher value at 97.5.

```
ilr_ci <- function() {
ci <- confint(lm_method, level = 0.95) %>%
  data.frame() %>%
  rownames_to_column(var="Variable") %>%
   rename(lower = X2.5.., upper = X97.5..) %>%
  mutate(est. = coef(lm_method)) %>%
```

```
  drop_na()

ci <- ci %>%
  dplyr::mutate(Variable = c("Bouyoucos Peroxide", "Bouyoucos multi-point No peroxide", "Bouyoucos multi

return(ci)
}
```

Function `ilr_ci` is applied for all differences. Because sand percentage has an additionnal sieving method to apply, we considered it appart from function. ilr1 is `[C | CLAY,SILT,SAND]`. It is shown here as complement as it is not shown for publication.

ilrs

```
# ilr1
lm_method <- lm(ilr1_diff ~ 0 + Method_treatment, data_diff)
ci_ilr1 <- ilr_ci()
ci_ilr1$ilr <- c("`[C|CLAY,SILT,SAND]`")

# ilr2
lm_method <- lm(ilr2_diff ~ 0 + Method_treatment, data_diff)
ci_ilr2 <- ilr_ci()
ci_ilr2$ilr <- c("`[CLAY|SILT,SAND]`")

# ilr3
lm_method <- lm(ilr3_diff ~ 0 + Method_treatment, data_diff)
ci_ilr3 <- ilr_ci()
ci_ilr3$ilr <- c("`[SILT|SAND]`")
```

Percentages

```
# SAND
lm_method <- lm(SAND_diff ~ 0 + Method_treatment, data_diff)

ci_sand <- confint(lm_method, level = 0.95) %>%
  data.frame() %>%
  rownames_to_column(var="Variable") %>%
  rename(lower = X2.5.., upper = X97.5..) %>%
  mutate(est. = coef(lm_method)) %>%
  drop_na()

ci_sand <- ci_sand %>%
  dplyr::mutate(Variable = c("Bouyoucos Peroxide", "Bouyoucos multi-point No peroxide", "Bouyoucos multi

ci_sand$ilr <- c("SAND")

# SILT
lm_method <- lm(SILT_diff ~ 0 + Method_treatment, data_diff)
ci_silt <- ilr_ci()
ci_silt$ilr <- c("SILT")
```

```
# CLAY
lm_method <- lm(CLAY_diff ~ 0 + Method_treatment, data_diff)
ci_clay <- ilr_ci()
ci_clay$ilr <- c("CLAY")
```

Ilrs and percentages were combined in two distinct objects.

Ilrs

```
ci <- rbind.data.frame(ci_ilr1, ci_ilr2, ci_ilr3)

ci$Variable <- factor(ci$Variable, levels =c("Sieving", "Laser 2 min sonication", "Laser 0 min sonicati
```

Percentages

```
ci_perc <- rbind.data.frame(ci_sand, ci_silt, ci_clay)

ci_perc$Variable <- factor(ci_perc$Variable, levels =c("Sieving", "Laser 2 min sonication", "Laser 0 mi

ci_perc$ilr <- factor(ci_perc$ilr, levels =c("CLAY", "SILT", "SAND"))
```

Visualization

Plot's purpose is to show difference between samples for sand, silt and clay percentages, as ilr transformed values and percentages.

Ilrs

```
gg_ci_tot <- ggplot(ci) +
  geom_vline(xintercept = 0, lwd = 0.1) +
  facet_grid(ilr ~ ., scales = "free", labeller = label_parsed, switch = "x") +
  geom_segment(aes(x = lower, xend=upper, y = Variable, yend = Variable), lwd = 2, colour = "grey") +
  geom_point(aes(x = est., y = Variable), shape=25, fill='black', size=0.95) +
  geom_text(aes(x = est., y = Variable, label = format(round(est., 2), nsmall = 2)),
            size = 3, colour = "black", vjust=-1) +
  geom_text(aes(x = lower, y = Variable, label = format(round(lower, 2), nsmall = 2)), size = 3, hjust =
  geom_text(aes(x = upper, y = Variable, label = format(round(upper, 2), nsmall = 2)), size = 3, hjust =
  labs(x = "Effect", y = "") +
  expand_limits(x=c(-1.3, 3.8)) +
  theme(strip.text.y = element_text(angle = 0))

gg_ci_tot
```

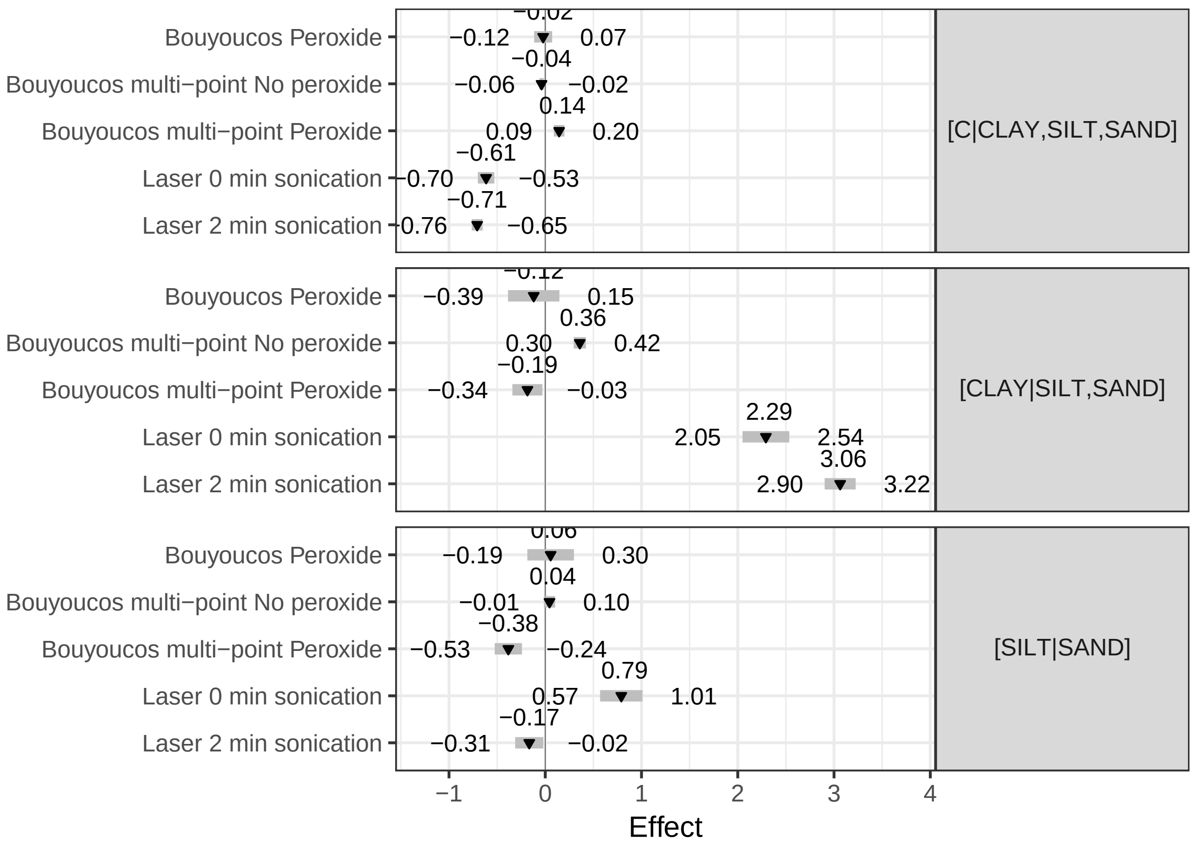


```r
ggsave("images/methods_ci.tiff", width = 14, height = 7.5, dpi = 300)
```

Percentages

```r
gg_ci_perc <- ggplot(ci_perc) +
  geom_vline(xintercept = 0, lwd = 0.1) +
  facet_grid(ilr ~ ., scales = "free", labeller = label_parsed, switch = "x") +
  geom_segment(aes(x = lower, xend=upper, y = Variable, yend = Variable), lwd = 2, colour = "grey") +
  geom_point(aes(x = est., y = Variable), shape=25, fill='black', size=0.95) +
  geom_text(aes(x = est., y = Variable, label = format(round(est., 2), nsmall = 2)),
            size = 3, colour = "black", vjust=-1) +
  geom_text(aes(x = lower, y = Variable, label = format(round(lower, 2), nsmall = 2)), size = 3, hjust =
  geom_text(aes(x = upper, y = Variable, label = format(round(upper, 2), nsmall = 2)), size = 3, hjust =
  labs(x = "Effect", y = "") +
  expand_limits(x=c(-32, 38)) +
  theme(strip.text.y = element_text(angle = 0))

gg_ci_perc
```

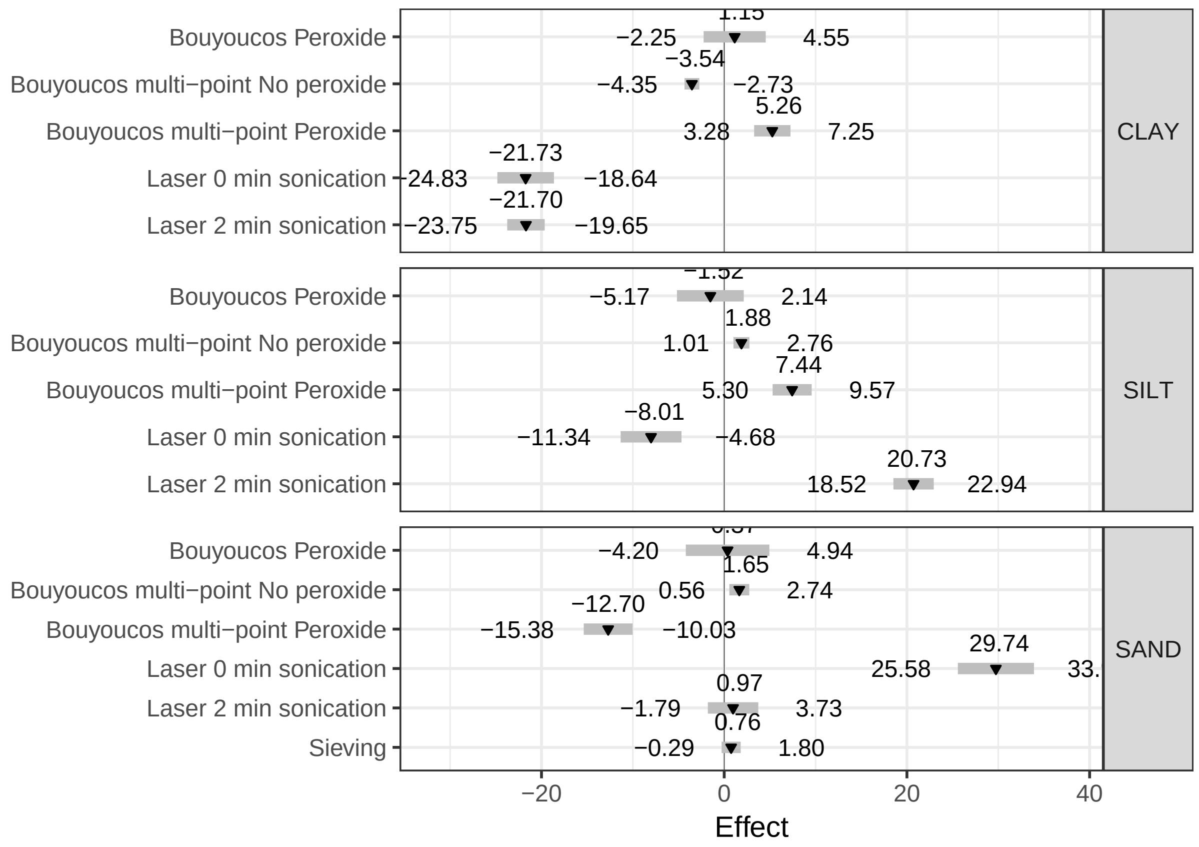


```r
ggsave("images/methods_ci_perc.tiff", width = 14, height = 7.5, dpi = 300)
```

1.2 - Differences between peroxide and no peroxide in Bouyoucos

We use both Bouyoucos and Bouyoucos estimated with and without peroxide this 2-points 2-h Bouyoucos set.

Visualization

We plot each ilr for normality.

```r
bouy_2pts_np_p %>%
  ggplot(aes(x = ilr1_diff)) +
  geom_histogram(aes(y=..density..), alpha=0.5) +
  geom_density(alpha=.5, fill="#FF6666")
```

```
## `stat_bin()` using `bins = 30`. Pick better value with `binwidth`.
```

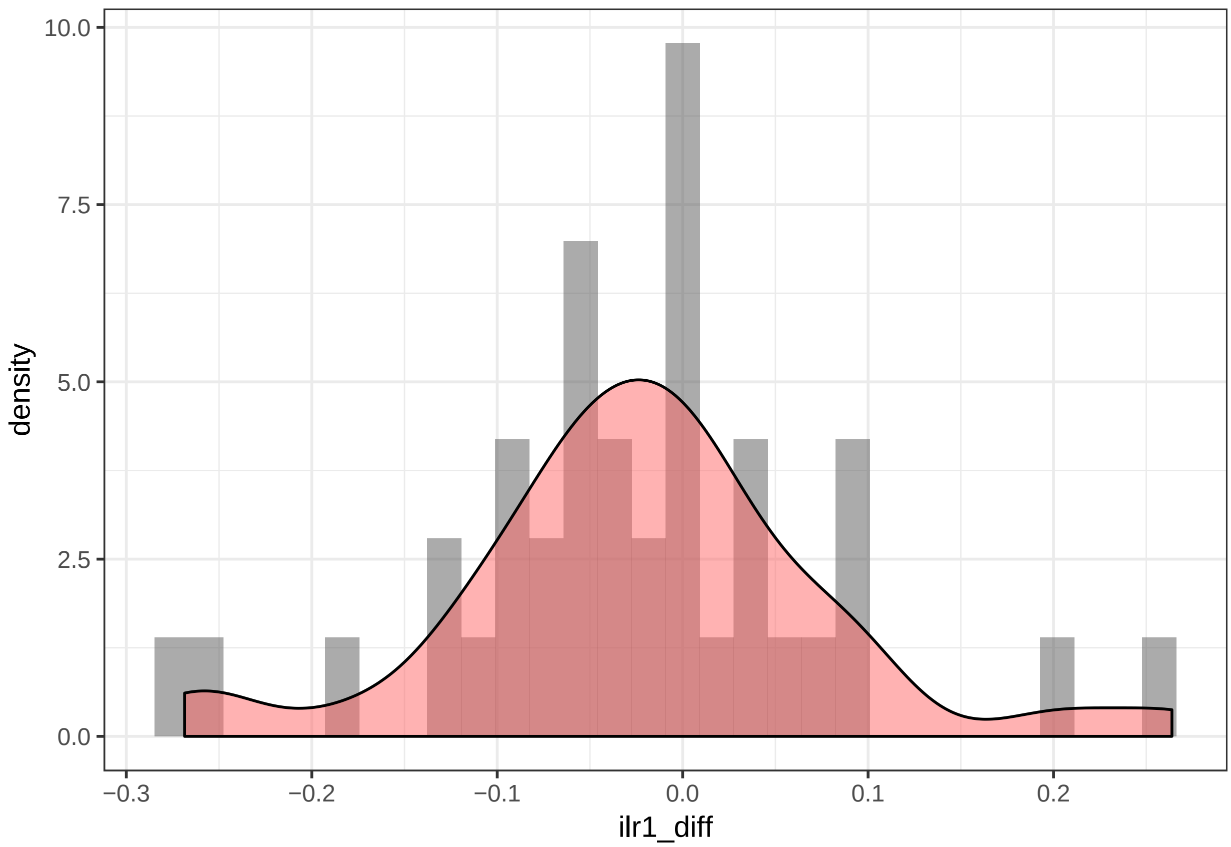


```
bouy_2pts_np_p %>%
  ggplot(aes(x = ilr2_diff)) +
  geom_histogram(aes(y=..density..), alpha=0.5) +
  geom_density(alpha=.5, fill="#FF6666")
```

```
## `stat_bin()` using `bins = 30`. Pick better value with `binwidth`.
```

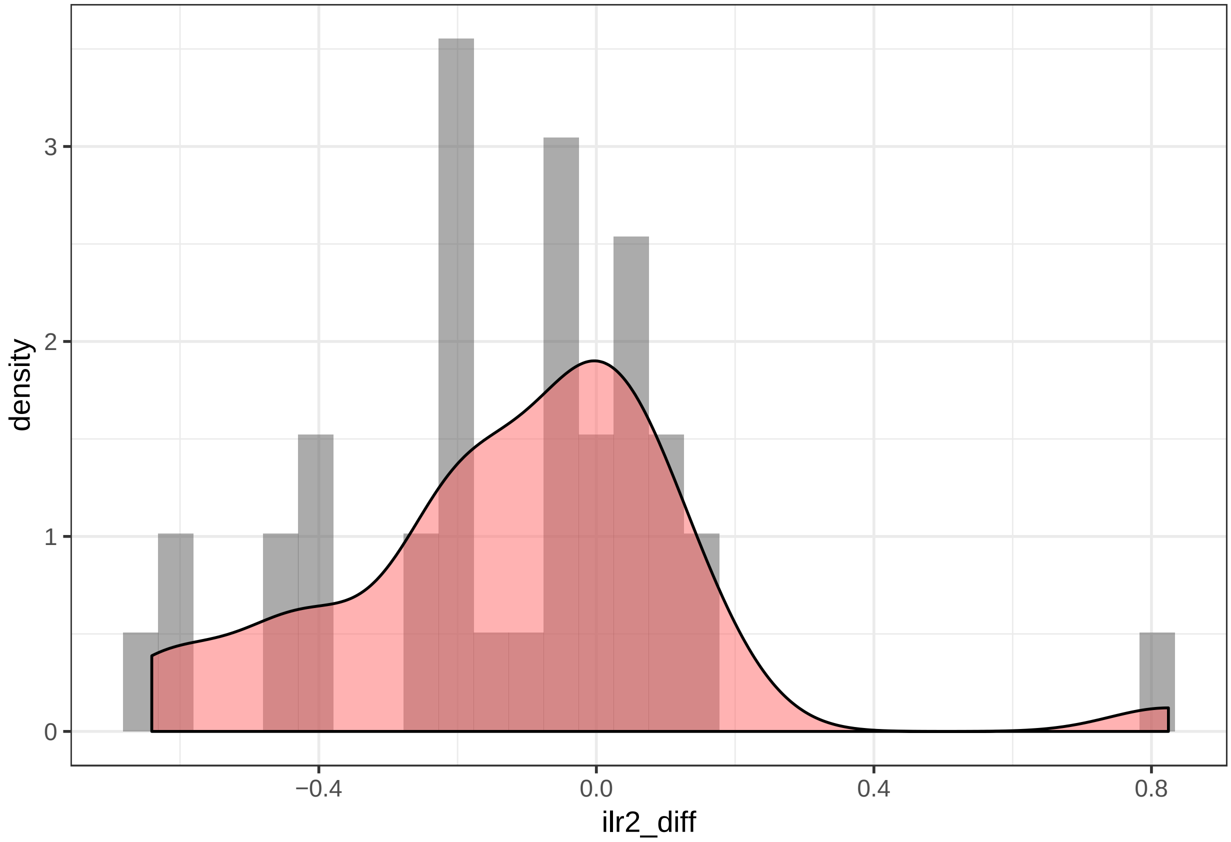


```
bouy_2pts_np_p %>%
  ggplot(aes(x = ilr3_diff)) +
  geom_histogram(aes(y=..density..), alpha=0.5) +
  geom_density(alpha=.5, fill="#FF6666")
```

```
## `stat_bin()` using `bins = 30`. Pick better value with `binwidth`.
```

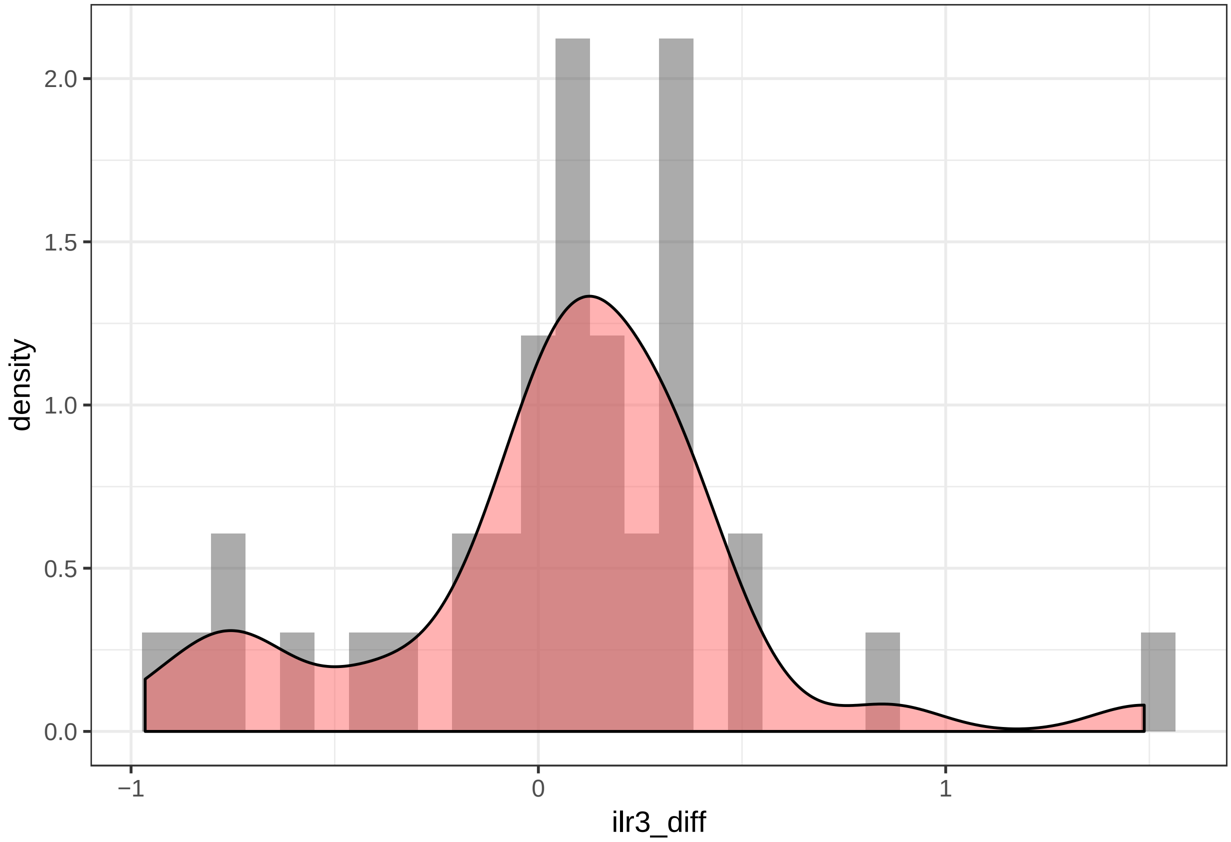

Significant differences

We test for differences with 't.test' using function `test_paired`.

```
test_paired <- function(data, ilr1_x, ilr2_x, ilr3_x, ilr1_y, ilr2_y, ilr3_y) {

test1 <- t.test(x = ilr1_x, y = ilr1_y, paired = TRUE)
test2 <- t.test(x = ilr2_x, y = ilr2_y, paired = TRUE)
test3 <- t.test(x = ilr3_x, y = ilr3_y, paired = TRUE)

lm_xy <- lm(ilr1_x ~ ilr1_y, data)

gg <- ggplot(data, mapping = aes(x = ilr2_x, y = ilr2_y)) +
  #facet_wrap(~Prediction, ncol=3) +
  #geom_point(mapping = aes(colour = Prediction), alpha = 0.5) +
  theme_classic() +
  geom_abline(aes(intercept = 0, slope = 1)) +
  geom_smooth(method = lm) +
  geom_count()

  print(gg)

tab <- map_df(list(test1, test2, test3), tidy)
tab$Stat <- c("Ilr1", "Ilr2", "Ilr3")
```

```
  return(tab)

}
```

A paired t.test between no peroxided and others

peroxide

```
t1 <- test_paired(bouy_2pts_np_p, bouy_2pts_np_p$ilr1_np, bouy_2pts_np_p$ilr2_np, bouy_2pts_np_p$ilr3_np
```

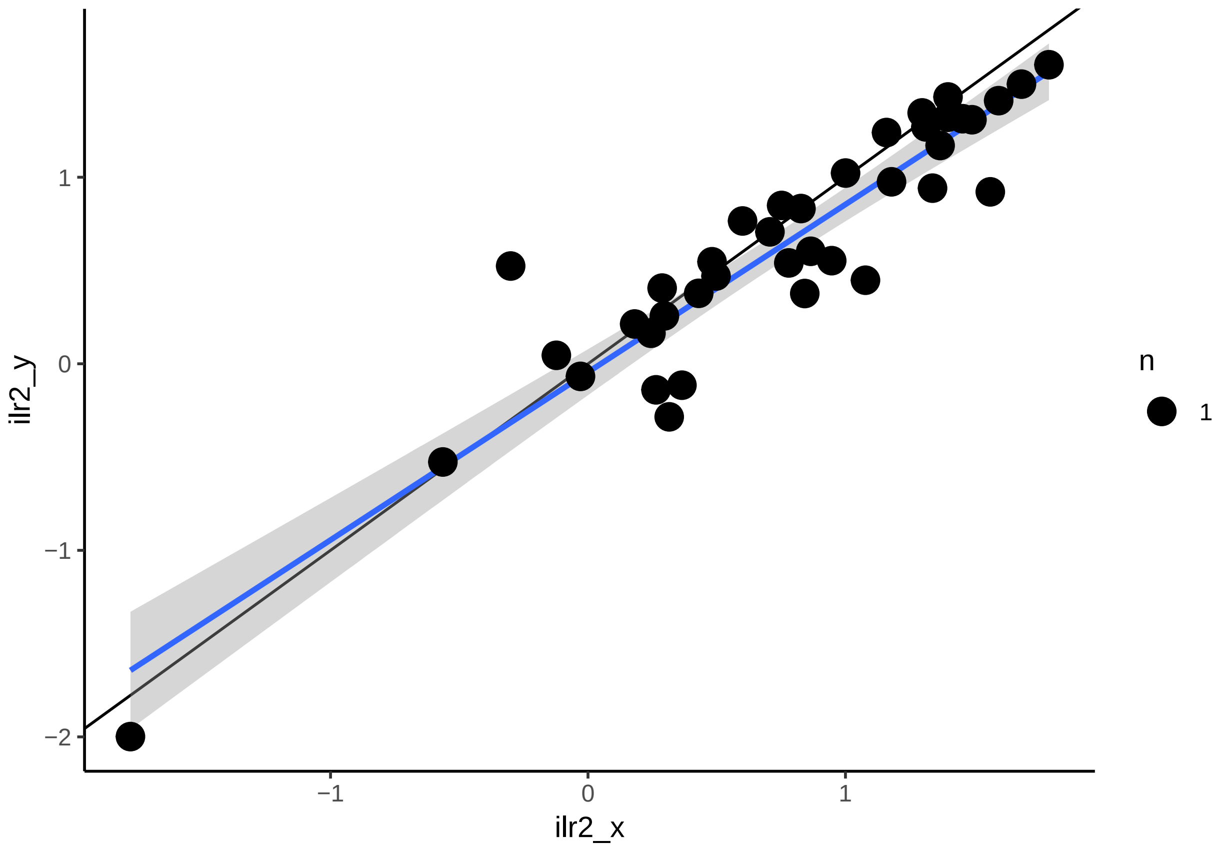


```
t1$Method1 <- c("Bouyoucos No peroxide")
t1$Method2 <- c("Bouyoucos Peroxide")
```

multi no peroxide

```
bouy_np_multi_np <- bouy_2pts_np[, c("IDEN", "ilr1_np", "ilr2_np", "ilr3_np")] %>%
left_join(bouy_multi_np)
```

```
## Joining, by = "IDEN"
```

```r
t2 <- test_paired(bouy_np_multi_np, bouy_np_multi_np$ilr1_np, bouy_np_multi_np$ilr2_np, bouy_np_multi_np
```

```
## Warning: Removed 427 rows containing non-finite values (stat_smooth).
```

```
## Warning: Removed 427 rows containing non-finite values (stat_sum).
```

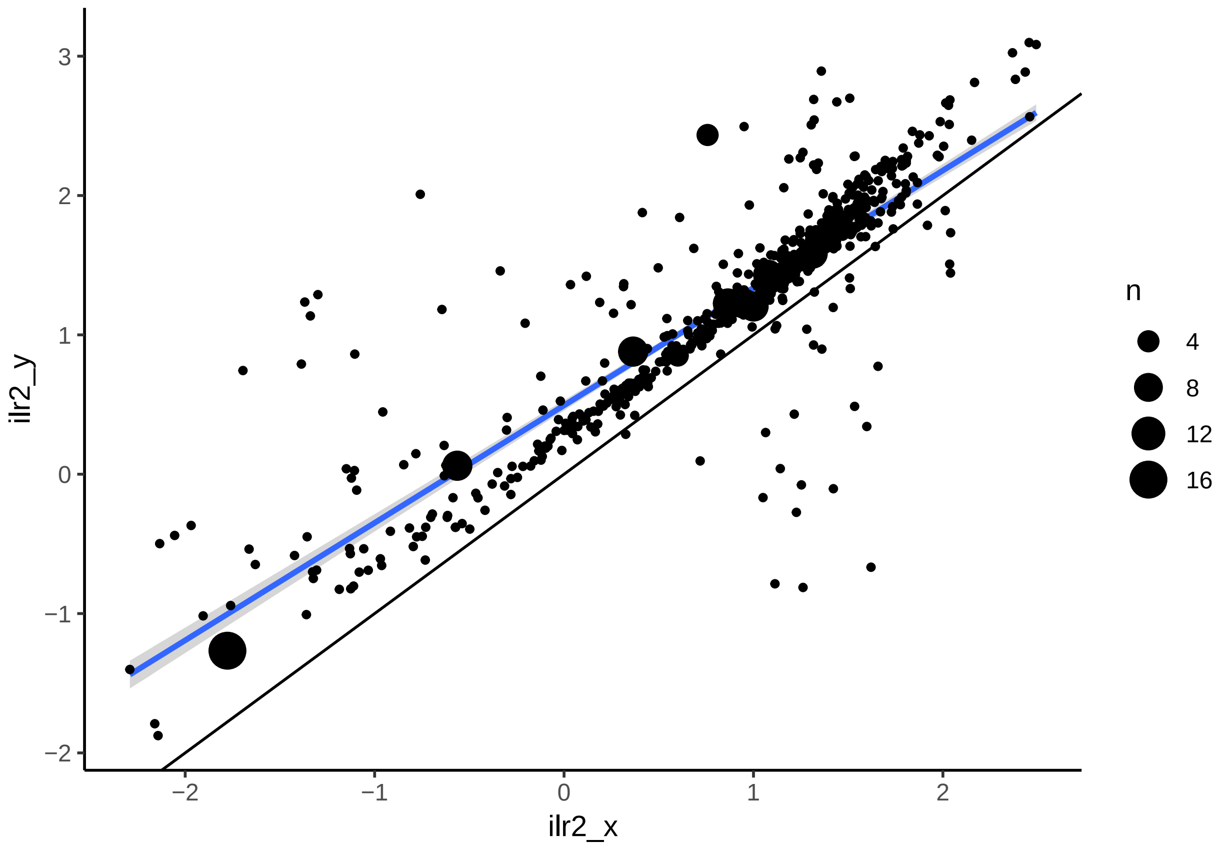


```r
t2$Method1 <- c("Bouyoucos No peroxide")
t2$Method2 <- c("Bouyoucos multi No peroxide")
```

multi peroxide

```r
bouy_p_multi_p <- bouy_2pts_p[, c("IDEN", "ilr1_p", "ilr2_p", "ilr3_p")] %>%
left_join(bouy_multi_p)
```

```
## Joining, by = "IDEN"
```

```r
t3 <- test_paired(bouy_p_multi_p, bouy_p_multi_p$ilr1_p, bouy_p_multi_p$ilr2_p, bouy_p_multi_p$ilr3_p, b
```

```
## Warning: Removed 22 rows containing non-finite values (stat_smooth).
```

```
## Warning: Removed 22 rows containing non-finite values (stat_sum).
```

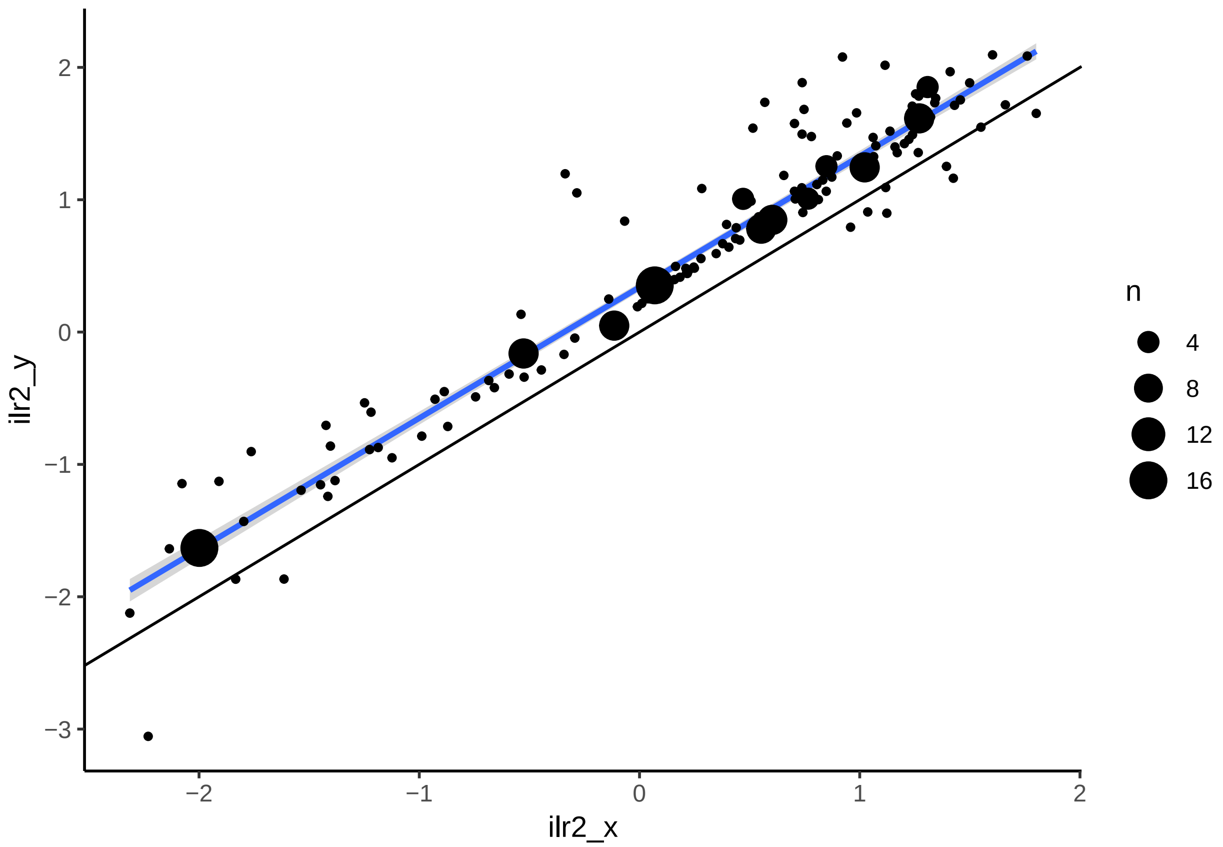


```
t3$Method1 <- c("Bouyoucos Peroxide")
t3$Method2 <- c("Bouyoucos multi Peroxide")
```

laser 0 min

```
t4 <- test_paired(bouy_2pts_np_0, bouy_2pts_np_0$ilr1_np, bouy_2pts_np_0$ilr2_np, bouy_2pts_np_0$ilr3_np
```

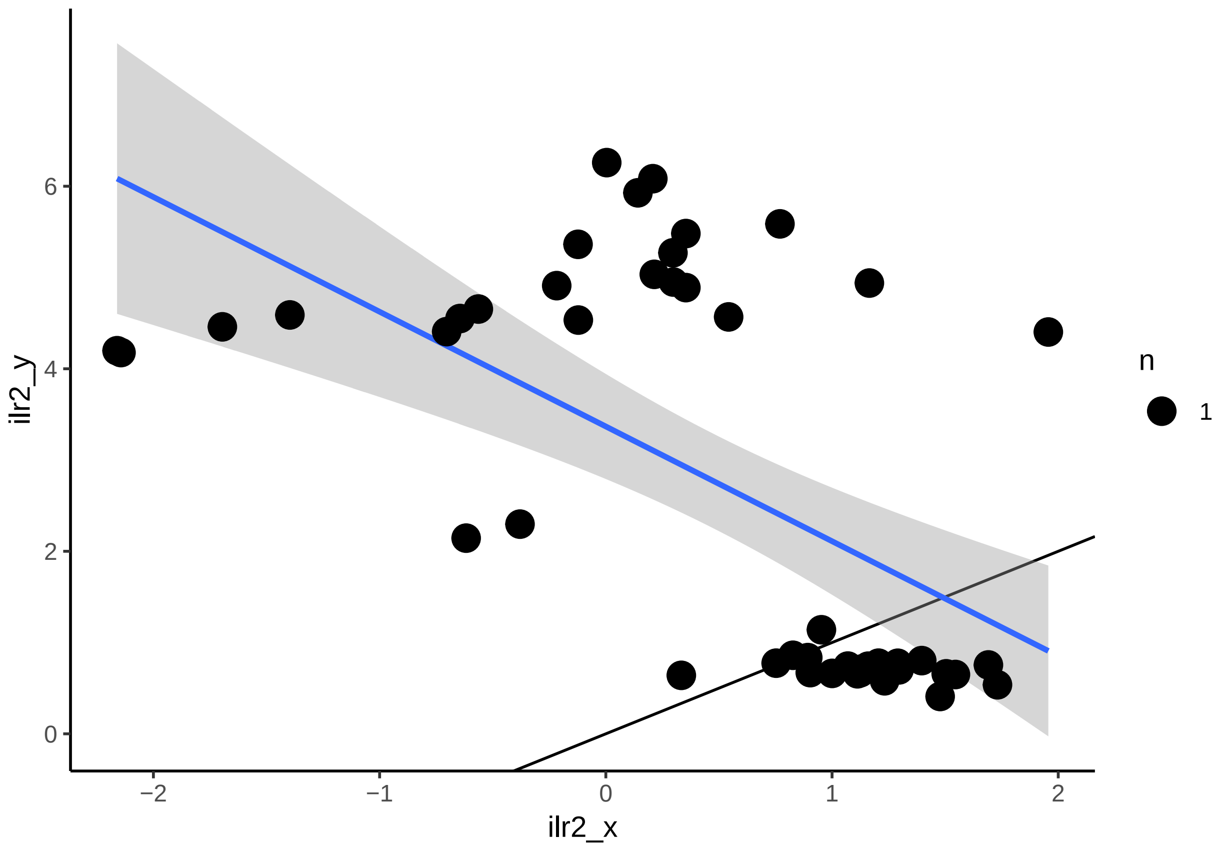


```
t4$Method1 <- c("Bouyoucos No peroxide")
t4$Method2 <- c("Laser 0 min")
```

laser 2 min

```
t5 <- test_paired(bouy_2pts_np_2, bouy_2pts_np_2$ilr1_np, bouy_2pts_np_2$ilr2_np, bouy_2pts_np_2$ilr3_n
```

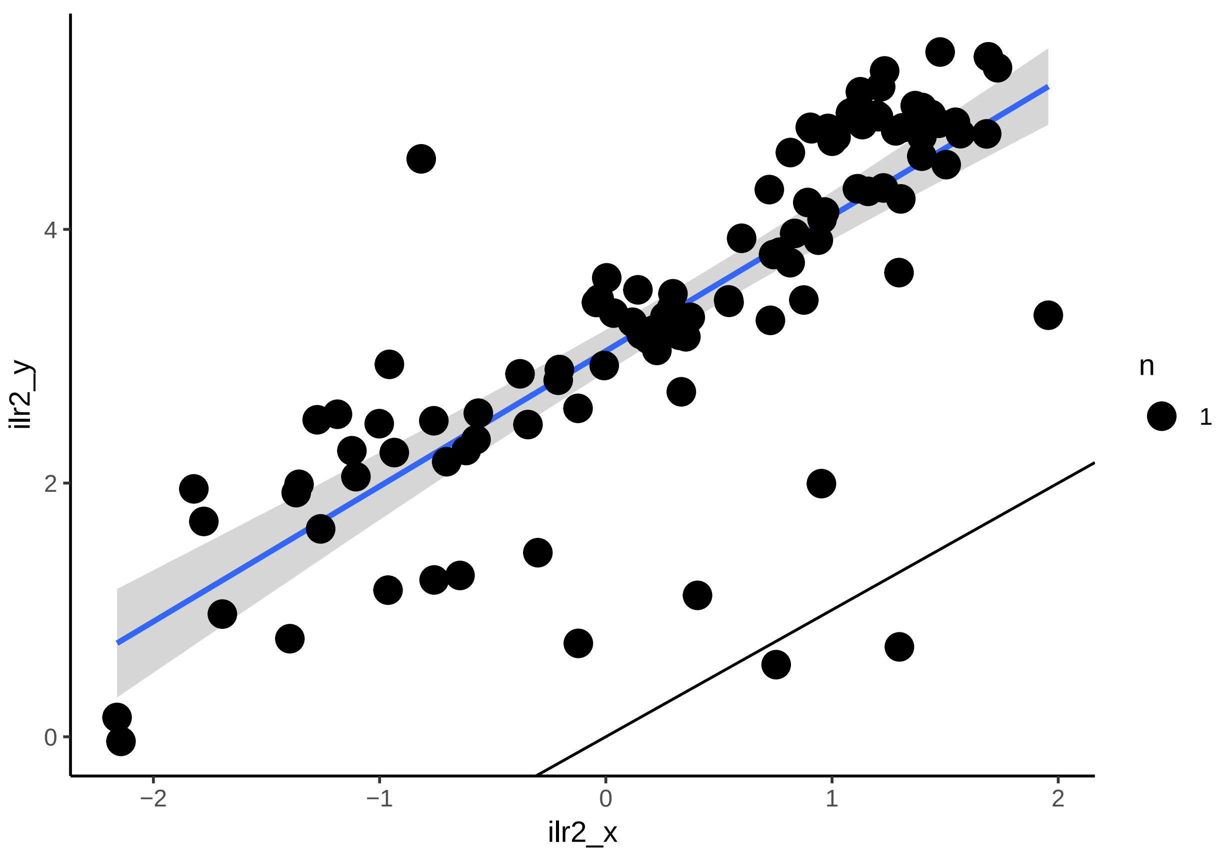


```
t5$Method1 <- c("Bouyoucos No peroxide")
t5$Method2 <- c("Laser 2 min")
```

1.4 - Differences between Bouyoucos and sand fractions mesured using sieving methodology

We select sand percentages. Differences between Bouyoucos and sieving's sand percentages are calculated.

A paired t.test

Between sieving and other methods

```
test_paired_sand <- function(data, sand_x, sand_y) {

test1 <- t.test(x = sand_x, y = sand_y, paired = TRUE)

lm_xy <- lm(sand_x ~ sand_y, data)

gg <- ggplot(data, mapping = aes(x = sand_x, y = sand_y)) +
  #facet_wrap(~Prediction, ncol=1) +
  #geom_point(mapping = aes(colour = Prediction), alpha = 0.5) +
```

```r
  theme_classic() +
  geom_abline(aes(intercept = 0, slope = 1)) +
  geom_smooth(method = lm) +
  geom_count()

  print(gg)

tab <- map_df(list(test1), tidy)
tab$Method1 <- c("Sieving")
tab$Stat <- c("Percentage")

  return(tab)

}

t6 <- test_paired_sand(perc_ilr_fract, perc_ilr_fract$SAND, perc_ilr_fract$SAND_np)
```

```
## Warning: Removed 357 rows containing non-finite values (stat_smooth).

## Warning: Removed 357 rows containing non-finite values (stat_sum).
```

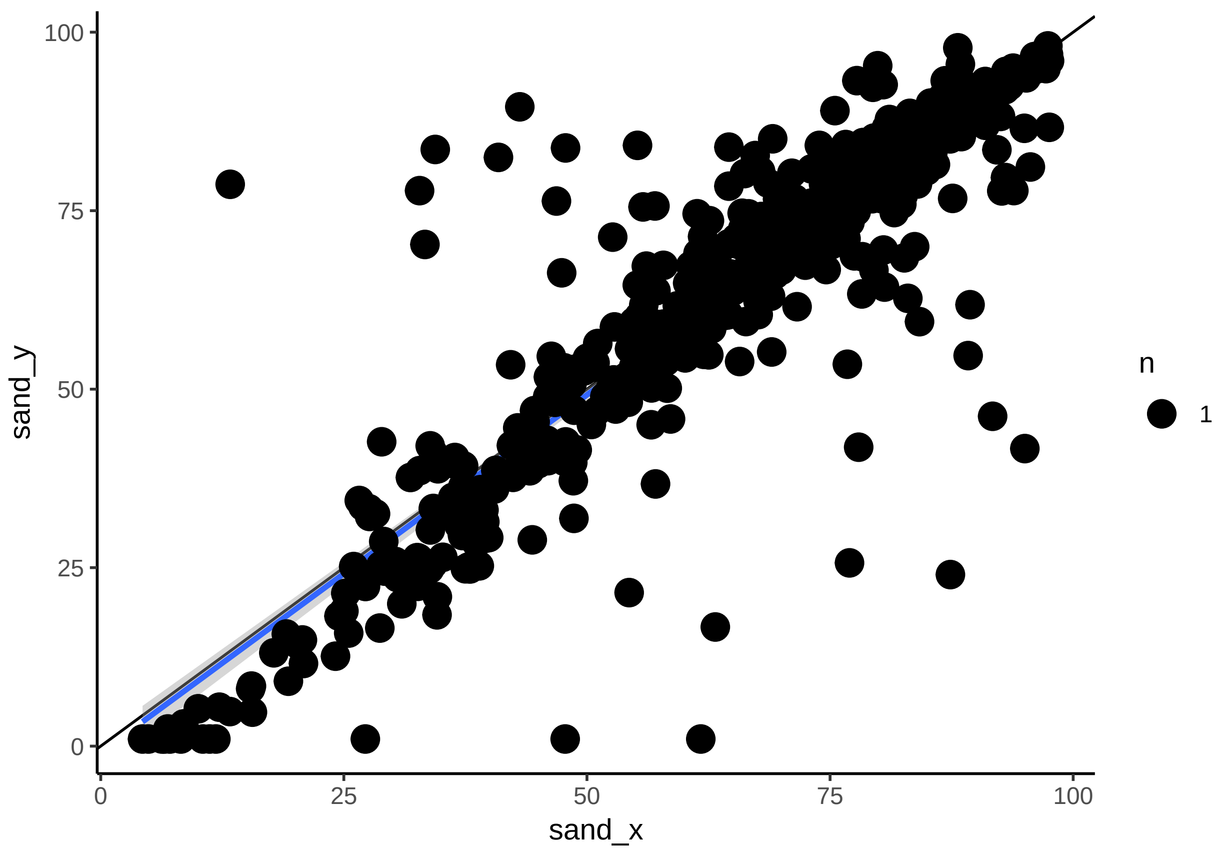


```r
t7 <- test_paired_sand(perc_ilr_fract, perc_ilr_fract$SAND, perc_ilr_fract$SAND_p)
```

```
## Warning: Removed 1077 rows containing non-finite values (stat_smooth).
```

```
## Warning: Removed 1077 rows containing non-finite values (stat_sum).
```

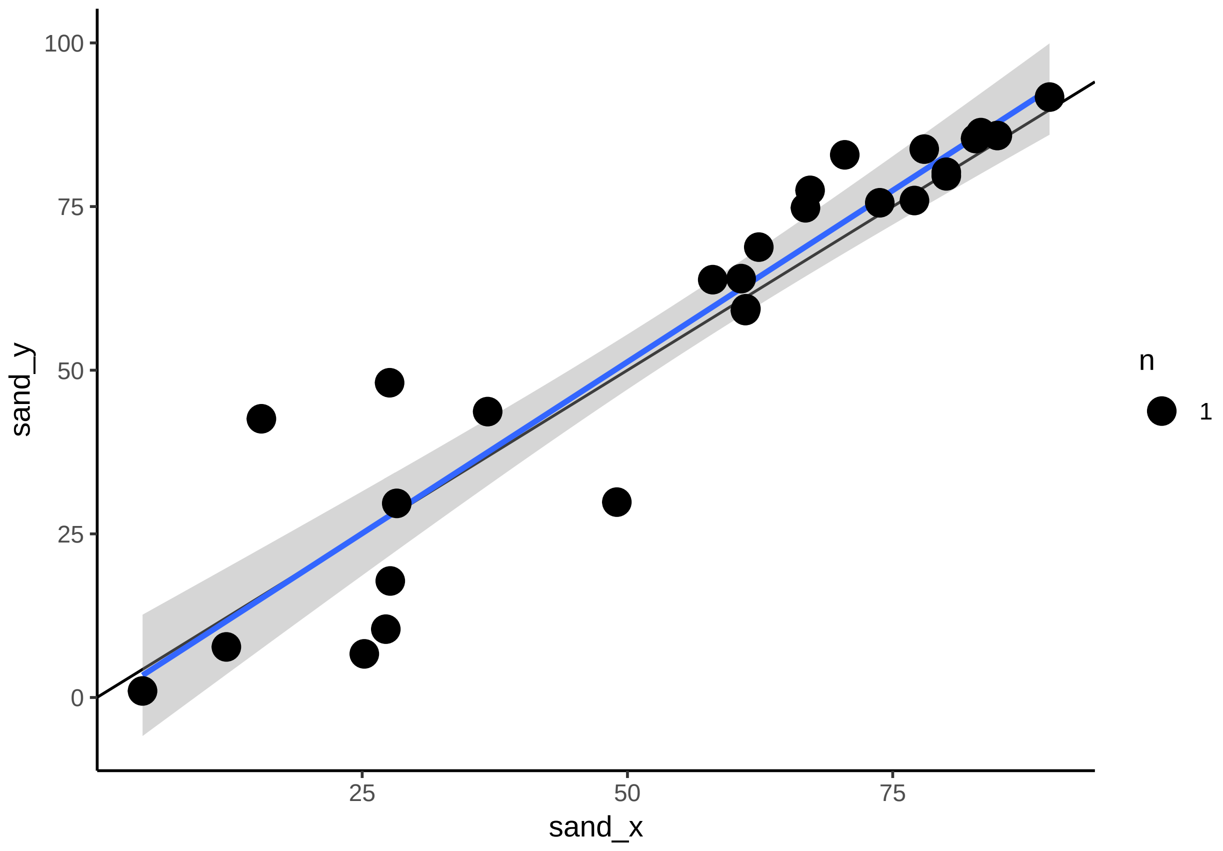


```
t8 <- test_paired_sand(perc_ilr_fract, perc_ilr_fract$SAND, perc_ilr_fract$SAND_0)
```

```
## Warning: Removed 1069 rows containing non-finite values (stat_smooth).

## Warning: Removed 1069 rows containing non-finite values (stat_sum).
```

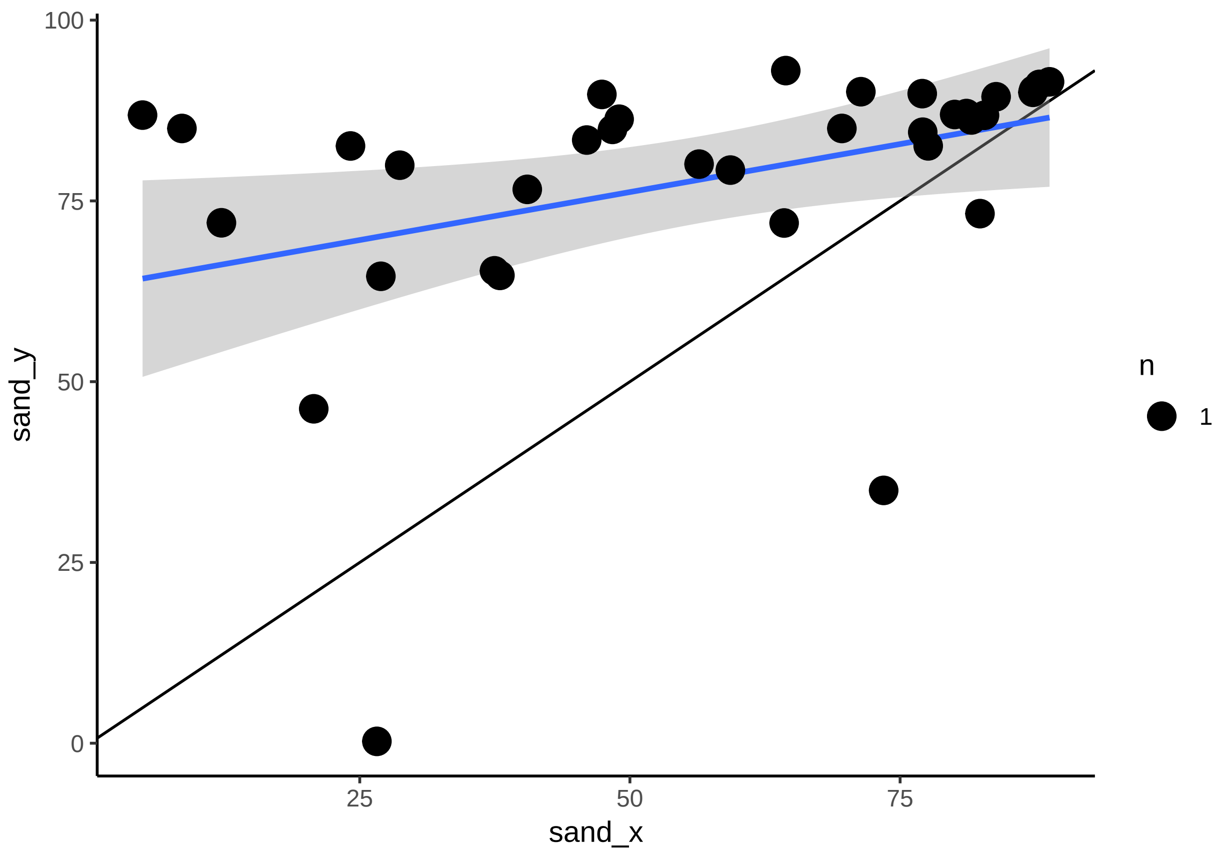


```
t9 <- test_paired_sand(perc_ilr_fract, perc_ilr_fract$SAND, perc_ilr_fract$SAND_2)
```

```
## Warning: Removed 1026 rows containing non-finite values (stat_smooth).
```

```
## Warning: Removed 1026 rows containing non-finite values (stat_sum).
```

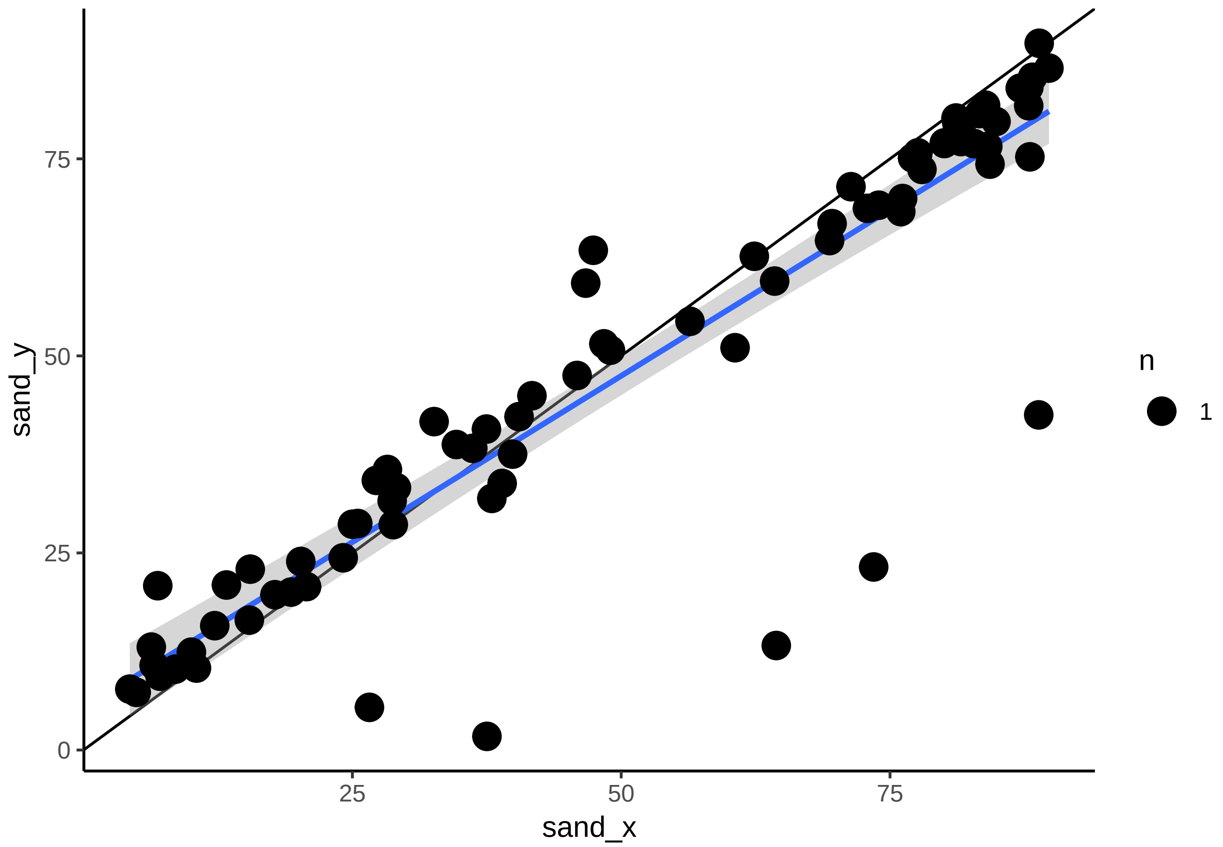


```
t10 <- test_paired_sand(perc_ilr_fract, perc_ilr_fract$SAND, perc_ilr_fract$SAND_multi_np)
```

```
## Warning: Removed 745 rows containing non-finite values (stat_smooth).
```

```
## Warning: Removed 745 rows containing non-finite values (stat_sum).
```

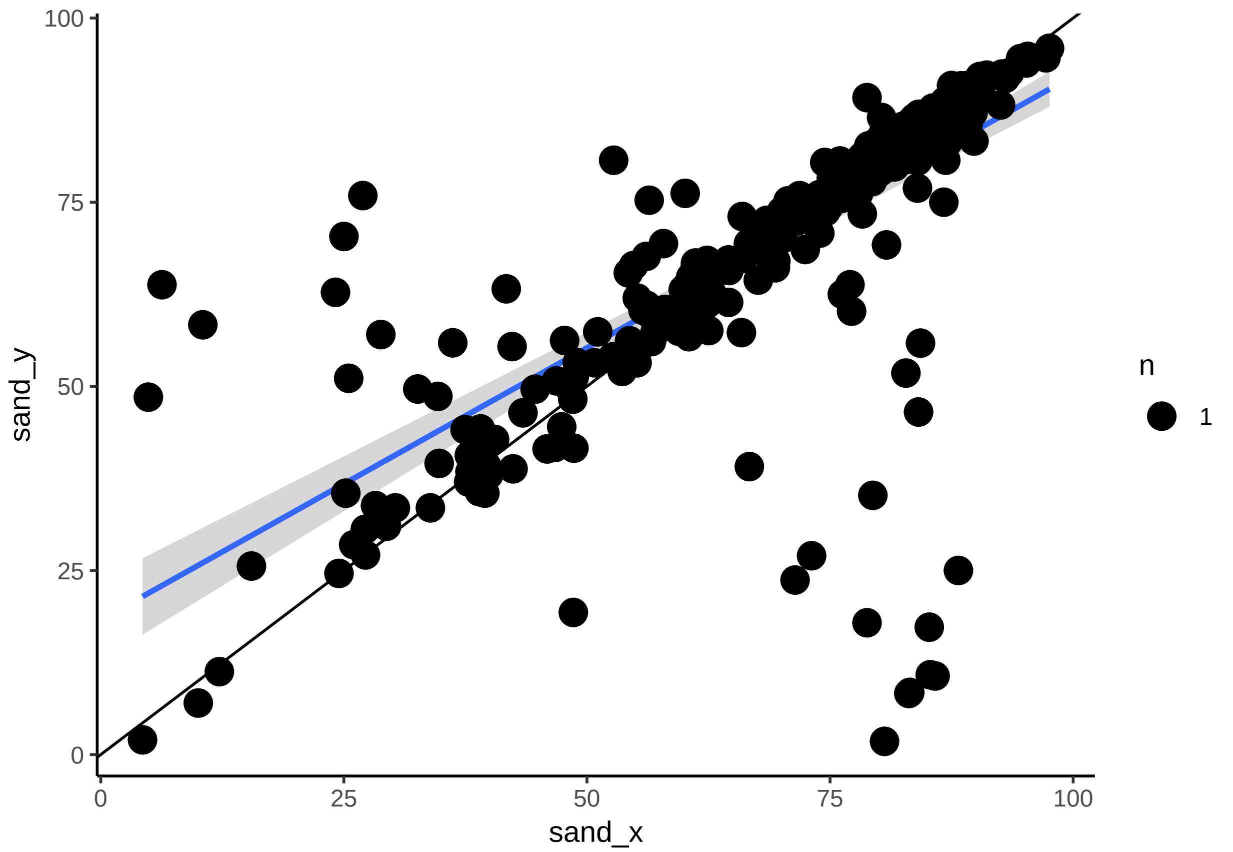


```
t11 <- test_paired_sand(perc_ilr_fract, perc_ilr_fract$SAND, perc_ilr_fract$SAND_multi_p)
```

```
## Warning: Removed 1019 rows containing non-finite values (stat_smooth).
```

```
## Warning: Removed 1019 rows containing non-finite values (stat_sum).
```

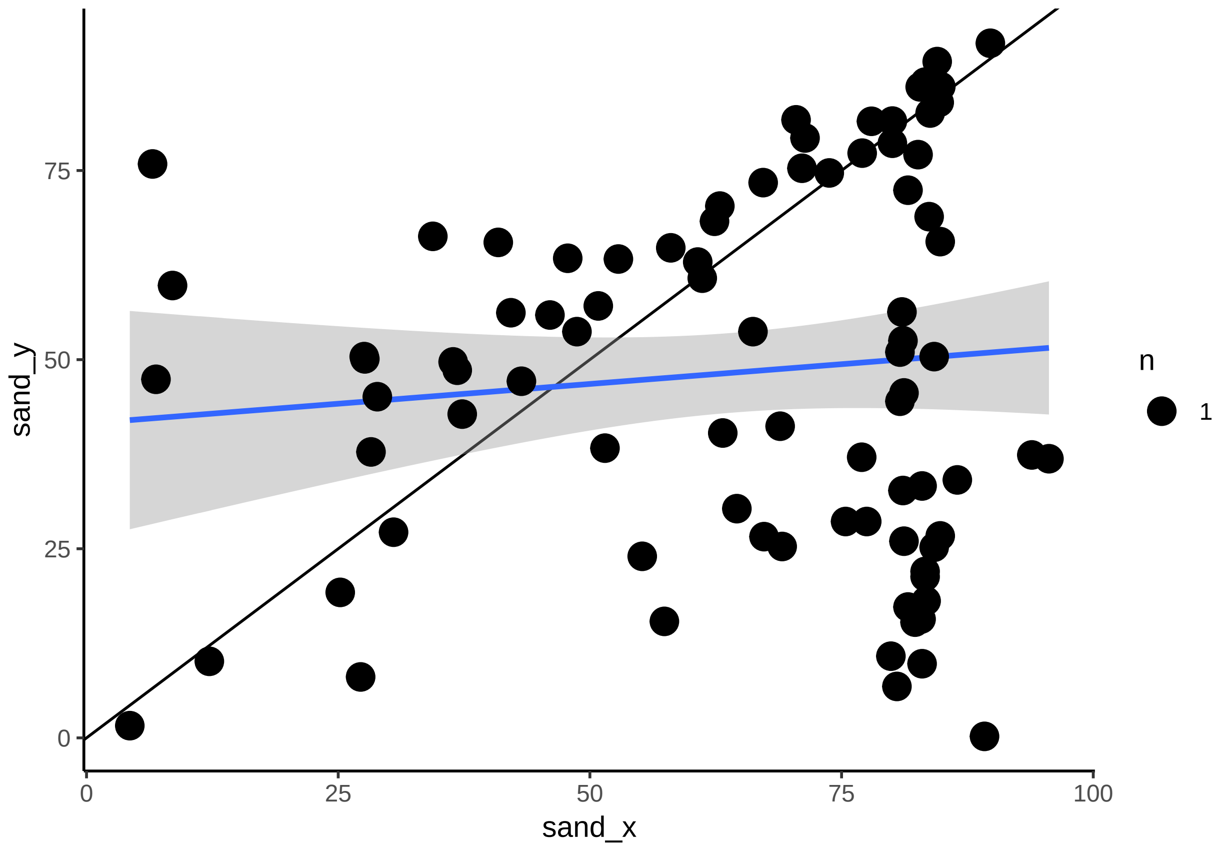

We name second method for sand percentages comparison.

```
t6$Method2 <- c("Bouyoucos No peroxide")
t7$Method2 <- c("Bouyoucos Peroxide")
t8$Method2 <- c("Laser 0 min")
t9$Method2 <- c("Laser 2 min")
t10$Method2 <- c("Bouyoucos multi No peroxide")
t11$Method2 <- c("Bouyoucos multi Peroxide")
```

p-value

Combine all p-values from t.test.

```
tab <-as.data.frame(t1) %>% full_join(.,as.data.frame(t2)) %>%
full_join(.,as.data.frame(t3), by = c("estimate", "statistic", "p.value", "parameter", "conf.low", "con
full_join(.,as.data.frame(t4), by = c("estimate", "statistic", "p.value", "parameter", "conf.low", "con
full_join(.,as.data.frame(t5), by = c("estimate", "statistic", "p.value", "parameter", "conf.low", "con
full_join(.,as.data.frame(t6), by = c("estimate", "statistic", "p.value", "parameter", "conf.low", "con
full_join(.,as.data.frame(t7), by = c("estimate", "statistic", "p.value", "parameter", "conf.low", "con
full_join(.,as.data.frame(t8), by = c("estimate", "statistic", "p.value", "parameter", "conf.low", "con
full_join(.,as.data.frame(t9), by = c("estimate", "statistic", "p.value", "parameter", "conf.low", "con
full_join(.,as.data.frame(t10), by = c("estimate", "statistic", "p.value", "parameter", "conf.low", "co
full_join(.,as.data.frame(t11), by = c("estimate", "statistic", "p.value", "parameter", "conf.low", "co
```

```
## Joining, by = c("estimate", "statistic", "p.value", "parameter", "conf.low", "conf.high", "method",
```

Save results in a table.

```
write_csv(x=tab, path = "output/tab.csv")
```

Question 2

Predict sand, silt and clay from IR data

The samples' identifications `IDEN` are typed as character.

```
time_coefficients$IDEN <- as.character(time_coefficients$IDEN)
c_density$IDEN <- as.character(c_density$IDEN)
color_rgb$IDEN <- as.character(color_rgb$IDEN)
bal_oxalate$IDEN <- as.character(bal_oxalate$IDEN)
NIR_scores$IDEN <- as.character(NIR_scores$IDEN)
NIR4X_scores$IDEN <- as.character(NIR4X_scores$IDEN)
```

Features are defined to select or unselect specific parameters.

```
features <- c("Density_gPcm3", "r", "g", "b", "ilr1_ox", "ilr2_ox", "ilr3_ox", "ilr4_ox", "ilr5_ox", "il
```

We remove outliers from principal data frame.

```
#is_in = sign2(perc_ilr_feat_2xscores[, c("SAND", "SILT", "CLAY", "C_perc")], qcrit = 0.95)$wfinal01 ==
#is_in = rep(TRUE, nrow(perc_ilr_feat_2xscores))
#sum(is_in)/length(is_in)

#outliers <- boxplot(perc_ilr_feat_2xscores[, c("C_perc", "CLAY", "SILT", "SAND")], plot=FALSE)$out
data_out <-perc_ilr_feat_2xscores
#data_out <- data_out[-which(data_out$C_perc %in% outliers),]

remove_outliers <- function(x, na.rm = TRUE) {
  qnt <- quantile(x, probs=c(.025, .975), na.rm = na.rm)
  H <- 1.5 * IQR(x, na.rm = na.rm)
  y <- x
  y[x < (qnt[1] - H)] <- NA
  y[x > (qnt[2] + H)] <- NA
  y
}
is_rm <- remove_outliers(perc_ilr_feat_2xscores$C_perc)
data_out_rm <- data_out[which(data_out$C_perc %in% is_rm),]
#summary(data_out_rm)
```

We join laser with NIR scores.

```
# Laser 2min - set1
data_ml_laser <- data_out_rm  %>%
  dplyr::select(., -features) %>%
  dplyr::filter(Method_treatment == c("laser   2   min")) %>%
  unique(.)
data_ml_laser <- data_ml_laser[complete.cases(data_ml_laser), ]
```

```r
# Laser with 2-points 2-h Bouyoucos - set2
data_ml_tot <- data_out_rm  %>%
  dplyr::select(., -features) %>%
  dplyr::filter(Method_treatment == c("Bouyoucos 2-point 2-h No peroxide", "Bouyoucos 2-point 2-h Perox
unique(.)
```

```
## Warning in Method_treatment == c("Bouyoucos 2-point 2-h No peroxide",
## "Bouyoucos 2-point 2-h Peroxide", : la taille d'un objet plus long n'est
## pas multiple de la taille d'un objet plus court
```

```r
data_ml_tot <- data_ml_tot[complete.cases(data_ml_tot), ]
```

```r
# Multi-points 7-h Bouyoucos - set3
data_ml_multipoint <- data_out_rm  %>%
  dplyr::select(., -features) %>%
  dplyr::filter(Method_treatment == c("Bouyoucos multi-point 7-h   No peroxide")) %>%
  unique(.)
data_ml_multipoint <- data_ml_multipoint[complete.cases(data_ml_multipoint), ]

data_ml_twopoint <- data_ml_multipoint[, c("IDEN")] %>%
  left_join(data_out_rm, by = "IDEN")  %>%
  dplyr::select(., -features) %>%
  dplyr::filter(Method_treatment == c("Bouyoucos 2-point 2-h No peroxide")) %>%
  unique(.)
data_ml_twopoint <- data_ml_twopoint[complete.cases(data_ml_twopoint),]

data_ml_multipoint <- data_ml_twopoint[, c("IDEN")] %>%
  left_join(., data_ml_multipoint, by = "IDEN") %>%
  unique(.)
data_ml_multipoint <- data_ml_multipoint[complete.cases(data_ml_multipoint), ]

data_ml_twopoint <- data_ml_multipoint[, c("IDEN")] %>%
  left_join(., data_ml_twopoint, by = "IDEN") %>%
   unique(.)
data_ml_twopoint <- data_ml_twopoint[complete.cases(data_ml_twopoint), ]
```

We join Bouyoucos with MIR scores.

```r
# 2-points 2-h Bouyoucos with MIR - set4
data_ml_MIR <- data_out_rm  %>%
  dplyr::select(., IDEN, IDEN_Method_treatment, Method, Treatment, Method_treatment, SAND, SILT, CLAY, 
  dplyr::filter(Method_treatment == c("Bouyoucos 2-point 2-h No peroxide")) %>%
  left_join(MIR_scores, by = "IDEN") %>%
  unique(.)
data_ml_MIR <- data_ml_MIR[complete.cases(data_ml_MIR), ]

data_ml_NIR <- data_ml_MIR[, c("IDEN")] %>%
  left_join(data_out_rm, by = "IDEN")  %>%
  dplyr::select(., -features) %>%
  dplyr::filter(Method_treatment == c("Bouyoucos 2-point 2-h No peroxide")) %>%
  unique(.)
data_ml_NIR <- data_ml_NIR[complete.cases(data_ml_NIR),]
```

```
data_ml_MIR <- data_ml_NIR[, c("IDEN")] %>%
  left_join(., data_ml_MIR, by = "IDEN") %>%
  unique(.)
data_ml_MIR <- data_ml_MIR[complete.cases(data_ml_MIR), ]
```

We combine 2-points 2-h Bouyoucos as ilrs with NIR 4X gain scores.

```
# 2-points 2-h Bouyoucos with NIR4X - set5
data_ml_NIR4X <- NIR4X_scores  %>%
  left_join(data_out_rm[, c("IDEN", "IDEN_Method_treatment", "Method", "Treatment", "Method_treatment",
  dplyr::filter(Method_treatment == c("Bouyoucos 2-point 2-h No peroxide")) %>%
  unique(.)
data_ml_NIR4X <- as.tibble(data_ml_NIR4X[complete.cases(data_ml_NIR4X), ])

data_ml_NIR2X <- data_ml_NIR4X[, c("IDEN")] %>%
  left_join(as.tibble(data_out_rm), by = "IDEN") %>%
  dplyr::filter(Method_treatment == c("Bouyoucos 2-point 2-h No peroxide")) %>%
  dplyr::select(., IDEN, IDEN_Method_treatment, Method, Treatment, Method_treatment, ilr1, ilr2, ilr3,
  left_join(., NIR_scores, by = "IDEN") %>%
  unique(.)
data_ml_NIR2X <- data_ml_NIR2X[complete.cases(data_ml_NIR2X),]

data_ml_NIR4X <- data_ml_NIR2X[, c("IDEN")] %>%
  left_join(., data_ml_NIR4X, by = "IDEN") %>%
  unique(.)
data_ml_NIR4X <- data_ml_NIR4X[complete.cases(data_ml_NIR4X), ]
```

Ilrs and coefficients (slope and intercept) are combined.

```
# slope and intercept from Bouyoucos - set6
data_out_rm_bouy2 <- data_out_rm %>%  dplyr::filter(Method== c("Bouyoucos 2-point 2-h"))
data_ml_coeff <- time_coefficients %>%
  left_join(., data_out_rm_bouy2, by = c("IDEN_Method_treatment", "IDEN", "Method", "Treatment", "CLAY"
  dplyr::select(., -features) %>%
  unique(.)
data_ml_coeff <- data_ml_coeff[complete.cases(data_ml_coeff), ]
```

We combine 2-points 2-h Bouyoucos as ilrs with features.

```
# 2-points 2-h Bouyoucos with features - set7
data_ml_feat <- data_out_rm %>%
  left_join(., ph_m3[, c("IDEN", "pHCaCl2")], by = "IDEN") %>%
  dplyr::filter(Method== c("Bouyoucos 2-point 2-h")) %>%
  unique(.)
data_ml_feat <- data_ml_feat[complete.cases(data_ml_feat), ]
```

We combine sand percentage from 2-points 2-h Bouyoucos with sieved sand.

```
# Sieved sand - set8
data_ml_sieve <- fract_sand[, c("IDEN_Method_treatment", "IDEN", "Method", "Treatment", "Method_treatme
  unique(.)
```

```r
data_ml_sieve <- data_ml_sieve[complete.cases(data_ml_sieve), ]

data_ml_sieve2 <- data_ml_sieve[, c("IDEN")] %>%
  left_join(., data_out_rm[, c("IDEN_Method_treatment", "IDEN", "Method", "Treatment", "Method_treatmen
  dplyr::filter(Method == c("Bouyoucos 2-point 2-h")) %>%
  unique(.) %>%
  left_join(., NIR_scores)
```

```
## Joining, by = "IDEN"
## Joining, by = "IDEN"
```

```r
data_ml_sieve2 <- data_ml_sieve2[complete.cases(data_ml_sieve2), ]

data_ml_sieve <- data_ml_sieve2[, c("IDEN")] %>%
  left_join(., data_ml_sieve) %>%
  left_join(., NIR_scores)
```

```
## Joining, by = "IDEN"
## Joining, by = "IDEN"
```

```r
data_ml_sieve <- data_ml_sieve[complete.cases(data_ml_sieve), ]
```

Training and testing partitioning

Data partitioning is used to divide data in training (70%) and test data (30%) using 'createDataPartition' function from **caret** package. For each set, data partitionning was made for percentages, slope/intercept. Then, for ilrs. `input` selects columns. Theses values will be compared to predictions.

```r
training <- function(data) {
inTraining <- createDataPartition(data$ilr1, p = .70, list = FALSE)
training_scores <- data[ inTraining,]
input_tr <- as.matrix(dplyr::select(training_scores, c('CLAY', 'SILT', 'SAND', 'ilr1', 'ilr2', 'ilr3'))

return(training_scores)
}

testing <- function(data) {
inTraining <- createDataPartition(data$ilr1, p = .70, list = FALSE)
testing_scores <- data[ -inTraining,]
input_te <- as.matrix(dplyr::select(testing_scores, c('CLAY', 'SILT', 'SAND', 'ilr1', 'ilr2', 'ilr3')))

return(testing_scores)
}

#For comparable items
training_double <- function(data) {
inTraining <- createDataPartition(data$ilr1, p = .70, list = FALSE)
return(inTraining)
}
```

All sets were splitted in training and testing groups.

```r
# Laser 2 min - set1
training_laser <- training(data_ml_laser)
testing_laser <- testing(data_ml_laser)

# Laser 2 min with 2-points 2-h Bouyoucos - set2
training_tot <- training(data_ml_tot)
testing_tot <- testing(data_ml_tot)

# Multi-points 7-h Bouyoucos - set3
inTraining_multi <- training_double(data_ml_multipoint)
training_multipoint <- data_ml_multipoint[ inTraining_multi,]
training_twopoint <- data_ml_twopoint[ inTraining_multi,]

testing_multipoint <- data_ml_multipoint[ -inTraining_multi,]
testing_twopoint <- data_ml_twopoint[ -inTraining_multi,]

# 2-points 2-h Bouyoucos with MIR - set4
inTraining_MIR <- training_double(data_ml_MIR)
training_MIR <- data_ml_MIR[ inTraining_MIR,]
training_NIR <- data_ml_NIR[ inTraining_MIR,]

testing_MIR <- data_ml_MIR[ -inTraining_MIR,]
testing_NIR <- data_ml_NIR[ -inTraining_MIR,]

# 2-points 2-h Bouyoucos with NIR4X - set5
inTraining_NIR4X <- training_double(data_ml_NIR4X)
training_NIR4X <- data_ml_NIR4X[ inTraining_NIR4X,]
training_NIR2X <- data_ml_NIR2X[ inTraining_NIR4X,]

testing_NIR4X <- data_ml_NIR4X[ -inTraining_NIR4X,]
testing_NIR2X <- data_ml_NIR2X[ -inTraining_NIR4X,]

# slope and intercept from Bouyoucos - set6
training_coeff <- training(data_ml_coeff)
testing_coeff <- testing(data_ml_coeff)

# 2-points 2-h Bouyoucos with features - set7
training_feat <- training(data_ml_feat)
testing_feat <- testing(data_ml_feat)

# 2-points 2-h Bouyoucos with sieved sand - set8
inTraining <- createDataPartition(data_ml_sieve$SAND, p = .70, list = FALSE)
training_sieve <- data_ml_sieve[ inTraining,]
testing_sieve <- data_ml_sieve[ -inTraining,]

training_sieve_bouy <- data_ml_sieve2[ inTraining,]
testing_sieve_bouy <- data_ml_sieve2[ -inTraining,]
```

```r
#inTraining_feat <- createDataPartition(data_ml_feat$SAND, p = .70, list = FALSE)
#training_scores_feat <- data_ml_feat[ inTraining_feat,]
#testing_scores_feat <- data_ml_feat[ -inTraining_feat,]

#input_feat_tr <- training_scores_feat %>%
```

```r
#  ungroup() %>%
#  dplyr::select(c('CLAY', 'SILT', 'SAND', 'ilr1', 'ilr2', 'ilr3')) %>%
#  as.matrix()

#input_feat_te <- testing_scores_feat %>%
#  ungroup() %>%
#  dplyr::select(c('CLAY', 'SILT', 'SAND', 'ilr1', 'ilr2', 'ilr3')) %>%
#  as.matrix()
```

Modelisation

Here, we have already tested machine learning models such as svmLinear ou svmLinear2, rqlasso, rqnc, svmLinear3, msanet, bridge, lm, glm, blasso, lasso, spls, foba, ridge, lmStepAIC, rlm, leapBackward, leapSeq, cubist, glmnet_h2o, blassoAveraged, bstLm, rf, gaussprLinear, widekernelpls, kernelpls, lars, lars2, enet, svmRadial, kknn, simpls, gbm, pls, icr, relaxo, leapForward, nnls, pcr, cforest, qrf, rvmlinear, superpc, spikeslab, rfRules from 'caret' package. The 'gbm' seemed to have the best results for ilrs.

ilr

We create a function for ilr modelisation.

```r
# ilr1
modtest_ilr1 <- function(data){

  model <- caret::train(ilr1 ~ .,
                         data = data,
                         method = "gbm",
                         verbose = FALSE)
return(model)
}

#ilr2
modtest_ilr2 <- function(data){

  model <- caret::train(ilr2 ~ .,
                         data = data,
                         method = "gbm",
                         verbose = FALSE)
return(model)
}

#ilr3
modtest_ilr3 <- function(data){

  model <- caret::train(ilr3 ~ .,
                         data = data,
                         method = "gbm",
                         verbose = FALSE)
return(model)
}
```

Percentages

We create a function for percentages modelisation.

```
#SAND
modtest_SAND <- function(data){

  model <- caret::train(SAND ~ .,
                         data = data,
                         method = "gbm",
                         verbose = FALSE)
return(model)
}

#SILT
modtest_SILT <- function(data){

  model <- caret::train(SILT ~ .,
                         data = data,
                         method = "gbm",
                         verbose = FALSE)
return(model)
}

#CLAY
modtest_CLAY <- function(data){

    model <- caret::train(CLAY ~ .,
                         data = data,
                         method = "gbm",
                         verbose = FALSE)
return(model)
}

#Carbon
modtest_C <- function(data){

  model <- caret::train(C_perc ~ .,
                         data = data,
                         method = "gbm",
                         verbose = FALSE)
return(model)
}
```

```
# set1
# character to numeric
training_laser$Method <- recode(training_laser$Method, "Bouyoucos 2-point 2-h" = 1, "Bouyoucos multi-poi
training_laser$Treatment <- recode(training_laser$Treatment, "No peroxide" = 1, "Peroxide" = 2, "2   mir
#sapply(training_laser, function(x) sum(is.na(x)))

##ilr
training_laser_ilr1 <- select(training_laser, -IDEN, -IDEN_Method_treatment, -Method, -Treatment, -Metho
training_laser_ilr2 <- select(training_laser, -IDEN, -IDEN_Method_treatment, -Method, -Treatment, -Metho
training_laser_ilr3 <- select(training_laser, -IDEN, -IDEN_Method_treatment, -Method, -Treatment, -Metho
```

```
mod_set1_ilr1 <- modtest_ilr1(training_laser_ilr1)
mod_set1_ilr2 <- modtest_ilr2(training_laser_ilr2)
mod_set1_ilr3 <- modtest_ilr3(training_laser_ilr3)

##percentages
training_laser_SAND <- select(training_laser, -IDEN, -IDEN_Method_treatment, -Method, -Treatment, -Meth
training_laser_SILT <- select(training_laser, -IDEN, -IDEN_Method_treatment, -Method, -Treatment, -Meth
training_laser_CLAY <- select(training_laser, -IDEN, -IDEN_Method_treatment, -Method, -Treatment, -Meth
training_laser_C <- select(training_laser, -IDEN, -IDEN_Method_treatment, -Method, -Treatment, -Method_

mod_set1_SAND <- modtest_SAND(training_laser_SAND)
mod_set1_SILT <- modtest_SILT(training_laser_SILT)
mod_set1_CLAY <- modtest_CLAY(training_laser_CLAY)
mod_set1_C <- modtest_C(training_laser_C)
```

```
# set2
# character to numeric
training_tot$Method <- recode(training_tot$Method, "Bouyoucos 2-point 2-h" = 1, "Bouyoucos multi-point
training_tot$Treatment <- recode(training_tot$Treatment, "No peroxide" = 1, "Peroxide" = 2, "2   min" =

training_tot_ilr1 <- select(training_tot, -IDEN, -IDEN_Method_treatment, -Method_treatment, -SAND, -SIL
training_tot_ilr2 <- select(training_tot, -IDEN, -IDEN_Method_treatment, -Method_treatment, -SAND, -SIL
training_tot_ilr3 <- select(training_tot, -IDEN, -IDEN_Method_treatment, -Method_treatment, -SAND, -SIL

mod_set2_ilr1 <- modtest_ilr1(training_tot_ilr1)
mod_set2_ilr2 <- modtest_ilr2(training_tot_ilr2)
mod_set2_ilr3 <- modtest_ilr3(training_tot_ilr3)

##percentages
training_tot_SAND <- select(training_tot, -IDEN, -IDEN_Method_treatment, -Method_treatment, -SILT, -CLA
training_tot_SILT <- select(training_tot, -IDEN, -IDEN_Method_treatment, -Method_treatment, -SAND, -CLA
training_tot_CLAY <- select(training_tot, -IDEN, -IDEN_Method_treatment, -Method_treatment, -SAND, -SIL
training_tot_C <- select(training_tot, -IDEN, -IDEN_Method_treatment, -Method_treatment, -SAND, -SILT,

mod_set2_SAND <- modtest_SAND(training_tot_SAND)
mod_set2_SILT <- modtest_SILT(training_tot_SILT)
mod_set2_CLAY <- modtest_CLAY(training_tot_CLAY)
mod_set2_C <- modtest_C(training_tot_C)
```

```
# set3
# character to numeric
training_multipoint$Method <- recode(training_multipoint$Method, "Bouyoucos 2-point 2-h" = 1, "Bouyouco
training_multipoint$Treatment <- recode(training_multipoint$Treatment, "No peroxide" = 1, "Peroxide" =

training_twopoint$Method <- recode(training_twopoint$Method, "Bouyoucos 2-point 2-h" = 1, "Bouyoucos mu
training_twopoint$Treatment <- recode(training_twopoint$Treatment, "No peroxide" = 1, "Peroxide" = 2, "

## ilr
### multi
training_multi_ilr1 <- select(training_multipoint, -IDEN, -IDEN_Method_treatment, -Method, -Treatment,
training_multi_ilr2 <- select(training_multipoint, -IDEN, -IDEN_Method_treatment, -Method, -Treatment,
training_multi_ilr3 <- select(training_multipoint, -IDEN, -IDEN_Method_treatment, -Method, -Treatment,
```

```
mod_set3A_ilr1 <- modtest_ilr1(training_multi_ilr1)
mod_set3A_ilr2 <- modtest_ilr2(training_multi_ilr2)
mod_set3A_ilr3 <- modtest_ilr3(training_multi_ilr3)

### two
training_two_ilr1 <- select(training_twopoint, -IDEN, -IDEN_Method_treatment, -Method, -Treatment, -Met
training_two_ilr2 <- select(training_twopoint, -IDEN, -IDEN_Method_treatment, -Method, -Treatment, -Met
training_two_ilr3 <- select(training_twopoint, -IDEN, -IDEN_Method_treatment, -Method, -Treatment, -Met

mod_set3B_ilr1 <- modtest_ilr1(training_two_ilr1)
mod_set3B_ilr2 <- modtest_ilr2(training_two_ilr2)
mod_set3B_ilr3 <- modtest_ilr3(training_two_ilr3)

## percentages
## multi
training_multi_SAND <- select(training_multipoint, -IDEN, -IDEN_Method_treatment, -Method, -Treatment,
training_multi_SILT <- select(training_multipoint, -IDEN, -IDEN_Method_treatment, -Method, -Treatment,
training_multi_CLAY <- select(training_multipoint, -IDEN, -IDEN_Method_treatment, -Method, -Treatment,
training_multi_C <- select(training_multipoint, -IDEN, -IDEN_Method_treatment, -Method, -Treatment, -Me

mod_set3A_SAND <- modtest_SAND(training_multi_SAND)
mod_set3A_SILT <- modtest_SILT(training_multi_SILT)
mod_set3A_CLAY <- modtest_CLAY(training_multi_CLAY)
mod_set3A_C <- modtest_C(training_multi_C)

## two
training_two_SAND <- select(training_twopoint, -IDEN, -IDEN_Method_treatment, -Method, -Treatment, -Met
training_two_SILT <- select(training_twopoint, -IDEN, -IDEN_Method_treatment, -Method, -Treatment, -Met
training_two_CLAY <- select(training_twopoint, -IDEN, -IDEN_Method_treatment, -Method, -Treatment, -Met
training_two_C <- select(training_twopoint, -IDEN, -IDEN_Method_treatment, -Method, -Treatment, -Method

mod_set3B_SAND <- modtest_SAND(training_two_SAND)
mod_set3B_SILT <- modtest_SILT(training_two_SILT)
mod_set3B_CLAY <- modtest_CLAY(training_two_CLAY)
mod_set3B_C <- modtest_C(training_two_C)
```

```
# set4
# character to numeric
training_MIR$Method <- recode(training_MIR$Method, "Bouyoucos 2-point 2-h" = 1, "Bouyoucos multi-point
training_MIR$Treatment <- recode(training_MIR$Treatment, "No peroxide" = 1, "Peroxide" = 2, "2   min" =

# character to numeric
training_NIR$Method <- recode(training_NIR$Method, "Bouyoucos 2-point 2-h" = 1, "Bouyoucos multi-point
training_NIR$Treatment <- recode(training_NIR$Treatment, "No peroxide" = 1, "Peroxide" = 2, "2   min" =

## ilr
### MIR
training_MIR_ilr1 <- select(training_MIR, -IDEN, -IDEN_Method_treatment, -Method, -Treatment, -Method_t
training_MIR_ilr2 <- select(training_MIR, -IDEN, -IDEN_Method_treatment, -Method, -Treatment, -Method_t
training_MIR_ilr3 <- select(training_MIR, -IDEN, -IDEN_Method_treatment, -Method, -Treatment, -Method_t

mod_set4A_ilr1 <- modtest_ilr1(training_MIR_ilr1)
mod_set4A_ilr2 <- modtest_ilr2(training_MIR_ilr2)
```

```r
mod_set4A_ilr3 <- modtest_ilr3(training_MIR_ilr3)

### NIR
training_NIR_ilr1 <- select(training_NIR, -IDEN, -IDEN_Method_treatment, -Method, -Treatment, -Method_tr
training_NIR_ilr2 <- select(training_NIR, -IDEN, -IDEN_Method_treatment, -Method, -Treatment, -Method_tr
training_NIR_ilr3 <- select(training_NIR, -IDEN, -IDEN_Method_treatment, -Method, -Treatment, -Method_tr

mod_set4B_ilr1 <- modtest_ilr1(training_NIR_ilr1)
mod_set4B_ilr2 <- modtest_ilr2(training_NIR_ilr2)
mod_set4B_ilr3 <- modtest_ilr3(training_NIR_ilr3)

## percentages
## MIR
training_MIR_SAND <- select(training_MIR, -IDEN, -IDEN_Method_treatment, -Method, -Treatment, -Method_tr
training_MIR_SILT <- select(training_MIR, -IDEN, -IDEN_Method_treatment, -Method, -Treatment, -Method_tr
training_MIR_CLAY <- select(training_MIR, -IDEN, -IDEN_Method_treatment, -Method, -Treatment, -Method_tr
training_MIR_C <- select(training_MIR, -IDEN, -IDEN_Method_treatment, -Method, -Treatment, -Method_treat

mod_set4A_SAND <- modtest_SAND(training_MIR_SAND)
mod_set4A_SILT <- modtest_SILT(training_MIR_SILT)
mod_set4A_CLAY <- modtest_CLAY(training_MIR_CLAY)
mod_set4A_C <- modtest_C(training_MIR_C)

## NIR
training_NIR_SAND <- select(training_NIR, -IDEN, -IDEN_Method_treatment, -Method, -Treatment, -Method_tr
training_NIR_SILT <- select(training_NIR, -IDEN, -IDEN_Method_treatment, -Method, -Treatment, -Method_tr
training_NIR_CLAY <- select(training_NIR, -IDEN, -IDEN_Method_treatment, -Method, -Treatment, -Method_tr
training_NIR_C <- select(training_NIR, -IDEN, -IDEN_Method_treatment, -Method, -Treatment, -Method_treat

mod_set4B_SAND <- modtest_SAND(training_NIR_SAND)
mod_set4B_SILT <- modtest_SILT(training_NIR_SILT)
mod_set4B_CLAY <- modtest_CLAY(training_NIR_CLAY)
mod_set4B_C <- modtest_C(training_NIR_C)
```

```r
# set5
# character to numeric
training_NIR4X$Method <- recode(training_NIR4X$Method, "Bouyoucos 2-point 2-h" = 1, "Bouyoucos multi-poi
training_NIR4X$Treatment <- recode(training_NIR4X$Treatment, "No peroxide" = 1, "Peroxide" = 2, "2   min

training_NIR2X$Method <- recode(training_NIR2X$Method, "Bouyoucos 2-point 2-h" = 1, "Bouyoucos multi-poi
training_NIR2X$Treatment <- recode(training_NIR2X$Treatment, "No peroxide" = 1, "Peroxide" = 2, "2   min

## ilr
### NIR4X
training_NIR4X_ilr1 <- select(training_NIR4X, -IDEN, -IDEN_Method_treatment, -Method, -Treatment, -Metho
training_NIR4X_ilr2 <- select(training_NIR4X, -IDEN, -IDEN_Method_treatment, -Method, -Treatment,  -Meth
training_NIR4X_ilr3 <- select(training_NIR4X, -IDEN, -IDEN_Method_treatment, -Method, -Treatment,  -Meth

mod_set5A_ilr1 <- modtest_ilr1(training_NIR4X_ilr1)
mod_set5A_ilr2 <- modtest_ilr2(training_NIR4X_ilr2)
mod_set5A_ilr3 <- modtest_ilr3(training_NIR4X_ilr3)

### NIR2X
```

```
training_NIR2X_ilr1 <- select(training_NIR2X, -IDEN, -IDEN_Method_treatment, -Method, -Treatment,  -Met
training_NIR2X_ilr2 <- select(training_NIR2X, -IDEN, -IDEN_Method_treatment, -Method, -Treatment,  -Met
training_NIR2X_ilr3 <- select(training_NIR2X, -IDEN, -IDEN_Method_treatment, -Method, -Treatment,  -Met

mod_set5B_ilr1 <- modtest_ilr1(training_NIR2X_ilr1)
mod_set5B_ilr2 <- modtest_ilr2(training_NIR2X_ilr2)
mod_set5B_ilr3 <- modtest_ilr3(training_NIR2X_ilr3)

## percentages
## NIR4X
training_NIR4X_SAND <- select(training_NIR4X, -IDEN, -IDEN_Method_treatment, -Method, -Treatment,  -Met
training_NIR4X_SILT <- select(training_NIR4X, -IDEN, -IDEN_Method_treatment, -Method, -Treatment,  -Met
training_NIR4X_CLAY <- select(training_NIR4X, -IDEN, -IDEN_Method_treatment, -Method, -Treatment,  -Met
training_NIR4X_C <- select(training_NIR4X, -IDEN, -IDEN_Method_treatment, -Method, -Treatment,  -Method_

mod_set5A_SAND <- modtest_SAND(training_NIR4X_SAND)
mod_set5A_SILT <- modtest_SILT(training_NIR4X_SILT)
mod_set5A_CLAY <- modtest_CLAY(training_NIR4X_CLAY)
mod_set5A_C <- modtest_C(training_NIR4X_C)

## NIR2X
training_NIR2X_SAND <- select(training_NIR2X, -IDEN, -IDEN_Method_treatment, -Method, -Treatment,  -Met
training_NIR2X_SILT <- select(training_NIR2X, -IDEN, -IDEN_Method_treatment, -Method, -Treatment,  -Met
training_NIR2X_CLAY <- select(training_NIR2X, -IDEN, -IDEN_Method_treatment, -Method, -Treatment,  -Met
training_NIR2X_C <- select(training_NIR2X, -IDEN, -IDEN_Method_treatment, -Method, -Treatment,  -Method_

mod_set5B_SAND <- modtest_SAND(training_NIR2X_SAND)
mod_set5B_SILT <- modtest_SILT(training_NIR2X_SILT)
mod_set5B_CLAY <- modtest_CLAY(training_NIR2X_CLAY)
mod_set5B_C <- modtest_C(training_NIR2X_C)
```

```
#Set 6
training_coeff$Method <- recode(training_coeff$Method, "Bouyoucos 2-point 2-h" = 1, "Bouyoucos multi-po
training_coeff$Treatment <- recode(training_coeff$Treatment, "No peroxide" = 1, "Peroxide" = 2, "2   mi

## Slope and intercept
### Intercept
training_coeff_intercept <- select(training_coeff, -IDEN, -IDEN_Method_treatment, -Method, -Method_trea
mod_set6_intercept = caret::train(Intercept ~ .,
                          data = training_coeff_intercept,
                          method = "gbm",
                         verbose = FALSE)

### Slope
training_coeff_slope <- select(training_coeff, -IDEN, -IDEN_Method_treatment, -Method, -Method_treatmen
mod_set6_slope = caret::train(Slope ~ .,
                          data = training_coeff_slope,
                          method = "gbm",
                         verbose = FALSE)
### Carbon
training_coeff_carbon <- select(training_coeff, -IDEN, -IDEN_Method_treatment, -Method, -Method_treatme
mod_set6_carbon = caret::train(C_perc ~ .,
                          data = training_coeff_carbon,
```

```
                          method = "gbm",
                         verbose = FALSE)

## ilr
training_coeff_ilr1 <- select(training_coeff, -IDEN, -IDEN_Method_treatment, -Method, -Method_treatment
training_coeff_ilr2 <- select(training_coeff, -IDEN, -IDEN_Method_treatment, -Method, -Method_treatment
training_coeff_ilr3 <- select(training_coeff, -IDEN, -IDEN_Method_treatment, -Method, -Method_treatment

mod_set6A_ilr1 <- modtest_ilr1(training_coeff_ilr1)
mod_set6A_ilr2 <- modtest_ilr2(training_coeff_ilr2)
mod_set6A_ilr3 <- modtest_ilr3(training_coeff_ilr3)

## percentages
training_coeff_SAND <- select(training_coeff, -IDEN, -IDEN_Method_treatment, -Method, -Method_treatment
training_coeff_SILT <- select(training_coeff, -IDEN, -IDEN_Method_treatment, -Method, -Method_treatment
training_coeff_CLAY <- select(training_coeff, -IDEN, -IDEN_Method_treatment, -Method, -Method_treatment
training_coeff_C <- select(training_coeff, -IDEN, -IDEN_Method_treatment, -Method, -Method_treatment, -

mod_set6B_SAND <- modtest_SAND(training_coeff_SAND)
mod_set6B_SILT <- modtest_SILT(training_coeff_SILT)
mod_set6B_CLAY <- modtest_CLAY(training_coeff_CLAY)
mod_set6B_C <- modtest_C(training_coeff_C)
```

```
# Set 7
training_feat$Method <- recode(training_feat$Method, "Bouyoucos 2-point 2-h" = 1, "Bouyoucos multi-poin
training_feat$Treatment <- recode(training_feat$Treatment, "No peroxide" = 1, "Peroxide" = 2, "2   min"

## All features
### ilr
training_feat_all_ilr1 <- select(training_feat, -IDEN, -IDEN_Method_treatment, -Method, -Treatment, -Me
training_feat_all_ilr2 <- select(training_feat, -IDEN, -IDEN_Method_treatment, -Method, -Treatment, -Me
training_feat_all_ilr3 <- select(training_feat, -IDEN, -IDEN_Method_treatment, -Method, -Treatment, -Me

mod_set7all_ilr1 <- modtest_ilr1(training_feat_all_ilr1)
mod_set7all_ilr2 <- modtest_ilr2(training_feat_all_ilr2)
mod_set7all_ilr3 <- modtest_ilr3(training_feat_all_ilr3)

## Carbon
### ilr
training_feat_c_ilr1 <- select(training_feat, -IDEN, -IDEN_Method_treatment, -Method, -Treatment, -Meth
training_feat_c_ilr2 <- select(training_feat, -IDEN, -IDEN_Method_treatment, -Method, -Treatment, -Meth
training_feat_c_ilr3 <- select(training_feat, -IDEN, -IDEN_Method_treatment, -Method, -Treatment, -Meth

mod_set7c_ilr1 <- modtest_ilr1(training_feat_c_ilr1)
mod_set7c_ilr2 <- modtest_ilr2(training_feat_c_ilr2)
mod_set7c_ilr3 <- modtest_ilr3(training_feat_c_ilr3)

## Density
### ilr
training_feat_d_ilr1 <- select(training_feat, -IDEN, -IDEN_Method_treatment, -Method, -Treatment, -Meth
training_feat_d_ilr2 <- select(training_feat, -IDEN, -IDEN_Method_treatment, -Method, -Treatment, -Meth
training_feat_d_ilr3 <- select(training_feat, -IDEN, -IDEN_Method_treatment, -Method, -Treatment, -Meth
```

```r
mod_set7d_ilr1 <- modtest_ilr1(training_feat_d_ilr1)
mod_set7d_ilr2 <- modtest_ilr2(training_feat_d_ilr2)
mod_set7d_ilr3 <- modtest_ilr3(training_feat_d_ilr3)

## pH
### ilr
training_feat_ph_ilr1 <- select(training_feat, -IDEN, -IDEN_Method_treatment, -Method, -Treatment, -Met
training_feat_ph_ilr2 <- select(training_feat, -IDEN, -IDEN_Method_treatment, -Method, -Treatment, -Met
training_feat_ph_ilr3 <- select(training_feat, -IDEN, -IDEN_Method_treatment, -Method, -Treatment, -Met

mod_set7ph_ilr1 <- modtest_ilr1(training_feat_ph_ilr1)
mod_set7ph_ilr2 <- modtest_ilr2(training_feat_ph_ilr2)
mod_set7ph_ilr3 <- modtest_ilr3(training_feat_ph_ilr3)

## Colors
### ilr
training_feat_rgb_ilr1 <- select(training_feat, -IDEN, -IDEN_Method_treatment, -Method, -Treatment, -Me
training_feat_rgb_ilr2 <- select(training_feat, -IDEN, -IDEN_Method_treatment, -Method, -Treatment, -Me
training_feat_rgb_ilr3 <- select(training_feat, -IDEN, -IDEN_Method_treatment, -Method, -Treatment, -Me

mod_set7rgb_ilr1 <- modtest_ilr1(training_feat_rgb_ilr1)
mod_set7rgb_ilr2 <- modtest_ilr2(training_feat_rgb_ilr2)
mod_set7rgb_ilr3 <- modtest_ilr3(training_feat_rgb_ilr3)

##Oxalate
### ilr
training_feat_ox_ilr1 <- select(training_feat, -IDEN, -IDEN_Method_treatment, -Method, -Treatment, -Met
training_feat_ox_ilr2 <- select(training_feat, -IDEN, -IDEN_Method_treatment, -Method, -Treatment, -Met
training_feat_ox_ilr3 <- select(training_feat, -IDEN, -IDEN_Method_treatment, -Method, -Treatment, -Met

mod_set7ox_ilr1 <- modtest_ilr1(training_feat_ox_ilr1)
mod_set7ox_ilr2 <- modtest_ilr2(training_feat_ox_ilr2)
mod_set7ox_ilr3 <- modtest_ilr3(training_feat_ox_ilr3)

## M3
### ilr
training_feat_m3_ilr1 <- select(training_feat, -IDEN, -IDEN_Method_treatment, -Method, -Treatment, -Met
training_feat_m3_ilr2 <- select(training_feat, -IDEN, -IDEN_Method_treatment, -Method, -Treatment, -Met
training_feat_m3_ilr3 <- select(training_feat, -IDEN, -IDEN_Method_treatment, -Method, -Treatment, -Met

mod_set7m3_ilr1 <- modtest_ilr1(training_feat_m3_ilr1)
mod_set7m3_ilr2 <- modtest_ilr2(training_feat_m3_ilr2)
mod_set7m3_ilr3 <- modtest_ilr3(training_feat_m3_ilr3)

## No features
### ilr
training_feat_no_ilr1 <- select(training_feat, -IDEN, -IDEN_Method_treatment, -Method, -Treatment, -Met
training_feat_no_ilr2 <- select(training_feat, -IDEN, -IDEN_Method_treatment, -Method, -Treatment, -Met
training_feat_no_ilr3 <- select(training_feat, -IDEN, -IDEN_Method_treatment, -Method, -Treatment, -Met

mod_set7no_ilr1 <- modtest_ilr1(training_feat_no_ilr1)
mod_set7no_ilr2 <- modtest_ilr2(training_feat_no_ilr2)
mod_set7no_ilr3 <- modtest_ilr3(training_feat_no_ilr3)
```

```r
# Set 8
training_sieve$Method <- recode(training_sieve$Method, "Bouyoucos 2-point 2-h" = 1, "Bouyoucos multi-poi
training_sieve$Treatment <- recode(training_sieve$Treatment, "No peroxide" = 1, "Peroxide" = 2, "2   mi

training_sieve_bouy$Treatment <- recode(training_sieve_bouy$Treatment, "No peroxide" = 1, "Peroxide" = 

## Sieving
training_sieve_SAND <- select(training_sieve, -IDEN, -IDEN_Method_treatment, -Method, -Treatment, -Meth
mod_set8_SAND <- modtest_SAND(training_sieve_SAND)

## Bouyoucos 2-point 2-h
training_sieve_bouy <- select(training_sieve_bouy, -IDEN, -IDEN_Method_treatment, -Method, -Method_trea
mod_set8bouy_SAND <- modtest_SAND(training_sieve_bouy)
```

Predictions

We create a function for predictions from model.

```r
prediction <- function(model, testing, original) {
  testing$Method <- recode(testing$Method, "Bouyoucos 2-point 2-h" = 1, "Bouyoucos multi-point 7-h" = 2
  testing$Treatment <- recode(testing$Treatment, "No peroxide" = 1, "Peroxide" = 2, "2   min" = 3)

  pred <- as.data.frame(predict(model, testing))
  testing$Method <- recode(testing$Method, "1" = "Bouyoucos 2-point 2-h", "2" = "Bouyoucos multi-point 
  testing$Method <- as.character(testing$Method)
  testing$Treatment <- recode(testing$Treatment, "1" = "No peroxide", "2" = "Peroxide", "3" = "2   min"
  testing$Treatment <- as.character(testing$Treatment)

  result <- pred %>%
    cbind2(testing[, c("IDEN", "IDEN_Method_treatment", "Method", "Treatment", "Method_treatment")]) %>
    left_join(., original[, c("IDEN", "IDEN_Method_treatment", "Method", "Treatment", "Method_treatment

  return(result)
}
```

We predict results for testing samples.

```r
# Set1
## ilr
pred_set1_ilr1 <- prediction(mod_set1_ilr1, testing_laser, data_ml_laser) %>% dplyr::rename(ilr1_IR = "
```

```
## Joining, by = c("IDEN", "IDEN_Method_treatment", "Method", "Treatment", "Method_treatment")
```

```r
pred_set1_ilr2 <- prediction(mod_set1_ilr2, testing_laser, data_ml_laser) %>% dplyr::rename(ilr2_IR = "
```

```
## Joining, by = c("IDEN", "IDEN_Method_treatment", "Method", "Treatment", "Method_treatment")
```

```r
pred_set1_ilr3 <- prediction(mod_set1_ilr3, testing_laser, data_ml_laser) %>% dplyr::rename(ilr3_IR = "
```

```
## Joining, by = c("IDEN", "IDEN_Method_treatment", "Method", "Treatment", "Method_treatment")
```

```r
#is.na(pred_set1_ilr1)

# percentages
pred_set1_SAND <- prediction(mod_set1_SAND, testing_laser, data_ml_laser) %>% dplyr::rename(SAND_IR = "
```

```
## Joining, by = c("IDEN", "IDEN_Method_treatment", "Method", "Treatment", "Method_treatment")
```

```r
pred_set1_SILT <- prediction(mod_set1_SILT, testing_laser, data_ml_laser) %>% dplyr::rename(SILT_IR = "
```

```
## Joining, by = c("IDEN", "IDEN_Method_treatment", "Method", "Treatment", "Method_treatment")
```

```r
pred_set1_CLAY <- prediction(mod_set1_CLAY, testing_laser, data_ml_laser) %>% dplyr::rename(CLAY_IR = "
```

```
## Joining, by = c("IDEN", "IDEN_Method_treatment", "Method", "Treatment", "Method_treatment")
```

```r
pred_set1_C <- prediction(mod_set1_C, testing_laser, data_ml_laser) %>% dplyr::rename(C_IR = "predict(m
```

```
## Joining, by = c("IDEN", "IDEN_Method_treatment", "Method", "Treatment", "Method_treatment")
```

```r
#is.na(pred_set1_SAND)
```

```r
# Set2
## ilr
pred_set2_ilr1 <- prediction(mod_set2_ilr1, testing_tot, data_ml_tot) %>% dplyr::rename(ilr1_IR = "pred
```

```
## Joining, by = c("IDEN", "IDEN_Method_treatment", "Method", "Treatment", "Method_treatment")
```

```r
pred_set2_ilr2 <- prediction(mod_set2_ilr2, testing_tot, data_ml_tot) %>% dplyr::rename(ilr2_IR = "pred
```

```
## Joining, by = c("IDEN", "IDEN_Method_treatment", "Method", "Treatment", "Method_treatment")
```

```r
pred_set2_ilr3 <- prediction(mod_set2_ilr3, testing_tot, data_ml_tot) %>% dplyr::rename(ilr3_IR = "pred
```

```
## Joining, by = c("IDEN", "IDEN_Method_treatment", "Method", "Treatment", "Method_treatment")
```

```r
#percentages
pred_set2_SAND <- prediction(mod_set2_SAND, testing_tot, data_ml_tot) %>% dplyr::rename(SAND_IR = "pred
```

```
## Joining, by = c("IDEN", "IDEN_Method_treatment", "Method", "Treatment", "Method_treatment")
```

```r
pred_set2_SILT <- prediction(mod_set2_SILT, testing_tot, data_ml_tot) %>% dplyr::rename(SILT_IR = "pred
```

```
## Joining, by = c("IDEN", "IDEN_Method_treatment", "Method", "Treatment", "Method_treatment")
```

```r
pred_set2_CLAY <- prediction(mod_set2_CLAY, testing_tot, data_ml_tot) %>% dplyr::rename(CLAY_IR = "predi
```

```
## Joining, by = c("IDEN", "IDEN_Method_treatment", "Method", "Treatment", "Method_treatment")
```

```r
pred_set2_C <- prediction(mod_set2_C, testing_tot, data_ml_tot) %>% dplyr::rename(C_IR = "predict(model
```

```
## Joining, by = c("IDEN", "IDEN_Method_treatment", "Method", "Treatment", "Method_treatment")
```

```r
# Set3
## multipoint
### ilr
pred_set3A_ilr1 <- prediction(mod_set3A_ilr1, testing_multipoint, data_ml_multipoint) %>% dplyr::rename
```

```
## Joining, by = c("IDEN", "IDEN_Method_treatment", "Method", "Treatment", "Method_treatment")
```

```r
pred_set3A_ilr2 <- prediction(mod_set3A_ilr2, testing_multipoint, data_ml_multipoint) %>% dplyr::rename
```

```
## Joining, by = c("IDEN", "IDEN_Method_treatment", "Method", "Treatment", "Method_treatment")
```

```r
pred_set3A_ilr3 <- prediction(mod_set3A_ilr3, testing_multipoint, data_ml_multipoint) %>% dplyr::rename
```

```
## Joining, by = c("IDEN", "IDEN_Method_treatment", "Method", "Treatment", "Method_treatment")
```

```r
### percentages
pred_set3A_SAND <- prediction(mod_set3A_SAND, testing_multipoint, data_ml_multipoint) %>% dplyr::rename
```

```
## Joining, by = c("IDEN", "IDEN_Method_treatment", "Method", "Treatment", "Method_treatment")
```

```r
pred_set3A_SILT <- prediction(mod_set3A_SILT, testing_multipoint, data_ml_multipoint) %>% dplyr::rename
```

```
## Joining, by = c("IDEN", "IDEN_Method_treatment", "Method", "Treatment", "Method_treatment")
```

```r
pred_set3A_CLAY <- prediction(mod_set3A_CLAY, testing_multipoint, data_ml_multipoint) %>% dplyr::rename
```

```
## Joining, by = c("IDEN", "IDEN_Method_treatment", "Method", "Treatment", "Method_treatment")
```

```r
pred_set3A_C <- prediction(mod_set3A_C, testing_multipoint, data_ml_multipoint) %>% dplyr::rename(C_IR =
```

```
## Joining, by = c("IDEN", "IDEN_Method_treatment", "Method", "Treatment", "Method_treatment")
```

```r
## twopoint
### ilr
pred_set3B_ilr1 <- prediction(mod_set3B_ilr1, testing_twopoint, data_ml_twopoint) %>% dplyr::rename(ilr
```

```
## Joining, by = c("IDEN", "IDEN_Method_treatment", "Method", "Treatment", "Method_treatment")
```

```
pred_set3B_ilr2 <- prediction(mod_set3B_ilr2, testing_twopoint, data_ml_twopoint) %>% dplyr::rename(ilr
```

```
## Joining, by = c("IDEN", "IDEN_Method_treatment", "Method", "Treatment", "Method_treatment")
```

```
pred_set3B_ilr3 <- prediction(mod_set3B_ilr3, testing_twopoint, data_ml_twopoint) %>% dplyr::rename(ilr
```

```
## Joining, by = c("IDEN", "IDEN_Method_treatment", "Method", "Treatment", "Method_treatment")
```

```
### percentages
pred_set3B_SAND <- prediction(mod_set3B_SAND, testing_twopoint, data_ml_twopoint) %>% dplyr::rename(SAN
```

```
## Joining, by = c("IDEN", "IDEN_Method_treatment", "Method", "Treatment", "Method_treatment")
```

```
pred_set3B_SILT <- prediction(mod_set3B_SILT, testing_twopoint, data_ml_twopoint) %>% dplyr::rename(SIL
```

```
## Joining, by = c("IDEN", "IDEN_Method_treatment", "Method", "Treatment", "Method_treatment")
```

```
pred_set3B_CLAY <- prediction(mod_set3B_CLAY, testing_twopoint, data_ml_twopoint) %>% dplyr::rename(CLA
```

```
## Joining, by = c("IDEN", "IDEN_Method_treatment", "Method", "Treatment", "Method_treatment")
```

```
pred_set3B_C <- prediction(mod_set3B_C, testing_twopoint, data_ml_twopoint) %>% dplyr::rename(C_IR = "p
```

```
## Joining, by = c("IDEN", "IDEN_Method_treatment", "Method", "Treatment", "Method_treatment")
```

```
# Set4
## MIR
### ilr
pred_set4A_ilr1 <- prediction(mod_set4A_ilr1, testing_MIR, data_ml_MIR) %>% dplyr::rename(ilr1_IR = "pr
```

```
## Joining, by = c("IDEN", "IDEN_Method_treatment", "Method", "Treatment", "Method_treatment")
```

```
pred_set4A_ilr2 <- prediction(mod_set4A_ilr2, testing_MIR, data_ml_MIR) %>% dplyr::rename(ilr2_IR = "pr
```

```
## Joining, by = c("IDEN", "IDEN_Method_treatment", "Method", "Treatment", "Method_treatment")
```

```
pred_set4A_ilr3 <- prediction(mod_set4A_ilr3, testing_MIR, data_ml_MIR) %>% dplyr::rename(ilr3_IR = "pr
```

```
## Joining, by = c("IDEN", "IDEN_Method_treatment", "Method", "Treatment", "Method_treatment")
```

```
### percentages
pred_set4A_SAND <- prediction(mod_set4A_SAND, testing_MIR, data_ml_MIR) %>% dplyr::rename(SAND_IR = "pr
```

```
## Joining, by = c("IDEN", "IDEN_Method_treatment", "Method", "Treatment", "Method_treatment")
```

```
pred_set4A_SILT <- prediction(mod_set4A_SILT, testing_MIR, data_ml_MIR) %>% dplyr::rename(SILT_IR = "pr
```

```
## Joining, by = c("IDEN", "IDEN_Method_treatment", "Method", "Treatment", "Method_treatment")
```

```
pred_set4A_CLAY <- prediction(mod_set4A_CLAY, testing_MIR, data_ml_MIR) %>% dplyr::rename(CLAY_IR = "pr
```

```
## Joining, by = c("IDEN", "IDEN_Method_treatment", "Method", "Treatment", "Method_treatment")
```

```
pred_set4A_C <- prediction(mod_set4A_C, testing_MIR, data_ml_MIR) %>% dplyr::rename(C_IR = "predict(mod
```

```
## Joining, by = c("IDEN", "IDEN_Method_treatment", "Method", "Treatment", "Method_treatment")
```

```
## NIR
### ilr
pred_set4B_ilr1 <- prediction(mod_set4B_ilr1, testing_NIR, data_ml_NIR) %>% dplyr::rename(ilr1_IR = "pr
```

```
## Joining, by = c("IDEN", "IDEN_Method_treatment", "Method", "Treatment", "Method_treatment")
```

```
pred_set4B_ilr2 <- prediction(mod_set4B_ilr2, testing_NIR, data_ml_NIR) %>% dplyr::rename(ilr2_IR = "pr
```

```
## Joining, by = c("IDEN", "IDEN_Method_treatment", "Method", "Treatment", "Method_treatment")
```

```
pred_set4B_ilr3 <- prediction(mod_set4B_ilr3, testing_NIR, data_ml_NIR) %>% dplyr::rename(ilr3_IR = "pr
```

```
## Joining, by = c("IDEN", "IDEN_Method_treatment", "Method", "Treatment", "Method_treatment")
```

```
### percentages
pred_set4B_SAND <- prediction(mod_set4B_SAND, testing_NIR, data_ml_NIR) %>% dplyr::rename(SAND_IR = "pr
```

```
## Joining, by = c("IDEN", "IDEN_Method_treatment", "Method", "Treatment", "Method_treatment")
```

```
pred_set4B_SILT <- prediction(mod_set4B_SILT, testing_NIR, data_ml_NIR) %>% dplyr::rename(SILT_IR = "pr
```

```
## Joining, by = c("IDEN", "IDEN_Method_treatment", "Method", "Treatment", "Method_treatment")
```

```
pred_set4B_CLAY <- prediction(mod_set4B_CLAY, testing_NIR, data_ml_NIR) %>% dplyr::rename(CLAY_IR = "pr
```

```
## Joining, by = c("IDEN", "IDEN_Method_treatment", "Method", "Treatment", "Method_treatment")
```

```
pred_set4B_C <- prediction(mod_set4B_C, testing_NIR, data_ml_NIR) %>% dplyr::rename(C_IR = "predict(mod
```

```
## Joining, by = c("IDEN", "IDEN_Method_treatment", "Method", "Treatment", "Method_treatment")
```

```r
# Set5
## NIR4X
### ilr
pred_set5A_ilr1 <- prediction(mod_set5A_ilr1, testing_NIR4X, data_ml_NIR4X) %>% dplyr::rename(ilr1_IR =
```

```
## Joining, by = c("IDEN", "IDEN_Method_treatment", "Method", "Treatment", "Method_treatment")
```

```r
pred_set5A_ilr2 <- prediction(mod_set5A_ilr2, testing_NIR4X, data_ml_NIR4X) %>% dplyr::rename(ilr2_IR =
```

```
## Joining, by = c("IDEN", "IDEN_Method_treatment", "Method", "Treatment", "Method_treatment")
```

```r
pred_set5A_ilr3 <- prediction(mod_set5A_ilr3, testing_NIR4X, data_ml_NIR4X) %>% dplyr::rename(ilr3_IR =
```

```
## Joining, by = c("IDEN", "IDEN_Method_treatment", "Method", "Treatment", "Method_treatment")
```

```r
### percentages
pred_set5A_SAND <- prediction(mod_set5A_SAND, testing_NIR4X, data_ml_NIR4X) %>% dplyr::rename(SAND_IR =
```

```
## Joining, by = c("IDEN", "IDEN_Method_treatment", "Method", "Treatment", "Method_treatment")
```

```r
pred_set5A_SILT <- prediction(mod_set5A_SILT, testing_NIR4X, data_ml_NIR4X) %>% dplyr::rename(SILT_IR =
```

```
## Joining, by = c("IDEN", "IDEN_Method_treatment", "Method", "Treatment", "Method_treatment")
```

```r
pred_set5A_CLAY <- prediction(mod_set5A_CLAY, testing_NIR4X, data_ml_NIR4X) %>% dplyr::rename(CLAY_IR =
```

```
## Joining, by = c("IDEN", "IDEN_Method_treatment", "Method", "Treatment", "Method_treatment")
```

```r
pred_set5A_C <- prediction(mod_set5A_C, testing_NIR4X, data_ml_NIR4X) %>% dplyr::rename(C_IR = "predict
```

```
## Joining, by = c("IDEN", "IDEN_Method_treatment", "Method", "Treatment", "Method_treatment")
```

```r
## NIR2X
### ilr
pred_set5B_ilr1 <- prediction(mod_set5B_ilr1, testing_NIR2X, data_ml_NIR2X) %>% dplyr::rename(ilr1_IR =
```

```
## Joining, by = c("IDEN", "IDEN_Method_treatment", "Method", "Treatment", "Method_treatment")
```

```r
pred_set5B_ilr2 <- prediction(mod_set5B_ilr2, testing_NIR2X, data_ml_NIR2X) %>% dplyr::rename(ilr2_IR =
```

```
## Joining, by = c("IDEN", "IDEN_Method_treatment", "Method", "Treatment", "Method_treatment")
```

```r
pred_set5B_ilr3 <- prediction(mod_set5B_ilr3, testing_NIR2X, data_ml_NIR2X) %>% dplyr::rename(ilr3_IR =
```

```
## Joining, by = c("IDEN", "IDEN_Method_treatment", "Method", "Treatment", "Method_treatment")
```

```r
### percentages
pred_set5B_SAND <- prediction(mod_set5B_SAND, testing_NIR2X, data_ml_NIR2X) %>% dplyr::rename(SAND_IR =
```

```
## Joining, by = c("IDEN", "IDEN_Method_treatment", "Method", "Treatment", "Method_treatment")
```

```r
pred_set5B_SILT <- prediction(mod_set5B_SILT, testing_NIR2X, data_ml_NIR2X) %>% dplyr::rename(SILT_IR =
```

```
## Joining, by = c("IDEN", "IDEN_Method_treatment", "Method", "Treatment", "Method_treatment")
```

```r
pred_set5B_CLAY <- prediction(mod_set5B_CLAY, testing_NIR2X, data_ml_NIR2X) %>% dplyr::rename(CLAY_IR =
```

```
## Joining, by = c("IDEN", "IDEN_Method_treatment", "Method", "Treatment", "Method_treatment")
```

```r
pred_set5B_C <- prediction(mod_set5B_C, testing_NIR2X, data_ml_NIR2X) %>% dplyr::rename(C_IR = "predict
```

```
## Joining, by = c("IDEN", "IDEN_Method_treatment", "Method", "Treatment", "Method_treatment")
```

```r
# Set6
## Slope and intercept
pred_set6_slope <- prediction(mod_set6_slope, testing_coeff, data_ml_coeff) %>%
  left_join(., data_ml_coeff[,c("IDEN_Method_treatment", "Slope")]) %>%
  dplyr::rename(Slope_coeff_NIR = "predict(model, testing)")
```

```
## Joining, by = c("IDEN", "IDEN_Method_treatment", "Method", "Treatment", "Method_treatment")
```

```
## Joining, by = "IDEN_Method_treatment"
```

```r
pred_set6_intercept <- prediction(mod_set6_intercept, testing_coeff, data_ml_coeff) %>%
  left_join(., data_ml_coeff[,c("IDEN_Method_treatment", "Intercept")]) %>%
  dplyr::rename(Intercept_coeff_NIR = "predict(model, testing)")
```

```
## Joining, by = c("IDEN", "IDEN_Method_treatment", "Method", "Treatment", "Method_treatment")
## Joining, by = "IDEN_Method_treatment"
```

```r
pred_set6_carbon <- prediction(mod_set6_carbon, testing_coeff, data_ml_coeff) %>%
  left_join(., data_ml_coeff[,c("IDEN_Method_treatment", "Intercept")]) %>%
  dplyr::rename(Carbon_coeff_NIR = "predict(model, testing)")
```

```
## Joining, by = c("IDEN", "IDEN_Method_treatment", "Method", "Treatment", "Method_treatment")
## Joining, by = "IDEN_Method_treatment"
```

```r
## ilr
pred_set6_ilr1 <- prediction(mod_set6A_ilr1, testing_coeff, data_ml_coeff) %>% dplyr::rename(ilr1_IR =
```

```
## Joining, by = c("IDEN", "IDEN_Method_treatment", "Method", "Treatment", "Method_treatment")
```

```r
pred_set6_ilr2 <- prediction(mod_set6A_ilr2, testing_coeff, data_ml_coeff) %>% dplyr::rename(ilr2_IR =
```

```
## Joining, by = c("IDEN", "IDEN_Method_treatment", "Method", "Treatment", "Method_treatment")
```

```r
pred_set6_ilr3 <- prediction(mod_set6A_ilr3, testing_coeff, data_ml_coeff) %>% dplyr::rename(ilr3_IR =
```

```
## Joining, by = c("IDEN", "IDEN_Method_treatment", "Method", "Treatment", "Method_treatment")
```

```r
## percentages
pred_set6_SAND <- prediction(mod_set6B_SAND, testing_coeff, data_ml_coeff) %>% dplyr::rename(SAND_IR =
```

```
## Joining, by = c("IDEN", "IDEN_Method_treatment", "Method", "Treatment", "Method_treatment")
```

```r
pred_set6_SILT <- prediction(mod_set6B_SILT, testing_coeff, data_ml_coeff) %>% dplyr::rename(SILT_IR =
```

```
## Joining, by = c("IDEN", "IDEN_Method_treatment", "Method", "Treatment", "Method_treatment")
```

```r
pred_set6_CLAY <- prediction(mod_set6B_CLAY, testing_coeff, data_ml_coeff) %>% dplyr::rename(CLAY_IR =
```

```
## Joining, by = c("IDEN", "IDEN_Method_treatment", "Method", "Treatment", "Method_treatment")
```

```r
pred_set6_C <- prediction(mod_set6B_C, testing_coeff, data_ml_coeff) %>% dplyr::rename(C_IR = "predict(
```

```
## Joining, by = c("IDEN", "IDEN_Method_treatment", "Method", "Treatment", "Method_treatment")
```

```r
# Set7
## All features
pred_set7all_ilr1 <- prediction(mod_set7all_ilr1, testing_feat, data_ml_feat) %>% dplyr::rename(ilr1_IR
```

```
## Joining, by = c("IDEN", "IDEN_Method_treatment", "Method", "Treatment", "Method_treatment")
```

```r
pred_set7all_ilr2 <- prediction(mod_set7all_ilr2, testing_feat, data_ml_feat) %>% dplyr::rename(ilr2_IR
```

```
## Joining, by = c("IDEN", "IDEN_Method_treatment", "Method", "Treatment", "Method_treatment")
```

```r
pred_set7all_ilr3 <- prediction(mod_set7all_ilr3, testing_feat, data_ml_feat) %>% dplyr::rename(ilr3_IR
```

```
## Joining, by = c("IDEN", "IDEN_Method_treatment", "Method", "Treatment", "Method_treatment")
```

```r
## Carbon
pred_set7c_ilr1 <- prediction(mod_set7c_ilr1, testing_feat, data_ml_feat) %>% dplyr::rename(ilr1_IR = "
```

```
## Joining, by = c("IDEN", "IDEN_Method_treatment", "Method", "Treatment", "Method_treatment")
```

```r
pred_set7c_ilr2 <- prediction(mod_set7c_ilr2, testing_feat, data_ml_feat) %>% dplyr::rename(ilr2_IR = "
```

```
## Joining, by = c("IDEN", "IDEN_Method_treatment", "Method", "Treatment", "Method_treatment")
```

```r
pred_set7c_ilr3 <- prediction(mod_set7c_ilr3, testing_feat, data_ml_feat) %>% dplyr::rename(ilr3_IR = "
```

```
## Joining, by = c("IDEN", "IDEN_Method_treatment", "Method", "Treatment", "Method_treatment")
```

```r
## Density
pred_set7d_ilr1 <- prediction(mod_set7d_ilr1, testing_feat, data_ml_feat) %>% dplyr::rename(ilr1_IR = "
```

```
## Joining, by = c("IDEN", "IDEN_Method_treatment", "Method", "Treatment", "Method_treatment")
```

```r
pred_set7d_ilr2 <- prediction(mod_set7d_ilr2, testing_feat, data_ml_feat) %>% dplyr::rename(ilr2_IR = "
```

```
## Joining, by = c("IDEN", "IDEN_Method_treatment", "Method", "Treatment", "Method_treatment")
```

```r
pred_set7d_ilr3 <- prediction(mod_set7d_ilr3, testing_feat, data_ml_feat) %>% dplyr::rename(ilr3_IR = "
```

```
## Joining, by = c("IDEN", "IDEN_Method_treatment", "Method", "Treatment", "Method_treatment")
```

```r
## pH
pred_set7ph_ilr1 <- prediction(mod_set7ph_ilr1, testing_feat, data_ml_feat) %>% dplyr::rename(ilr1_IR =
```

```
## Joining, by = c("IDEN", "IDEN_Method_treatment", "Method", "Treatment", "Method_treatment")
```

```r
pred_set7ph_ilr2 <- prediction(mod_set7ph_ilr2, testing_feat, data_ml_feat) %>% dplyr::rename(ilr2_IR =
```

```
## Joining, by = c("IDEN", "IDEN_Method_treatment", "Method", "Treatment", "Method_treatment")
```

```r
pred_set7ph_ilr3 <- prediction(mod_set7ph_ilr3, testing_feat, data_ml_feat) %>% dplyr::rename(ilr3_IR =
```

```
## Joining, by = c("IDEN", "IDEN_Method_treatment", "Method", "Treatment", "Method_treatment")
```

```r
## colors
pred_set7rgb_ilr1 <- prediction(mod_set7rgb_ilr1, testing_feat, data_ml_feat) %>% dplyr::rename(ilr1_IR
```

```
## Joining, by = c("IDEN", "IDEN_Method_treatment", "Method", "Treatment", "Method_treatment")
```

```r
pred_set7rgb_ilr2 <- prediction(mod_set7rgb_ilr2, testing_feat, data_ml_feat) %>% dplyr::rename(ilr2_IR
```

```
## Joining, by = c("IDEN", "IDEN_Method_treatment", "Method", "Treatment", "Method_treatment")
```

```r
pred_set7rgb_ilr3 <- prediction(mod_set7rgb_ilr3, testing_feat, data_ml_feat) %>% dplyr::rename(ilr3_IR
```

```
## Joining, by = c("IDEN", "IDEN_Method_treatment", "Method", "Treatment", "Method_treatment")
```

```r
## oxalate
pred_set7ox_ilr1 <- prediction(mod_set7ox_ilr1, testing_feat, data_ml_feat) %>% dplyr::rename(ilr1_IR =
```

```
## Joining, by = c("IDEN", "IDEN_Method_treatment", "Method", "Treatment", "Method_treatment")
```

```r
pred_set7ox_ilr2 <- prediction(mod_set7ox_ilr2, testing_feat, data_ml_feat) %>% dplyr::rename(ilr2_IR =
```

```
## Joining, by = c("IDEN", "IDEN_Method_treatment", "Method", "Treatment", "Method_treatment")
```

```r
pred_set7ox_ilr3 <- prediction(mod_set7ox_ilr3, testing_feat, data_ml_feat) %>% dplyr::rename(ilr3_IR =
```

```
## Joining, by = c("IDEN", "IDEN_Method_treatment", "Method", "Treatment", "Method_treatment")
```

```r
## M3
pred_set7m3_ilr1 <- prediction(mod_set7m3_ilr1, testing_feat, data_ml_feat) %>% dplyr::rename(ilr1_IR =
```

```
## Joining, by = c("IDEN", "IDEN_Method_treatment", "Method", "Treatment", "Method_treatment")
```

```r
pred_set7m3_ilr2 <- prediction(mod_set7m3_ilr2, testing_feat, data_ml_feat) %>% dplyr::rename(ilr2_IR =
```

```
## Joining, by = c("IDEN", "IDEN_Method_treatment", "Method", "Treatment", "Method_treatment")
```

```r
pred_set7m3_ilr3 <- prediction(mod_set7m3_ilr3, testing_feat, data_ml_feat) %>% dplyr::rename(ilr3_IR =
```

```
## Joining, by = c("IDEN", "IDEN_Method_treatment", "Method", "Treatment", "Method_treatment")
```

```r
## No features
pred_set7no_ilr1 <- prediction(mod_set7no_ilr1, testing_feat, data_ml_feat) %>% dplyr::rename(ilr1_IR =
```

```
## Joining, by = c("IDEN", "IDEN_Method_treatment", "Method", "Treatment", "Method_treatment")
```

```r
pred_set7no_ilr2 <- prediction(mod_set7no_ilr2, testing_feat, data_ml_feat) %>% dplyr::rename(ilr2_IR =
```

```
## Joining, by = c("IDEN", "IDEN_Method_treatment", "Method", "Treatment", "Method_treatment")
```

```r
pred_set7no_ilr3 <- prediction(mod_set7no_ilr3, testing_feat, data_ml_feat) %>% dplyr::rename(ilr3_IR =
```

```
## Joining, by = c("IDEN", "IDEN_Method_treatment", "Method", "Treatment", "Method_treatment")
```

```r
# Set8
## Sieving
pred_sieve <- as.data.frame(predict(mod_set8_SAND, testing_sieve))

result_sieve <- pred_sieve %>%
  cbind2(testing_sieve[, c("IDEN", "IDEN_Method_treatment", "Method", "Treatment", "Method_treatment")]
  left_join(., data_ml_sieve[, c("IDEN", "IDEN_Method_treatment", "Method", "Treatment", "Method_treatm
```

```
## Joining, by = c("IDEN", "IDEN_Method_treatment", "Method", "Treatment", "Method_treatment")
```

```r
pred_set8_SAND <- result_sieve %>% dplyr::rename(SAND_IR = "predict(mod_set8_SAND, testing_sieve)")

## Bouyoucos 2-points 2-h
testing_sieve_bouy$Method <- recode(testing_sieve_bouy$Method, "Bouyoucos 2-point 2-h" = 1, "Bouyoucos m
testing_sieve_bouy$Treatment <- recode(testing_sieve_bouy$Treatment, "No peroxide" = 1, "Peroxide" = 2,

pred_sieve_bouy <- as.data.frame(predict(mod_set8bouy_SAND, testing_sieve_bouy))
testing_sieve_bouy$Method <- recode(testing_sieve_bouy$Method, "1" = "Bouyoucos 2-point 2-h", "2" = "Bou
testing_sieve_bouy$Method <- as.character(testing_sieve_bouy$Method)
testing_sieve_bouy$Treatment <- recode(testing_sieve_bouy$Treatment, "1" = "No peroxide", "2" = "Peroxid
testing_sieve_bouy$Treatment <- as.character(testing_sieve_bouy$Treatment)

result_sieve_bouy <- pred_sieve_bouy %>%
  cbind2(testing_sieve_bouy[, c("IDEN", "IDEN_Method_treatment", "Method", "Treatment", "Method_treatmen
  left_join(., data_ml_sieve2[, c("IDEN", "IDEN_Method_treatment", "Method", "Treatment", "Method_treatm
```

```
## Joining, by = c("IDEN", "IDEN_Method_treatment", "Method", "Treatment", "Method_treatment")
```

```r
pred_set8_bouy_SAND <- result_sieve_bouy %>% dplyr::rename(SAND_IR = "predict(mod_set8bouy_SAND, testing
```

Tables of Sand, silt and clay percentages

Slope and intercept

Data organization

A dataframe object includes slope and intercept prediction results with their corresponding informations (identification, method and pre-treatment).

Coefficients transformated into sand, silt and clay percentages

Predictions

```r
# Predictions from NIR spectra
silt_clay_NIR <- exp(pred_set6_intercept$Intercept_coeff_NIR + log(40/60) * pred_set6_slope$Slope_coeff_
silt_clay_NIR <- ifelse(silt_clay_NIR > 100, 99, silt_clay_NIR)
time_coefficients_NIR <- pred_set6_intercept %>%
  left_join(., pred_set6_slope)
```

```
## Joining, by = c("IDEN", "IDEN_Method_treatment", "Method", "Treatment", "Method_treatment", "ilr1",
```

```
time_coefficients_NIR$CLAY_IR <- exp(time_coefficients_NIR$Intercept_coeff_NIR + log(120) * time_coeffi
time_coefficients_NIR$SILT_IR <- silt_clay_NIR - time_coefficients_NIR$CLAY_IR
time_coefficients_NIR$SAND_IR <- 100 - silt_clay_NIR
time_coefficients_NIR$C_IR <- pred_set6_carbon$Carbon_coeff_NIR
slope_intercept <- time_coefficients_NIR %>% dplyr::select("IDEN", "Method", "Treatment", "SAND", "SILT
```

ilr transformations

We transform ilr into sand, silt and clay percentages.

```
balance <- function(pred_test_1, pred_test_2, pred_test_3) {
  bal_pred <- data.frame(pred_test_1[, c("ilr1_IR")], pred_test_2[, c("ilr2_IR")], pred_test_3[, c("ilr
  comp_pred <- unclass(ilrInv(bal_pred, V=gsi.buildilrBase(t(sbp_texture_q1))))
  colnames(comp_pred) <- c('CLAY_IR', 'SILT_IR', 'SAND_IR', 'C_IR')
  comp_pred <- comp_pred %>%
    as.data.frame(.) %>%
    dplyr::mutate(CLAY_IR = CLAY_IR/(1-C_IR)*100, SILT_IR = SILT_IR/(1-C_IR)*100, SAND_IR = SAND_IR/(1-
    cbind2(., pred_test_1) %>%
    left_join(., pred_test_2) %>%
    left_join(., pred_test_3) %>%
    dplyr::select("IDEN", "Method", "Treatment", "SAND", "SILT", "CLAY", "C_perc", "SAND_IR", "SILT_IR"
    as.tibble(.)
return(comp_pred)
}
```

We transform ilr results to sand, silt, clay percentages.

```
balance_laser <- balance(pred_set1_ilr1, pred_set1_ilr2, pred_set1_ilr3) %>% mutate(Prediction = "Set1_
```

```
## Joining, by = c("IDEN", "IDEN_Method_treatment", "Method", "Treatment", "Method_treatment", "ilr1",
## Joining, by = c("IDEN", "IDEN_Method_treatment", "Method", "Treatment", "Method_treatment", "ilr1",
```

```
balance_tot <- balance(pred_set2_ilr1, pred_set2_ilr2, pred_set2_ilr3) %>% mutate(Prediction = "Set2_il
```

```
## Joining, by = c("IDEN", "IDEN_Method_treatment", "Method", "Treatment", "Method_treatment", "ilr1",
## Joining, by = c("IDEN", "IDEN_Method_treatment", "Method", "Treatment", "Method_treatment", "ilr1",
```

```
balance_multi <- balance(pred_set3A_ilr1, pred_set3A_ilr2, pred_set3A_ilr3) %>% mutate(Prediction = "Se
```

```
## Joining, by = c("IDEN", "IDEN_Method_treatment", "Method", "Treatment", "Method_treatment", "ilr1",
## Joining, by = c("IDEN", "IDEN_Method_treatment", "Method", "Treatment", "Method_treatment", "ilr1",
```

```
balance_two <- balance(pred_set3B_ilr1, pred_set3B_ilr2, pred_set3B_ilr3) %>% mutate(Prediction = "Set3
```

```
## Joining, by = c("IDEN", "IDEN_Method_treatment", "Method", "Treatment", "Method_treatment", "ilr1",
## Joining, by = c("IDEN", "IDEN_Method_treatment", "Method", "Treatment", "Method_treatment", "ilr1",
```

```r
balance_MIR <- balance(pred_set4A_ilr1, pred_set4A_ilr2, pred_set4A_ilr3) %>% mutate(Prediction = "Set4
```

```
## Joining, by = c("IDEN", "IDEN_Method_treatment", "Method", "Treatment", "Method_treatment", "ilr1",
## Joining, by = c("IDEN", "IDEN_Method_treatment", "Method", "Treatment", "Method_treatment", "ilr1",
```

```r
balance_NIR <- balance(pred_set4B_ilr1, pred_set4B_ilr2, pred_set4B_ilr3) %>% mutate(Prediction = "Set4
```

```
## Joining, by = c("IDEN", "IDEN_Method_treatment", "Method", "Treatment", "Method_treatment", "ilr1",
## Joining, by = c("IDEN", "IDEN_Method_treatment", "Method", "Treatment", "Method_treatment", "ilr1",
```

```r
balance_NIR4X <- balance(pred_set5A_ilr1, pred_set5A_ilr2, pred_set5A_ilr3) %>% mutate(Prediction = "Se
```

```
## Joining, by = c("IDEN", "IDEN_Method_treatment", "Method", "Treatment", "Method_treatment", "ilr1",
## Joining, by = c("IDEN", "IDEN_Method_treatment", "Method", "Treatment", "Method_treatment", "ilr1",
```

```r
balance_NIR2X <- balance(pred_set5B_ilr1, pred_set5B_ilr2, pred_set5B_ilr3) %>% mutate(Prediction = "Se
```

```
## Joining, by = c("IDEN", "IDEN_Method_treatment", "Method", "Treatment", "Method_treatment", "ilr1",
## Joining, by = c("IDEN", "IDEN_Method_treatment", "Method", "Treatment", "Method_treatment", "ilr1",
```

```r
balance_coeff <- balance(pred_set6_ilr1, pred_set6_ilr2, pred_set6_ilr3) %>% mutate(Prediction = "Set6_
```

```
## Joining, by = c("IDEN", "IDEN_Method_treatment", "Method", "Treatment", "Method_treatment", "ilr1",
## Joining, by = c("IDEN", "IDEN_Method_treatment", "Method", "Treatment", "Method_treatment", "ilr1",
```

```r
balance_featall <- balance(pred_set7all_ilr1, pred_set7all_ilr2, pred_set7all_ilr3) %>% mutate(Predicti
```

```
## Joining, by = c("IDEN", "IDEN_Method_treatment", "Method", "Treatment", "Method_treatment", "ilr1",
## Joining, by = c("IDEN", "IDEN_Method_treatment", "Method", "Treatment", "Method_treatment", "ilr1",
```

```r
balance_featc <- balance(pred_set7c_ilr1, pred_set7c_ilr2, pred_set7c_ilr3) %>% mutate(Prediction = "Se
```

```
## Joining, by = c("IDEN", "IDEN_Method_treatment", "Method", "Treatment", "Method_treatment", "ilr1",
## Joining, by = c("IDEN", "IDEN_Method_treatment", "Method", "Treatment", "Method_treatment", "ilr1",
```

```r
balance_featd <- balance(pred_set7d_ilr1, pred_set7d_ilr2, pred_set7d_ilr3) %>% mutate(Prediction = "Se
```

```
## Joining, by = c("IDEN", "IDEN_Method_treatment", "Method", "Treatment", "Method_treatment", "ilr1",
## Joining, by = c("IDEN", "IDEN_Method_treatment", "Method", "Treatment", "Method_treatment", "ilr1",
```

```r
balance_featph <- balance(pred_set7ph_ilr1, pred_set7ph_ilr2, pred_set7ph_ilr3) %>% mutate(Prediction =
```

```
## Joining, by = c("IDEN", "IDEN_Method_treatment", "Method", "Treatment", "Method_treatment", "ilr1",
## Joining, by = c("IDEN", "IDEN_Method_treatment", "Method", "Treatment", "Method_treatment", "ilr1",
```

```
balance_featrgb <- balance(pred_set7rgb_ilr1, pred_set7rgb_ilr2, pred_set7rgb_ilr3) %>% mutate(Predicti
```

```
## Joining, by = c("IDEN", "IDEN_Method_treatment", "Method", "Treatment", "Method_treatment", "ilr1",
## Joining, by = c("IDEN", "IDEN_Method_treatment", "Method", "Treatment", "Method_treatment", "ilr1",
```

```
balance_featox <- balance(pred_set7ox_ilr1, pred_set7ox_ilr2, pred_set7ox_ilr3) %>% mutate(Prediction =
```

```
## Joining, by = c("IDEN", "IDEN_Method_treatment", "Method", "Treatment", "Method_treatment", "ilr1",
## Joining, by = c("IDEN", "IDEN_Method_treatment", "Method", "Treatment", "Method_treatment", "ilr1",
```

```
balance_featm3 <- balance(pred_set7m3_ilr1, pred_set7m3_ilr2, pred_set7m3_ilr3) %>% mutate(Prediction =
```

```
## Joining, by = c("IDEN", "IDEN_Method_treatment", "Method", "Treatment", "Method_treatment", "ilr1",
## Joining, by = c("IDEN", "IDEN_Method_treatment", "Method", "Treatment", "Method_treatment", "ilr1",
```

```
balance_featno <- balance(pred_set7no_ilr1, pred_set7no_ilr2, pred_set7no_ilr3) %>% mutate(Prediction =
```

```
## Joining, by = c("IDEN", "IDEN_Method_treatment", "Method", "Treatment", "Method_treatment", "ilr1",
## Joining, by = c("IDEN", "IDEN_Method_treatment", "Method", "Treatment", "Method_treatment", "ilr1",
```

Group all results

We join all results and compare each other using linear graphics.

```
perc_set1 <- pred_set1_SAND %>%
  left_join(., pred_set1_SILT) %>%
  left_join(., pred_set1_CLAY) %>%
  left_join(., pred_set1_C) %>%
  dplyr::select("IDEN", "Method", "Treatment", "SAND", "SILT", "CLAY", "C_perc", "SAND_IR", "SILT_IR",
```

```
## Joining, by = c("IDEN", "IDEN_Method_treatment", "Method", "Treatment", "Method_treatment", "ilr1",
## Joining, by = c("IDEN", "IDEN_Method_treatment", "Method", "Treatment", "Method_treatment", "ilr1",
## Joining, by = c("IDEN", "IDEN_Method_treatment", "Method", "Treatment", "Method_treatment", "ilr1",
```

```
perc_set2 <- pred_set2_SAND %>%
  left_join(., pred_set2_SILT) %>%
  left_join(., pred_set2_CLAY) %>%
  left_join(., pred_set2_C) %>%
  dplyr::select("IDEN", "Method", "Treatment", "SAND", "SILT", "CLAY", "C_perc", "SAND_IR", "SILT_IR",
```

```
## Joining, by = c("IDEN", "IDEN_Method_treatment", "Method", "Treatment", "Method_treatment", "ilr1",
## Joining, by = c("IDEN", "IDEN_Method_treatment", "Method", "Treatment", "Method_treatment", "ilr1",
## Joining, by = c("IDEN", "IDEN_Method_treatment", "Method", "Treatment", "Method_treatment", "ilr1",
```

```
perc_set3A <- pred_set3A_SAND %>%
  left_join(., pred_set3A_SILT) %>%
  left_join(., pred_set3A_CLAY) %>%
  left_join(., pred_set3A_C) %>%
  dplyr::select("IDEN", "Method", "Treatment", "SAND", "SILT", "CLAY", "C_perc", "SAND_IR", "SILT_IR",
```

```
## Joining, by = c("IDEN", "IDEN_Method_treatment", "Method", "Treatment", "Method_treatment", "ilr1",
## Joining, by = c("IDEN", "IDEN_Method_treatment", "Method", "Treatment", "Method_treatment", "ilr1",
## Joining, by = c("IDEN", "IDEN_Method_treatment", "Method", "Treatment", "Method_treatment", "ilr1",
```

```
perc_set3B <- pred_set3B_SAND %>%
  left_join(., pred_set3B_SILT) %>%
  left_join(., pred_set3B_CLAY) %>%
  left_join(., pred_set3B_C) %>%
  dplyr::select("IDEN", "Method", "Treatment", "SAND", "SILT", "CLAY", "C_perc", "SAND_IR", "SILT_IR",
```

```
## Joining, by = c("IDEN", "IDEN_Method_treatment", "Method", "Treatment", "Method_treatment", "ilr1",
## Joining, by = c("IDEN", "IDEN_Method_treatment", "Method", "Treatment", "Method_treatment", "ilr1",
## Joining, by = c("IDEN", "IDEN_Method_treatment", "Method", "Treatment", "Method_treatment", "ilr1",
```

```
perc_set4A <- pred_set4A_SAND %>%
  left_join(., pred_set4A_SILT) %>%
  left_join(., pred_set4A_CLAY) %>%
  left_join(., pred_set4A_C) %>%
  dplyr::select("IDEN", "Method", "Treatment", "SAND", "SILT", "CLAY", "C_perc", "SAND_IR", "SILT_IR",
```

```
## Joining, by = c("IDEN", "IDEN_Method_treatment", "Method", "Treatment", "Method_treatment", "ilr1",
## Joining, by = c("IDEN", "IDEN_Method_treatment", "Method", "Treatment", "Method_treatment", "ilr1",
## Joining, by = c("IDEN", "IDEN_Method_treatment", "Method", "Treatment", "Method_treatment", "ilr1",
```

```
perc_set4B <- pred_set4B_SAND %>%
  left_join(., pred_set4B_SILT) %>%
  left_join(., pred_set4B_CLAY) %>%
  left_join(., pred_set4B_C) %>%
  dplyr::select("IDEN", "Method", "Treatment", "SAND", "SILT", "CLAY", "C_perc", "SAND_IR", "SILT_IR",
```

```
## Joining, by = c("IDEN", "IDEN_Method_treatment", "Method", "Treatment", "Method_treatment", "ilr1",
## Joining, by = c("IDEN", "IDEN_Method_treatment", "Method", "Treatment", "Method_treatment", "ilr1",
## Joining, by = c("IDEN", "IDEN_Method_treatment", "Method", "Treatment", "Method_treatment", "ilr1",
```

```
perc_set5A <- pred_set5A_SAND %>%
  left_join(., pred_set5A_SILT) %>%
  left_join(., pred_set5A_CLAY) %>%
  left_join(., pred_set5A_C) %>%
  dplyr::select("IDEN", "Method", "Treatment", "SAND", "SILT", "CLAY", "C_perc", "SAND_IR", "SILT_IR",
```

```
## Joining, by = c("IDEN", "IDEN_Method_treatment", "Method", "Treatment", "Method_treatment", "ilr1",
## Joining, by = c("IDEN", "IDEN_Method_treatment", "Method", "Treatment", "Method_treatment", "ilr1",
## Joining, by = c("IDEN", "IDEN_Method_treatment", "Method", "Treatment", "Method_treatment", "ilr1",
```

```
perc_set5B <- pred_set5B_SAND %>%
  left_join(., pred_set5B_SILT) %>%
  left_join(., pred_set5B_CLAY) %>%
  left_join(., pred_set5B_C) %>%
  dplyr::select("IDEN", "Method", "Treatment", "SAND", "SILT", "CLAY", "C_perc", "SAND_IR", "SILT_IR",
```

```
## Joining, by = c("IDEN", "IDEN_Method_treatment", "Method", "Treatment", "Method_treatment", "ilr1",
## Joining, by = c("IDEN", "IDEN_Method_treatment", "Method", "Treatment", "Method_treatment", "ilr1",
## Joining, by = c("IDEN", "IDEN_Method_treatment", "Method", "Treatment", "Method_treatment", "ilr1",
```

```
perc_set6 <- pred_set6_SAND %>%
  left_join(., pred_set6_SILT) %>%
  left_join(., pred_set6_CLAY) %>%
  left_join(., pred_set6_C) %>%
  dplyr::select("IDEN", "Method", "Treatment", "SAND", "SILT", "CLAY", "C_perc", "SAND_IR", "SILT_IR",
```

```
## Joining, by = c("IDEN", "IDEN_Method_treatment", "Method", "Treatment", "Method_treatment", "ilr1",
## Joining, by = c("IDEN", "IDEN_Method_treatment", "Method", "Treatment", "Method_treatment", "ilr1",
## Joining, by = c("IDEN", "IDEN_Method_treatment", "Method", "Treatment", "Method_treatment", "ilr1",
```

```
perc_set6_SI <- as.data.frame(slope_intercept) %>%
  dplyr::select("IDEN", "Method", "Treatment", "SAND", "SILT", "CLAY", "C_perc", "SAND_IR", "SILT_IR",

  perc_set8 <- pred_set8_SAND %>%
  mutate(SILT = NA, CLAY = NA, C_perc= NA, SILT_IR = NA, CLAY_IR = NA, C_IR = NA) %>%
  dplyr::select("IDEN", "Method", "Treatment", "SAND", "SILT", "CLAY", "C_perc", "SAND_IR", "SILT_IR",

perc_set8B <- pred_set8_bouy_SAND %>%
  mutate(SILT = NA, CLAY = NA, C_perc= NA, SILT_IR = NA, CLAY_IR = NA, C_IR = NA) %>%
  dplyr::select("IDEN", "Method", "Treatment", "SAND", "SILT", "CLAY", "C_perc", "SAND_IR", "SILT_IR",
```

```
percentages_test <- balance_tot %>%
  rbind.data.frame(., balance_featall) %>%
  rbind.data.frame(., balance_featno) %>%
  rbind.data.frame(., balance_featc) %>%
  rbind.data.frame(., balance_featd) %>%
  rbind.data.frame(., balance_featph) %>%
  rbind.data.frame(., balance_featrgb) %>%
  rbind.data.frame(., balance_featox) %>%
  rbind.data.frame(., balance_featm3) %>%
  rbind.data.frame(., balance_coeff) %>%
  rbind.data.frame(., balance_MIR) %>%
  rbind.data.frame(., balance_NIR) %>%
  rbind.data.frame(., balance_laser) %>%
  rbind.data.frame(., balance_NIR4X) %>%
  rbind.data.frame(., balance_NIR2X) %>%
  rbind.data.frame(., balance_multi) %>%
  rbind.data.frame(., balance_two) %>%
  rbind.data.frame(., perc_set1) %>%
  rbind.data.frame(., perc_set2) %>%
  rbind.data.frame(., perc_set3A) %>%
  rbind.data.frame(., perc_set3B) %>%
  rbind.data.frame(., perc_set4A) %>%
  rbind.data.frame(., perc_set4B) %>%
  rbind.data.frame(., perc_set5A) %>%
  rbind.data.frame(., perc_set5B) %>%
  rbind.data.frame(., perc_set6) %>%
  rbind.data.frame(., perc_set6_SI) %>%
```

```
  rbind.data.frame(., perc_set8) %>%
  rbind.data.frame(., perc_set8B)
```

We order names.

```
col_order <- c("IDEN", "CLAY_IR", "SILT_IR", "SAND_IR", "C_IR", "CLAY", "SILT", "SAND", "C_perc", "Predi
percentages_final <- percentages_test[, col_order]

df <- percentages_final
df$Prediction <- factor(df$Prediction,
                        levels = c("Set1_ilr", "Set1_%", "Set2_ilr", "Set2_%",  "Set3_Multi-point_ilr",
```

Visualization

We used this reference for plot configuration. Reference: https://rstudio-pubs-static.s3.amazonaws.com/213536_d4b3975ee92b43af8671057ccefb90c7.html

SAND percentages predictions vs laboratory results

Data organization

We select columns, eliminate Nas and change types.

```
df_SAND <- df[, c("IDEN", "SAND", "SAND_IR", "Prediction")]
df_SAND <- df_SAND[complete.cases(df_SAND),]
df_SAND_ratio <- df_SAND
df_SAND_ratio$IDEN <- as.numeric(df_SAND_ratio$IDEN)
df_SAND_ratio <- dplyr::select(df_SAND_ratio, IDEN, Prediction, SAND, SAND_IR)
max <- max(df_SAND_ratio$SAND)
min <- min(df_SAND_ratio$SAND)
```

Statistics calculations

```
df_SAND_ratio <-  df_SAND_ratio %>%
  as.data.frame(.) %>%
  group_by(Prediction) %>%
  dplyr::mutate(SEP = sqrt((sum((SAND-SAND_IR)^2))/5422)) %>%
  dplyr::mutate(SD =  sqrt(mean(SAND^2)-mean(SAND)^2)) %>%
  dplyr::mutate(RPD = SD/SEP) %>%
  dplyr::mutate(RER = (max - min)/SEP)
write.table(df_SAND_ratio, file = "SAND_ratio.txt", sep = ",", quote = FALSE, row.names = F)
```

Formulas combination

https://stackoverflow.com/questions/43123462/how-to-obtain-rmse-out-of-lm-result

```
regression_SAND = function(df_SAND){
  #setting the regression function.
  reg_fun<-lm(formula=df_SAND$SAND ~ df_SAND$SAND_IR) #regression function
  #getting the slope, intercept, R square and adjusted R squared of
  #the regression function (with 3 decimals).
  slope<-round(coef(reg_fun)[2],3)
  intercept<-round(coef(reg_fun)[1],3)
  R2<-round(as.numeric(summary(reg_fun)[8]),3)
  R2.Adj<-round(as.numeric(summary(reg_fun)[9]),3)
  RSS <- c(crossprod(reg_fun$residuals)) #Residual sum of squares
  MSE <- RSS / length(reg_fun$residuals) #Mean squared error
  RMSE <- sqrt(MSE) #Root MSE
  sig2 <- RSS / reg_fun$df.residual #Pearson estimated residual variance
  c(slope,intercept,R2,R2.Adj,RMSE)
}
regressions_data_SAND <- plyr::ddply(df_SAND,"Prediction",regression_SAND)
colnames(regressions_data_SAND) <- c ("Group_tested", "slope","intercept","R2","R2.Adj", "RMSE")
```

Vizualisation

All graphs featuring sand percentages are summarized.

```
gg_SAND <- ggplot(data = df_SAND, mapping = aes(x = SAND_IR, y = SAND)) +
  facet_wrap(~Prediction, ncol=2) +
  geom_point(alpha = 0.5) +
  xlab("Sand determined by corresponding laboratory analysis (%)") +
  ylab("Sand predicted by infrared spectroscopy methods (%)") +
  #ggtitle("Comparison of laboratory analysis sand percentage vs other laboratory tests or infrared met
  #theme_classic() +
  theme(plot.title = element_text(lineheight=.8, face="bold")) +
  geom_abline(aes(intercept = 0, slope = 1)) +
  geom_smooth(method = lm) #+
#geom_count() #+
#geom_label(data=regressions_data, inherit.aes=FALSE, aes(x = 1.2, y = 32,
ggsave("Methods_sand.jpg", width = 15, height = 15)
```

SILT percentages

Data organization

We select columns, eliminate Nas and change types.

```
df_SILT <- df[, c("IDEN", "SILT", "SILT_IR", "Prediction")]
df_SILT <- df_SILT[complete.cases(df_SILT),]
df_SILT_ratio <- df_SILT
df_SILT_ratio$IDEN <- as.numeric(df_SILT_ratio$IDEN)
df_SILT_ratio <- dplyr::select(df_SILT_ratio, IDEN, Prediction, SILT, SILT_IR)
max <- max(df_SILT_ratio$SILT)
min <- min(df_SILT_ratio$SILT)
```

Statistics calculations

```
df_SILT_ratio <-  df_SILT_ratio %>%
  as.data.frame(.) %>%
  group_by(Prediction) %>%
  dplyr::mutate(SEP = sqrt((sum((SILT-SILT_IR)^2))/5422)) %>%
  dplyr::mutate(SD =  sqrt(mean(SILT^2)-mean(SILT)^2)) %>%
  dplyr::mutate(RPD = SD/SEP) %>%
  dplyr::mutate(RER = (max - min)/SEP)
write.table(df_SILT_ratio, file = "SILT_ratio.txt", sep = ",", quote = FALSE, row.names = F)
```

Formulas combination

https://stackoverflow.com/questions/43123462/how-to-obtain-rmse-out-of-lm-result

```
regression_SILT=function(df_SILT){
  #setting the regression function.
  reg_fun<-lm(formula=df_SILT$SILT ~ df_SILT$SILT_IR) #regression function
  #getting the slope, intercept, R square and adjusted R squared of
  #the regression function (with 3 decimals).
  slope<-round(coef(reg_fun)[2],3)
  intercept<-round(coef(reg_fun)[1],3)
  R2<-round(as.numeric(summary(reg_fun)[8]),3)
  R2.Adj<-round(as.numeric(summary(reg_fun)[9]),3)
  RSS <- c(crossprod(reg_fun$residuals))
  MSE <- RSS / length(reg_fun$residuals)
  RMSE <- sqrt(MSE)
  sig2 <- RSS / reg_fun$df.residual
  c(slope,intercept,R2,R2.Adj,RMSE)
}

regressions_data_SILT <- plyr::ddply(df_SILT,"Prediction",regression_SILT)
colnames(regressions_data_SILT) <- c ("Group_tested", "slope","intercept","R2","R2.Adj", "RMSE")
#regressions_data_SILT$Target <- c("SILT")
```

Vizualisation

All graphs featuring silt percentages are summarized.

```
ggplot(data = df_SILT, mapping = aes(x = SILT_IR, y = SILT)) +
  facet_wrap(~Prediction, ncol=2) +
  geom_point(alpha = 0.5) +
  xlab("SILT determined by corresponding laboratory analysis (%)") +
  ylab("SILT predicted by infrared spectroscopy methods (%)") +
  #ggtitle("Comparison of laboratory analysis SILT percentage vs other laboratory tests or infrared met
  theme(plot.title = element_text(lineheight=.8, face="bold")) +
  geom_abline(aes(intercept = 0, slope = 1)) +
  geom_smooth(method = lm)
```

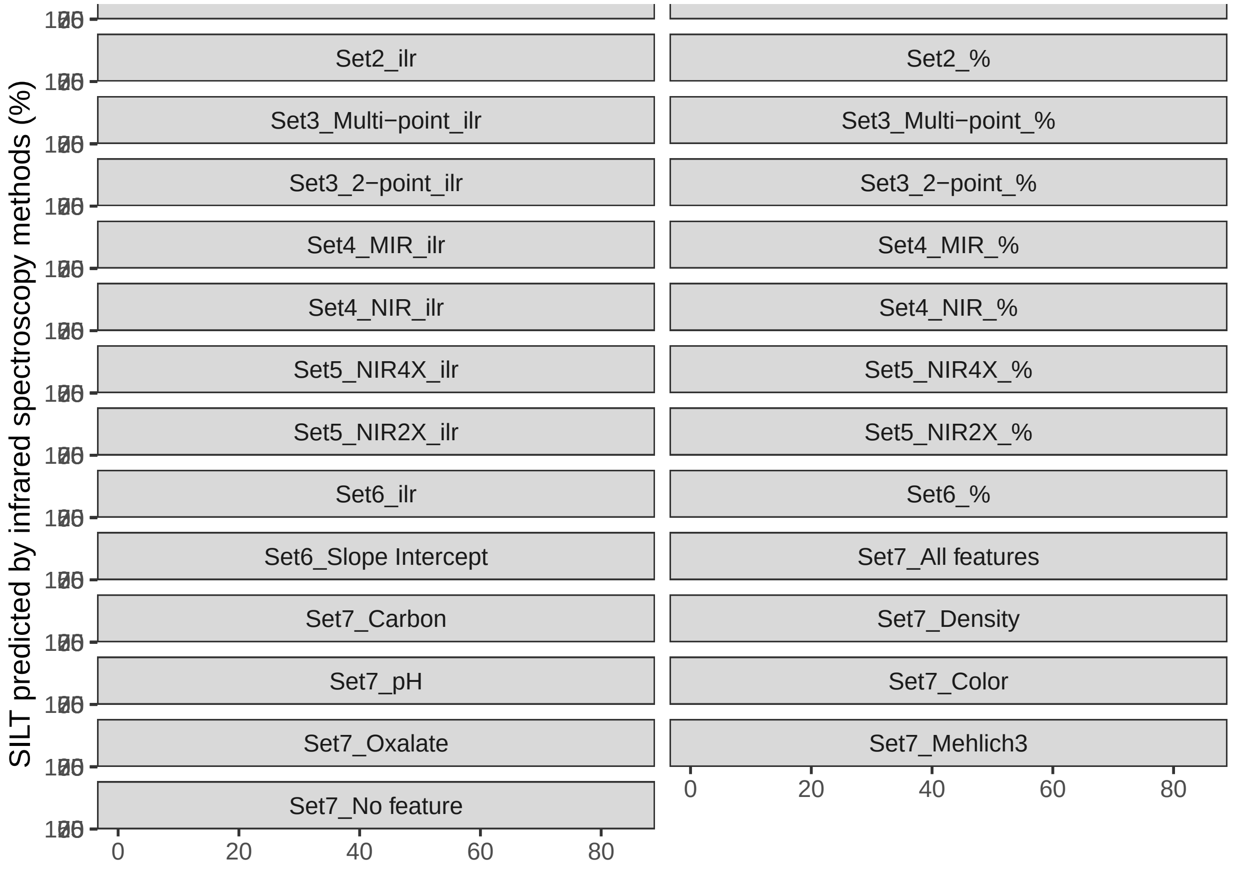


```
ggsave("Methods_silt.jpg", width = 15, height = 15)
```

CLAY percentages

Data organization

We select columns, eliminate Nas and change types.

```
df_CLAY <- df
df_CLAY <- df_CLAY[complete.cases(df_CLAY),]
df_CLAY_ratio <- df_CLAY
df_CLAY_ratio$IDEN <- as.numeric(df_CLAY_ratio$IDEN)
df_CLAY_ratio <- dplyr::select(df_CLAY_ratio, IDEN, Prediction, CLAY, CLAY_IR)
max <- max(df_CLAY_ratio$CLAY)
min <- min(df_CLAY_ratio$CLAY)
```

Statistics calculations

```
df_CLAY_ratio <-  df_CLAY_ratio %>%
  as.data.frame(.) %>%
  group_by(Prediction) %>%
  dplyr::mutate(SEP = sqrt((sum((CLAY-CLAY_IR)^2))/5422)) %>%
```

```
  dplyr::mutate(SD =  sqrt(mean(CLAY^2)-mean(CLAY)^2)) %>%
  dplyr::mutate(RPD = SD/SEP) %>%
  dplyr::mutate(RER = (max - min)/SEP)
write.table(df_CLAY_ratio, file = "CLAY_ratio.txt", sep = ",", quote = FALSE, row.names = F)
```

Formulas combination

https://stackoverflow.com/questions/43123462/how-to-obtain-rmse-out-of-lm-result

```
regression_CLAY=function(df_CLAY){
  #setting the regression function.
  reg_fun<-lm(formula=df_CLAY$CLAY ~ df_CLAY$CLAY_IR) #regression function
  #getting the slope, intercept, R square and adjusted R squared of
  #the regression function (with 3 decimals).
  slope<-round(coef(reg_fun)[2],3)
  intercept<-round(coef(reg_fun)[1],3)
  R2<-round(as.numeric(summary(reg_fun)[8]),3)
  R2.Adj<-round(as.numeric(summary(reg_fun)[9]),3)
  RSS <- c(crossprod(reg_fun$residuals))
  MSE <- RSS / length(reg_fun$residuals)
  RMSE <- sqrt(MSE)
  sig2 <- RSS / reg_fun$df.residual
  c(slope,intercept,R2,R2.Adj,RMSE)
}

regressions_data_CLAY <- plyr::ddply(df_CLAY,"Prediction",regression_CLAY)
colnames(regressions_data_CLAY) <- c("Group_tested", "slope","intercept","R2","R2.Adj", "RMSE")
#regressions_data_CLAY$Target <- c("CLAY")
```

Vizualisation

All graphs featuring silt percentages are summarized.

```
ggplot(data = df_CLAY, mapping = aes(x = CLAY_IR, y = CLAY)) +
  facet_wrap(~Prediction, ncol=2) +
  geom_point(alpha = 0.5) +
  xlab("CLAY determined by corresponding laboratory analysis (%)") +
  ylab("CLAY predicted by infrared spectroscopy methods (%)") +
  #ggtitle("Comparison of laboratory analysis CLAY percentage vs other laboratory tests or infrared met
  theme(plot.title = element_text(lineheight=.8, face="bold")) +
  geom_abline(aes(intercept = 0, slope = 1)) +
  geom_smooth(method = lm) #+
```

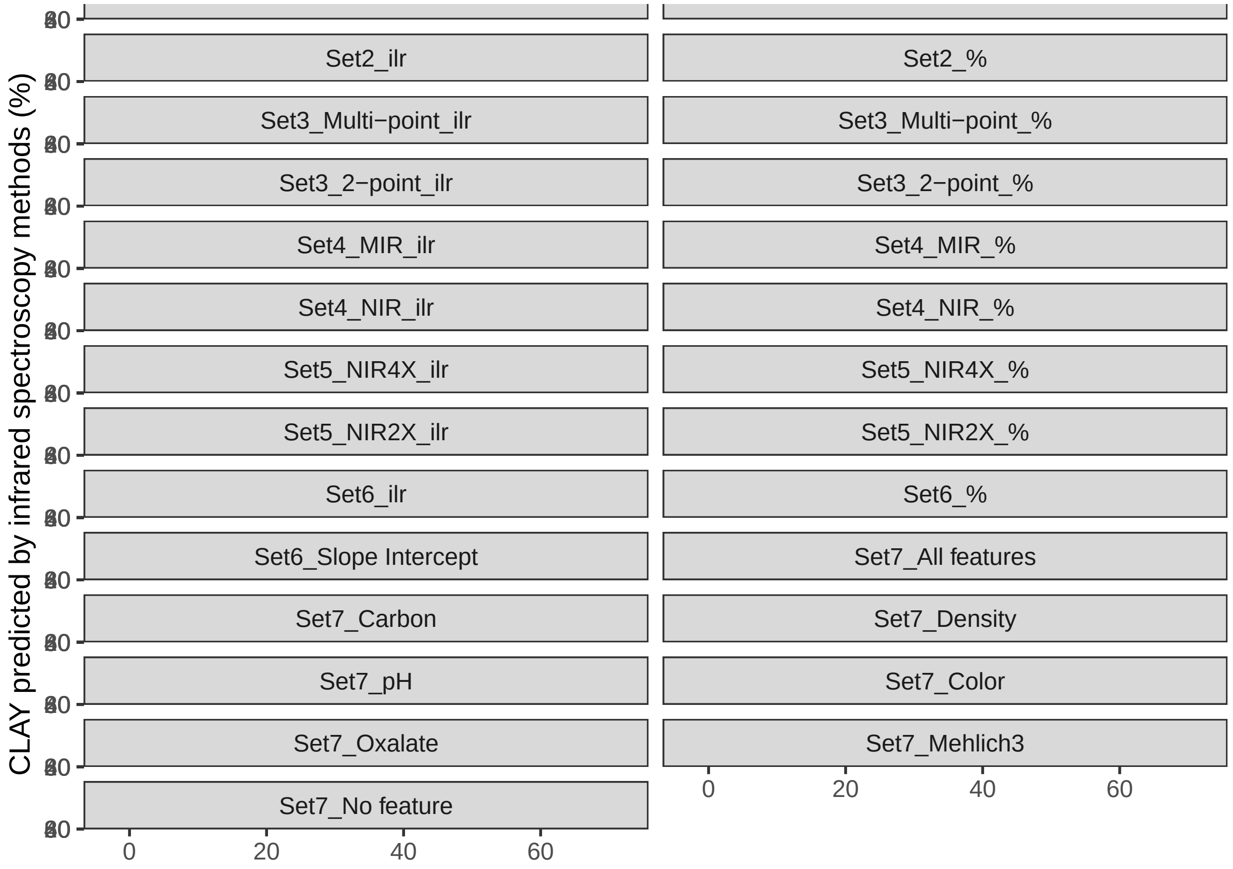


```
# geom_count()  #+
#geom_label(data=regressions_data, inherit.aes=FALSE, aes(x = 1.2, y = 32,
#label=paste("slope=",slope,","," ","intercept=",intercept,","," ","R^2=",R2,","," ","R^2.Adj=",R2.Adj)
names(c)
ggsave("Methods_clay.jpg", width = 15, height = 15)
```

Carbon percentages

Data organization

We select columns, eliminate Nas and change types.

```
df_C <- df
df_C <- df_C[complete.cases(df_C),]

df_C_ratio <- df_C
df_C_ratio$IDEN <- as.numeric(df_C_ratio$IDEN)
df_C_ratio <- dplyr::select(df_C_ratio, IDEN, Prediction, C_perc, C_IR)
max <- max(df_C_ratio$C_perc)
min <- min(df_C_ratio$C_perc)
```

Statistics calculations

```
df_C_ratio <-  df_C_ratio %>%
  as.data.frame(.) %>%
  group_by(Prediction) %>%
  dplyr::mutate(SEP = sqrt((sum((C_perc-C_IR)^2))/5422)) %>%
  dplyr::mutate(SD =  sqrt(mean(C_perc^2)-mean(C_perc)^2)) %>%
  dplyr::mutate(RPD = SD/SEP) %>%
  dplyr::mutate(RER = (max - min)/SEP)
write.table(df_C_ratio, file = "C_ratio.txt", sep = ",", quote = FALSE, row.names = F)
```

Formulas combination

https://stackoverflow.com/questions/43123462/how-to-obtain-rmse-out-of-lm-result

```
regression_C=function(df_C){
  #setting the regression function.
  reg_fun<-lm(formula=df_C$C_perc ~ df_C$C_IR) #regression function
  #getting the slope, intercept, R square and adjusted R squared of
  #the regression function (with 3 decimals).
  slope<-round(coef(reg_fun)[2],3)
  intercept<-round(coef(reg_fun)[1],3)
  R2<-round(as.numeric(summary(reg_fun)[8]),3)
  R2.Adj<-round(as.numeric(summary(reg_fun)[9]),3)
  RSS <- c(crossprod(reg_fun$residuals))
  MSE <- RSS / length(reg_fun$residuals)
  RMSE <- sqrt(MSE)
  sig2 <- RSS / reg_fun$df.residual
  c(slope,intercept,R2,R2.Adj,RMSE)
}

regressions_data_C <- plyr::ddply(df_C,"Prediction", regression_C)
colnames(regressions_data_C) <- c ("Group_tested", "slope","intercept","R2","R2.Adj", "RMSE")
#regressions_data_CLAY$Target <- c("C")
```

Vizualisation

All graphs featuring silt percentages are summarized.

```
ggplot(data = df_C, mapping = aes(x = C_IR, y = C_perc)) +
  facet_wrap(~Prediction, ncol=2) +
  geom_point(alpha = 0.5) +
  xlab("CLAY determined by corresponding laboratory analysis (%)") +
  ylab("CLAY predicted by infrared spectroscopy methods (%)") +
  #ggtitle("Comparison of laboratory analysis CLAY percentage vs other laboratory tests or infrared met
  theme(plot.title = element_text(lineheight=.8, face="bold")) +
  geom_abline(aes(intercept = 0, slope = 1)) +
  geom_smooth(method = lm) #+
```

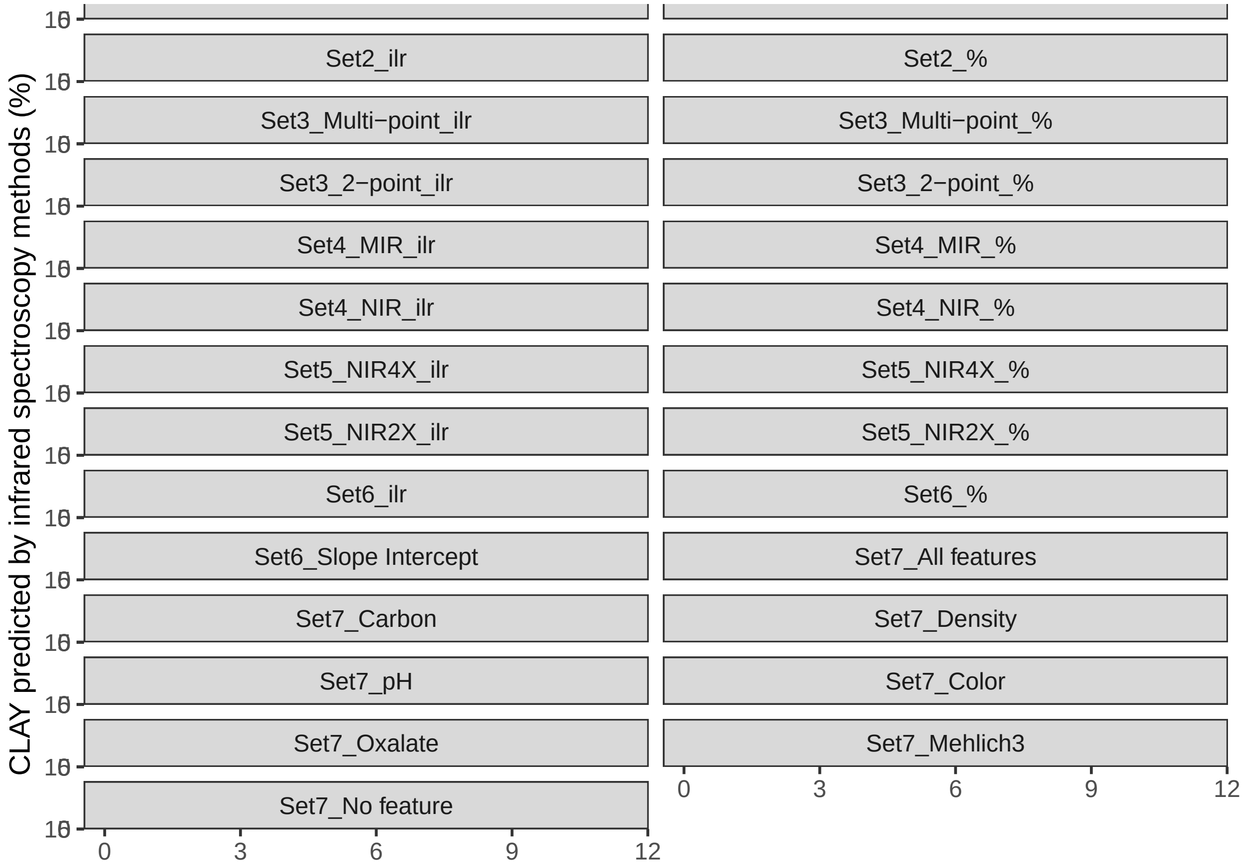


```
# geom_count()  #+
#geom_label(data=regressions_data, inherit.aes=FALSE, aes(x = 1.2, y = 32,
#label=paste("slope=",slope,",","  ","intercept=",intercept,",","  ","R^2=",R2,",","  ","R^2.Adj=",R2.Adj)
names(c)
```

```
## NULL
```

```
ggsave("Methods_c.jpg", width = 15, height = 15)
```

Results

Table

We summarize all results in a table.

```
regression_Bouy <- regressions_data_SAND %>%
  rename(SAND_slope = slope,  SAND_intercept = intercept, SAND_R2 = R2, SAND_R2.Adj = R2.Adj, SAND_RMSE
  left_join(., regressions_data_SILT, by = "Group_tested") %>%
  rename(SILT_slope = slope,  SILT_intercept = intercept, SILT_R2 = R2, SILT_R2.Adj = R2.Adj, SILT_RMSE
  left_join(., regressions_data_CLAY, by = "Group_tested") %>%
  rename(CLAY_slope = slope,  CLAY_intercept = intercept, CLAY_R2 = R2, CLAY_R2.Adj = R2.Adj, CLAY_RMSE
  left_join(., regressions_data_C, by = "Group_tested") %>%
  rename(C_slope = slope,  C_intercept = intercept, C_R2 = R2, C_R2.Adj = R2.Adj, C_RMSE = RMSE)
write.table(regression_Bouy, file = "regressions.txt", sep = ".", quote = FALSE, row.names = F)
#write.csv2(regression_Bouy, file = "regressions.csv", sep = ".", quote = FALSE, row.names = F)
```
